# Reactivity and Structure of a Bis-phenolate Niobium NHC Complex

**DOI:** 10.1021/acsorginorgau.2c00028

**Published:** 2022-12-05

**Authors:** Florian R. Neururer, Konstantin Huter, Michael Seidl, Stephan Hohloch

**Affiliations:** Faculty of Chemistry, Institute of Inorganic, General and Theoretical Chemistry, University of Innsbruck, Innrain 80-82, 6020 Innsbruck, Austria

**Keywords:** N-heterocyclic carbenes, early transition metals, niobium, NHC ligands, carbenes, imidos

## Abstract

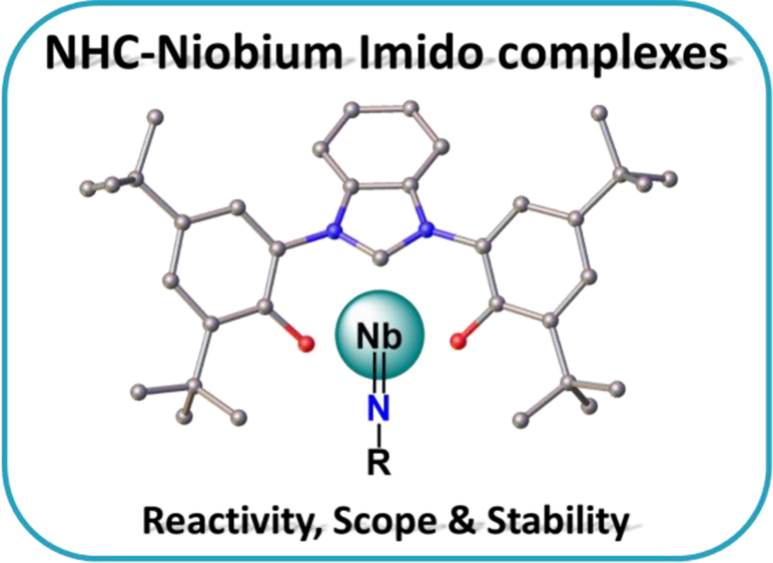

We report the facile synthesis of a rare niobium(V) imido NHC complex with a dianionic OCO-pincer benzimidazolylidene ligand (**L**^**1**^) with the general formula **[Nb**L**^**1**^(N**^***t***^**Bu)PyCl] 1-Py**. We achieved this by *in situ* deprotonation of the corresponding azolium salt **[H**_**3**_**L**^**1**^**][Cl]** and subsequent reaction with **[Nb(N**^***t***^**Bu)Py**_**2**_**Cl**_**3**_**]**. The pyridine ligand in **1-Py** can be removed by the addition of B(C_6_F_5_)_3_ as a strong Lewis acid leading to the formation of the pyridine-free complex **1**. In contrast to similar vanadium(V) complexes, complex **1-Py** was found to be a good precursor for various salt metathesis reactions, yielding a series of chalcogenido and pnictogenido complexes with the general formula **[NbL^**1**^(N**^***t***^**Bu)Py(EMes)]** (E = O (**2**), S (**3**), NH (**4**), and PH (**5**)). Furthermore, complex **1-Py** can be converted to alkyl complex (**6**) with 1 equiv of neosilyl lithium as a transmetallation agent. Addition of a second equivalent yields a new trianionic supporting ligand on the niobium center (**7**) in which the benzimidazolylidene ligand is alkylated at the former carbene carbon atom. The latter is an interesting chemically “noninnocent” feature of the benzimidazolylidene ligand potentially useful in catalysis and atom transfer reactions. Addition of mesityl lithium to **1-Py** gives the pyridine-free aryl complex **8**, which is stable toward “overarylation” by an additional equivalent of mesityl lithium. Electrochemical investigation revealed that complexes **1-Py** and **1** are inert toward reduction in dichloromethane but show two irreversible reduction processes in tetrahydrofuran as a solvent. However, using standard reduction agents, *e.g.*, KC_8_, K-mirror, and Na/Napht, no reduced products could be isolated. All complexes have been thoroughly studied by various techniques, including ^1^H-, ^13^C{^1^H}-, and ^1^H-^15^N HMBC NMR spectroscopy, IR spectroscopy, and X-ray diffraction analysis.

## Introduction

**Figure 1:**
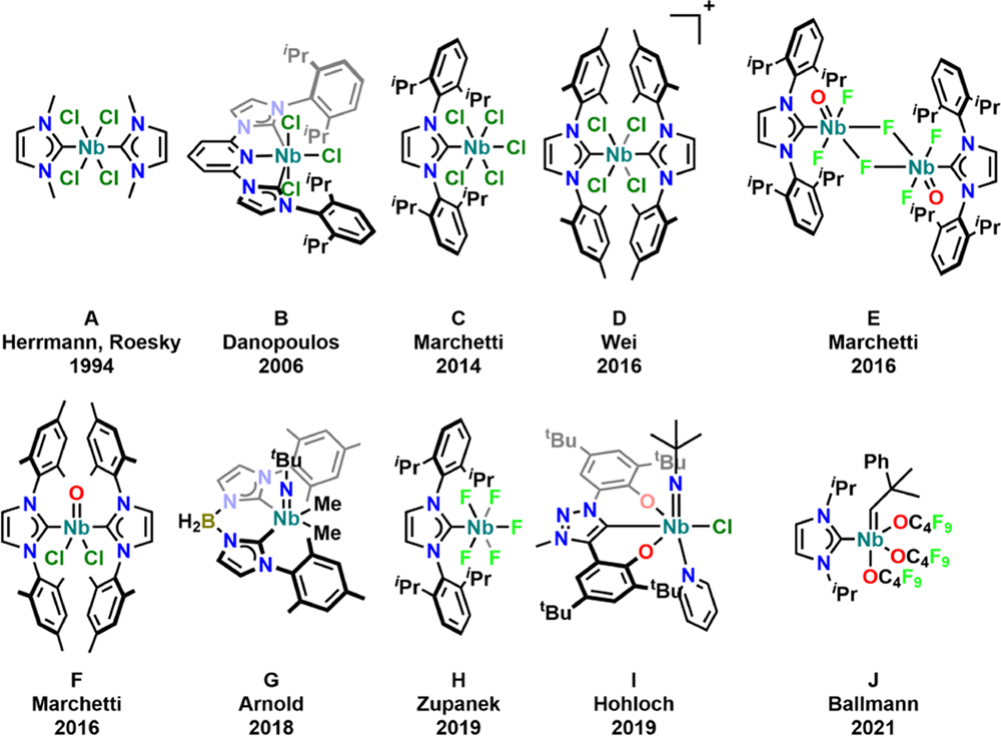
Selected examples of niobium NHC complexes reported so far.

Niobium-based complexes are of considerable interest,^1−3^*e.g.*, due to their potential catalytic applications in transformations such as group atom transfer^3−14^ and polymerization^6,15−20^ reactions as well as the (stoichiometric) activation of nitrogen^21−25^ or phosphorous.^26−31^ In this context, a variety of supporting ligands have been investigated, ranging from simple phenolates^24,32−34^ or amides,^11,23^ to bidentate BDI ligands^3,7,35−49^ and guinidinates^50−53^ or formamidinates^54^ over to tridentate pincer-type PNP^5,13,55,56^ ligands. In contrast, *N*-heterocyclic carbene-based ligands have been rarely used in niobium chemistry. Despite seminal work by Herrmann and Roesky (Figure 1A),^57^ who showed that high-valent niobium NHC complexes can be easily accessed, their chemistry stayed dormant for the past decades. However, the lack of systematic use of NHC in niobium chemistry is even more surprising since 10 years later Danopoulos showed (Figure 1B) that even low-valent niobium(III) complexes can be stabilized by a tridentate NHC ligand.^58^ Instead, it took another decade until this research area was slowly explored by Marchetti,^59−61^ Chao,^62^ and Zupanek^63^ and co-workers, showing that high-valent niobium NHC complexes (Figure 1C–F,H) can be easily accessed with monodentate imidazol-2-ylidene ligands. However, given the high instability of early-transition-metal NHC complexes resulting from nonideal orbital interactions,^64^ the tendency of Nb-NHC complexes to undergo hydrolysis is very high,^65^ prohibiting a detailed investigation of their reactivity or their use in catalysis. This difficulty was overcome by Arnold and co-workers, who used a borate-tethered anionic bis-NHC ligand^66−68^ (Figure 1G). This allowed the detailed exploration of the reactivity of these complexes, showing that the borate backbone was not as chemically innocent as initially expected, undergoing (among others) cycloaddition reactions with ketones and carbon monoxide.^69^ Notably, the latter also led to the reduction of the niobium(V) to a niobium(III) center. This unusual metal-based reduction behavior can be traced back to the reductive nature of the borate backbone which undergoes hydroboration. The redox-innocence of a niobium(V) NHC complex is further confirmed by the fact that Ballmann’s niobium NHC complex (Figure 1J) is inert under reductive conditions (hydrogen atmosphere) while its tantalum congener is not.^70^ However, except for Arnold and Ballmann’s work, no general overview of the reactivity, scope, and limitations of niobium NHC complexes has been reported so far and the chemistry of niobium NHC complexes is still in its infancy.

We recently started to investigate the coordination chemistry of phenolate-linked tridentate mesoionic and *N*-heterocyclic carbenes^71−74^ with a special emphasis on the OCO bis-phenolate benzimidazol-2-ylidene ligand (**L**^**1**^) originally introduced by Bellemin-Laponnaz and Bercaw.^75,76^ Due to the highly oxophilic nature of the early transition metals, the oxygen tethers enforce a strong interaction between the carbene ligand and the early transition metal. This led, among others,^77−87^ to the first example of an air- and moisture-stable dioxomolybdenum complex supported by an NHC ligand.^71^ This complex was further found to be a useful catalyst in the deoxygenation of nitroarenes.^73^ Furthermore, we recently investigated the coordination chemistry **L**^**1**^ toward high-valent vanadium(V).^74^ Here we extend our efforts in exploring the chemistry of early-transition-metal NHC complexes to the heavier congener of vanadium, namely, niobium.

**Scheme 1:**
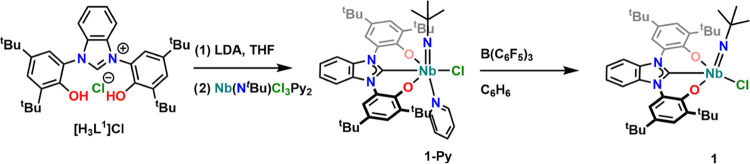
Synthesis of the Benzimidazol-2-ylidene Niobium Complexes **1-Py** and **1**

## Results and Discussion

**Figure 2:**
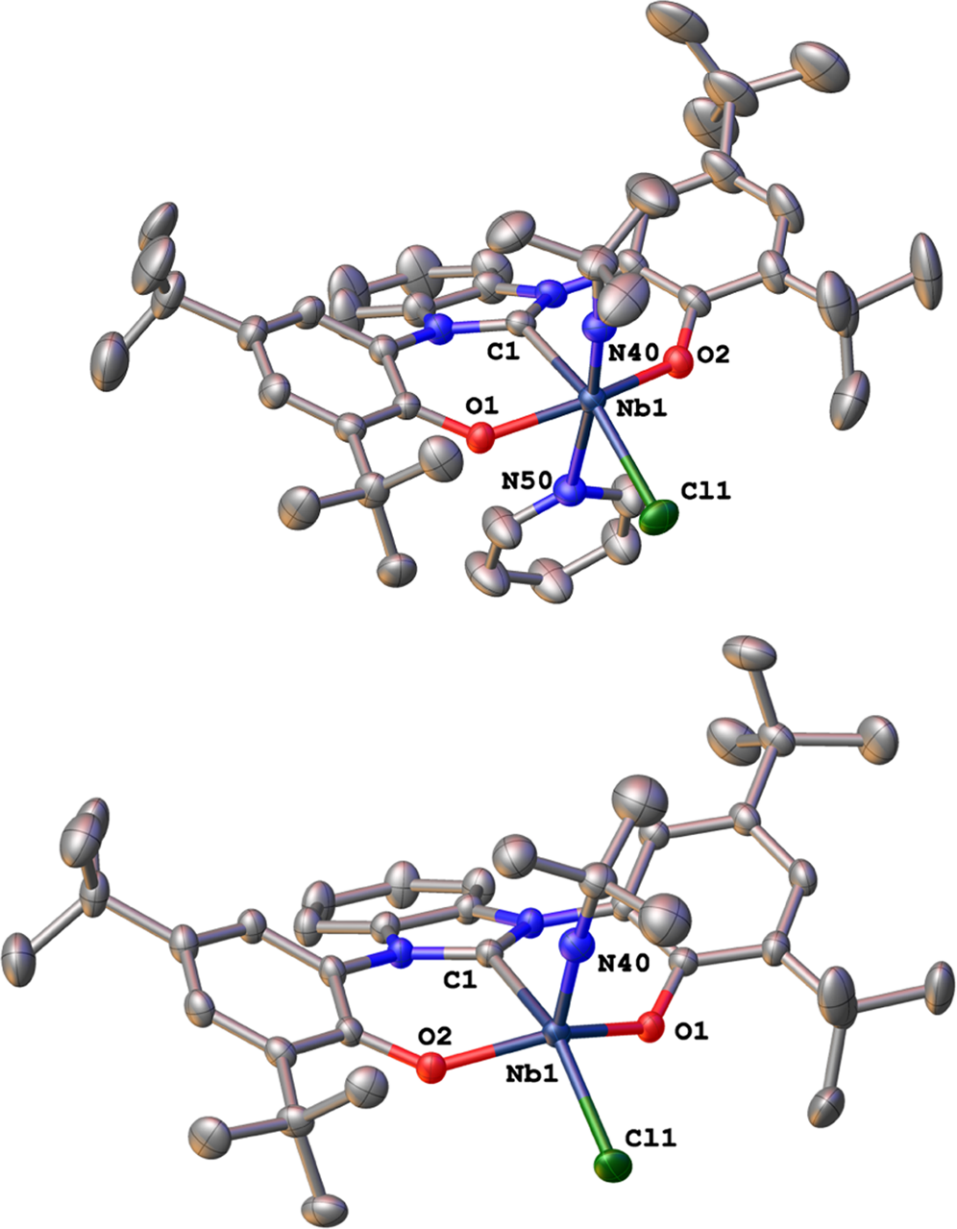
Molecular structure of the niobium benzimidazole-2-ylidene complexes **1-Py** (top) and **1** (bottom). Hydrogen atoms and solvent lattice molecules have been omitted for clarity. Only one of the two molecular units within the asymmetric unit is displayed. Ellipsoids are shown at a probability level of 50%.

Synthesis of the imido complex **1-Py** was achieved following a procedure recently reported by our group to synthesize the corresponding MIC complex **I** (MIC = mesoionic carbene; Figure 1).^72^ Addition of 3.3 equiv of lithium diisopropylamide (LDA) to a tetrahydrofuran (THF) solution of the proligand **[H**_**3**_**L**^**1**^**][Cl]** results in a bright green solution, which is then added to a yellow solution of the precursor **[Nb(N**^***t***^**Bu)Py**_**2**_**Cl**_**3**_**]** in THF. After the respective work-up, complex **1-Py** crystallizes readily from diethyl ether at −40 °C as pale-yellow blocks (65%, crystalline yield). The reaction seems to be moderately scalable and has been performed to yield up to 2 g of the desired complex **1-Py**. First evidence for the successful formation of the desired complex **1-Py** is indicated by the disappearance of the benzimidazolium 2-H proton and the two OH protons of the benzimidazolium salt **[H**_**3**_**L**^**1**^**][Cl]**. Additionally, ^1^H NMR spectroscopy reveals the presence of 1 equiv of pyridine. Indeed, even prolonged drying of the complex at elevated temperatures under high vacuum (60 °C, 10^–3^ mbar) does not result in the loss of the pyridine, suggesting its coordination toward the niobium center. Unfortunately, due to the large quadrupolar moment of the ^93^Nb center, no characteristic ^13^C{^1^H} resonance could be observed, even after prolonged measurement times at high-field NMR instruments. However, a shift of the imido *tert*-butyl protons from 1.48 ppm in **[Nb(N**^***t***^**Bu)Py**_**2**_**Cl**_**3**_**]** (Figure S1) to 0.93 ppm in complex **1-Py** (Figure S3) in the ^1^H NMR spectra of the corresponding complexes indicates a major change of the electronic situation around the niobium nucleus. This is further evident by the shift of the ^15^N resonance (as observed by ^1^H-^15^N HMBC NMR experiments) from 444.4 ppm in **[Nb(N**^***t***^**Bu)Py**_**2**_**Cl**_**3**_**]** (Figure S2) to 454.7 ppm in **1-Py** (Figure S8). To set this value also in relation with other niobium imido NHC complexes, complex **I** (Figure 1) previously reported by our group,^74^ shows a ^15^N_imido_ resonance at 466.6 ppm (Figure S62 and Table 1). The high-field shifts of the ^15^N_imido_ resonances in **1-Py** or **1** (*vide infra*) compared to MIC complex **I** (Figure 1) are in line with the MIC ligand being the stronger σ-donor ligand, shifting the ^15^N resonance of the π-accepting imido moiety to lower fields. Unfortunately, for the other imido NHC complexes present in the literature, no ^15^N_imido_ shifts are reported. Due to the low symmetry of the complexes and the resulting broad linewidth, it was not possible to record meaningful and comparable ^93^Nb NMR data of all complexes reported herein. Nevertheless, unambiguous proof for the formation of the desired complex was obtained by X-ray structure analysis of single crystals of **1-Py** grown from a concentrated diethyl ether solution at −40 °C (Figure 2). The niobium center in **1-Py** is hexa-coordinated in a strongly distorted octahedral manner by the three OCO donor atoms of **L**^**1**^, the *tert*-butyl-imido, and the remaining chlorido ligand, as well as an additional equivalent of pyridine as already indicated by ^1^H NMR spectroscopy. The niobium carbene distance Nb1–C1 was found to be 2.260(2) Å, which is slightly shorter (approx. 0.06–0.1 Å) compared to previously reported niobium(V) NHC complexes with monodentate NHC ligands by Marchetti^59−61^ or Wei^88^ and co-workers, similar to previously reported examples of niobium(V) bis-NHC-borate complexes by the Arnold group (2.26–2.27 Å)^69^ but longer than the recent example of a niobium(V) mesoionic carbene complex **I** (Figure 1, 2.196(3) Å).^72^ The niobium imido distance was found to be 1.756(2) Å, which is in the same range as reported for complex **I**(72) and other niobium imido complexes.^36,69^ With a bond distance of 2.509(2) Å, the niobium pyridine distance Nb1–N50 is rather long compared to other niobium(V) pyridine adducts.^89^ It is worth mentioning that the benzimidazol-2-ylidene ligand shows a large pitch angle of 20.3(1)° toward the Nb1–C1 bond axis. Such a large tilt angle has been previously seen for complexes of **L**^**1**^(71,73,74) (*vide infra*). Addition of 1 equiv tris(pentafluorophenyl)borane (Scheme 1) and subsequent recrystallization from *n*-pentane (preformed twice) led to the isolation of the pyridine-free complex **1**. Except for the expected disappearance of the pyridine signals, there is no mentionable difference in the ^1^H NMR spectra of **1-Py***vs***1**. The most notable feature is a high-field shift of the *tert*-butyl imido protons from 0.93 ppm in **1-Py** to 0.86 ppm in **1**. At the same time, the ^15^N resonances observed by ^1^H-^15^N HMBC spectroscopy shift to lower fields from 454.7 to 459.9 ppm in **1-Py***vs***1** (Table 1, Figures S8 and S14). X-ray quality crystals of **1** were obtained from a concentrated *n*-pentane solution at −40 °C within two days. As expected, the complex is now pentacoordinated in a slightly distorted square pyramidal manner (τ_5_ = 0.24). Apart from that, the bond metrics of **1** resemble those found/discussed for **1-Py** (*vide supra* and Tables S1 and S2).

Having complexes **1** and **1-Py** in hand, we were initially interested in its reduction behavior. Since corresponding vanadium *N*-heterocyclic and mesoionic carbene complexes have shown a reversible one-electron reduction wave and the isolation of the corresponding vanadium(IV) complexes was possible,^74^ we were tempted if analogous niobium(IV) complexes could also be accessible. The cyclic voltammograms of **1-Py** in dichloromethane showed only two irreversible oxidations and no reduction processes (Figures S81 and S82), which is in stark contrast to the vanadium systems.^74^ However, switching to THF as a solvent revealed the presence of two irreversible reductions close to the edge of the solvent window at −2.81 and −2.98 V *vs* Fc/[Fc]^+^ couple as an internal standard (Figure S83 and Table S3). Removal of the pyridine co-ligand in **1** leads to a slight shift and a larger separation between the two reductive redox events now appearing at −2.70 and −3.03 V *vs* Fc/[Fc]^+^ (Figure S84 and Table S3). Interestingly, the potential of the first reduction process is slightly lower for the triazol-5-ylidene complex **I** (−2.65 V *vs* Fc/[Fc]^+^; Figures S85 and S86) with respect to the benzimidazol-2-ylidene complexes **1-Py** and **1** (Table S3) while no second reduction process was observed for complex **I**. Given the higher σ-donating properties of MICs *vs* NHCs (*vide supra*),^90−95^ this is in contrast to what would be expected for a metal-centered reduction process.^74^ This indicates that the first reduction might not even be metal-centered, but rather ligand-centered.^96−98^ Despite cyclic voltammetry showing the potential accessibility of low-valent niobium complexes supported by the NHC ligand **L^1^**, the isolation of such a species has yet failed. Various attempts in different solvents (THF, diethyl ether, toluene) to reduce **1-Py** with strong chemical reductants such as potassium metal, potassium graphite, or sodium-naphthalide have failed and resulted in the re-isolation of the starting material **1-Py**. This is particularly surprising given the prevalence of various other niobium(IV)^36,37,41,99^ and niobium(III)^39−41,100^ imido complexes, together with the fact that the first niobium NHC complex was based on niobium(IV),^57^ Also, Marchetti and co-workers have recently isolated bis*-*NHC adducts of niobium(IV)-oxo complexes (Figure 1F).^59^ However, the large stabilization of the niobium(V) oxidation state compared to analogous vanadium(V) complexes^74^ might also be interesting for future separation strategies of these two elements *via* their (redox)-properties.^101−107^ It is worth mentioning at this point that regardless of the solvents (toluene or THF) and synthetic strategies used (**Li**_**2**_**L**^**1**^ or **[H**_**3**_**L**^**1**^**][Cl]**/NEt_3_), the use of NbCl_5_ did not meet with any success to form a heteroleptic Nb(V) chloride complex and no NHC complexes could be isolated or identified from the crude reactions mixtures.

**Scheme 2:**
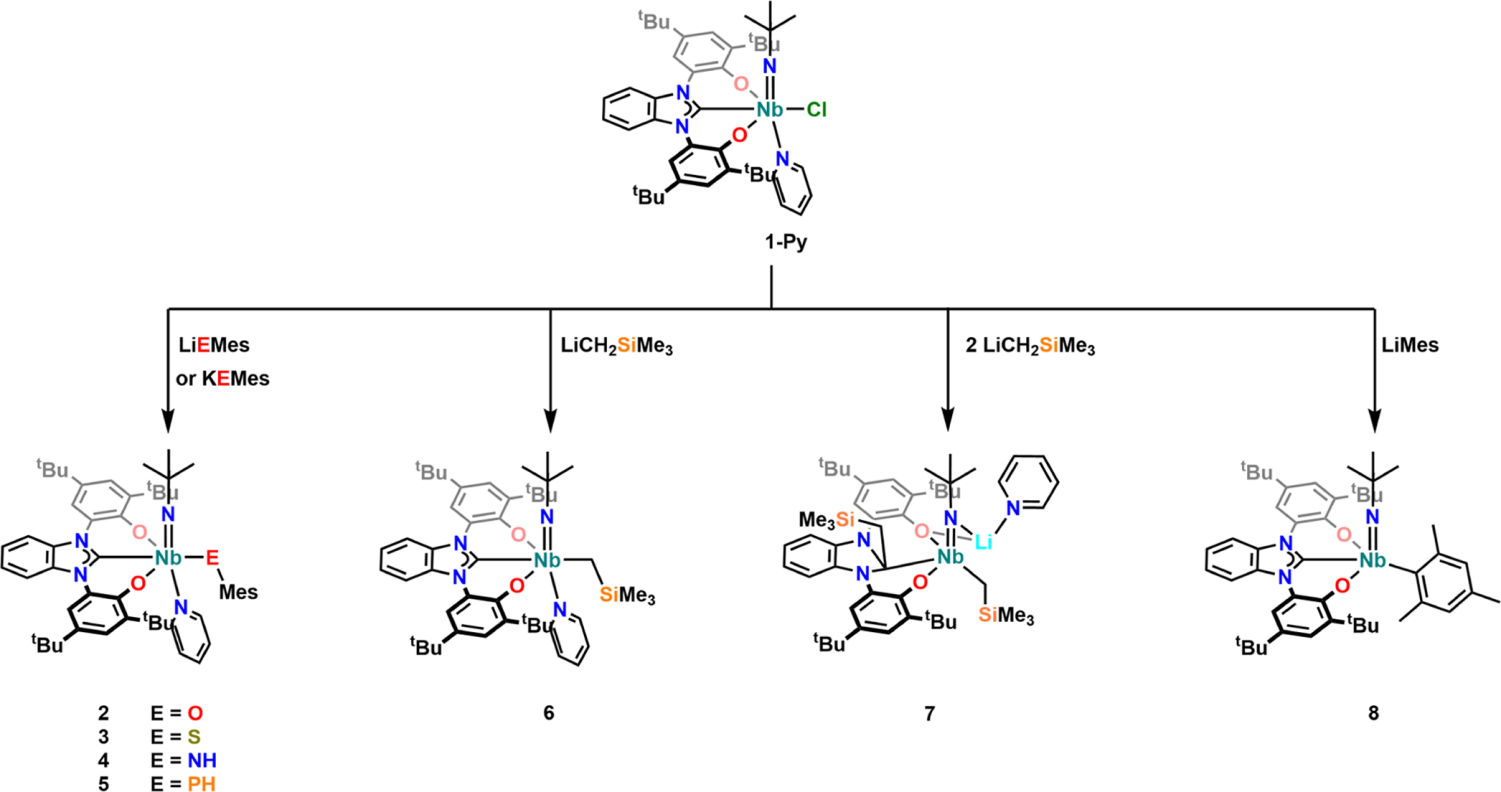
Salt Metathesis Reactions between the Chlorido Complex **1-Py** and a Variety of Different Nucleophiles Leading to Phenolate, Thiophenolate, Amido, Phosphanido, Alkyl, and Aryl Niobium(V) Imido Complexes Supported by the Benzimidazol-2-ylidene Ligand **L**^**1**^

Turning to the chemistry of the niobium(V) complex **1-Py**, we further explored its behavior in salt metathesis reactions (Scheme 2). In this context, we studied an initial series of mesityl-substituted chalcogenide- and pnictogenide-based donor systems, going from mesitolate to thiomesitolate and from primary mesityl amido to primary mesityl phosphanido ligands. The reaction between **1-Py** and the lithium or potassium salts of the desired nucleophiles (LiOMes, KSMes, LiNHMes, and KPHMes, Scheme 2) in diethyl ether results in a smooth conversion to yield the desired complexes in good yields. For all complexes **2**–**5**, successful formation was evident by ^1^H NMR spectroscopy, showing a redistribution of the ligand protons of **L**^**1**^, compared to **1-Py**. The resonance originating from the *tert*-butyl imido ligand is only marginally shifted (0.1 ppm over the whole series) in the ^1^H NMR spectra of the complexes. However, large shifts have been observed in the imido resonances observed by ^1^H-^15^N HMBC NMR experiments, revealing the resonance of the imido nitrogen atom at 434.1, 456.0, 439.1, and 455.6 ppm for **2**–**5** (Figures S20, S26, S32, and S39). In addition to the redistribution of the ligand signals in the ^1^H NMR spectra, the appearance of the characteristic aromatic (6.96, 6.86, 6.83, and 6.88 ppm for **2**–**5**, Figures S15, S21, S27, and S33) and aliphatic (2.57/2.28 ppm in **2**; 2.91/2.17 ppm in **3**, 2.57/2.24 ppm in **4**, and 2.73/2.27 ppm in **5**; Figures S15, S21, S27, and S33) mesityl protons is a further indication for the successful formation of the desired complexes. For the mesityl amido and mesityl phosphanido complexes **4** and **5**, the characteristic E–H protons (E = N, P) were also observed. The resonance of the amido proton in complex **4** was found to appear at 8.69 ppm, showing a ^1^*J*_N–H_ coupling of 65 Hz to the amido nitrogen atom in the ^1^H-^15^N HMBC spectra. A similar ^1^*J*_N–H_ coupling constant was also found in a molybdenum(VI) imido amido complex,^71^ supported by the benzimidazol-2-ylidene ligand **L^1^**. The presence of the amido ligand in **4** was further confirmed by the ^15^N resonance at 188.4 ppm. For the phosphanido complex **5**, the presence of a broad singlet at −43.4 ppm in the respective ^31^P{^1^H} NMR spectrum (Figure S34) and the presence of an IR band at 2340 cm^–1^ (corresponding to the *PH* stretching frequency) are indicative of the successful formation of a primary phosphanido complex.^108^ Note that due to the large quadrupolar moment of the ^93^Nb nucleus, the resonance of the phosphorous atom is significantly broadened and not as sharp as it would be expected for a primary phosphanido complex. This also prevents us from observing or extracting any meaningful ^1^*J*_P–H_ coupling constants from the ^31^P NMR spectrum of the complex (Figure S34). However, the ^1^*J*_P–H_ coupling constant can be extracted from the ^1^H NMR spectrum of **5** (Figure S33), showing the typical *PH* doublet between 5.16 and 4.61 ppm with a ^1^*J*_P–H_ coupling constant of 221 Hz, which is consistent with other mesityl phosphanido complexes throughout the literature.^108−112^ To the best of our knowledge, complex **5** is the first example of an anionic primary phosphanido ligand bound to niobium(V) reported in the literature so far. The only related report we were able to find, was the coordination of a series of neutral primary phosphine complexes with the general formula [NbCpCl_4_(PH_2_R)] (R = Mes, Ph, Cy, Ad, ^*t*^Bu) reported by Hey-Hawkins.^113^ To point out again, all ^1^H NMR spectra of complexes **4**–**7** show the presence of 1 equiv of pyridine being coordinated to the niobium center.

**Table 1:** Overview and Comparison of All ^15^N NMR Shifts Observed by ^1^H-^15^N HMBC Spectroscopy^a^

	**Nb(N^*t*^Bu)Cl_3_Py_2_**	**I**	**1-Py**	**1**	**2**	**3**	**4**	**5**	**6**	**7**	**8**
N_benzimidazole_	n.o.	n.o.^b^	178.8	n.o.	179.2	179.8	179.2	180.5	181.0	108.0	179.9
N_pyridine_	286.2	293.3	289.2	n.o.	296.6	285.4	n.o.	287.6	296.6	n.o.	n.o.
N_imido_	444.4	466.6	454.7	459.9	434.1	456.0	439.1	455.6	445.5	417.3	454.2
N_amido_							188.4				

a n.o. = not observed.

b The triazolylidene nitrogen atoms are observed at 221.1, 258.0, and 334.1 ppm.

**Figure 3:**
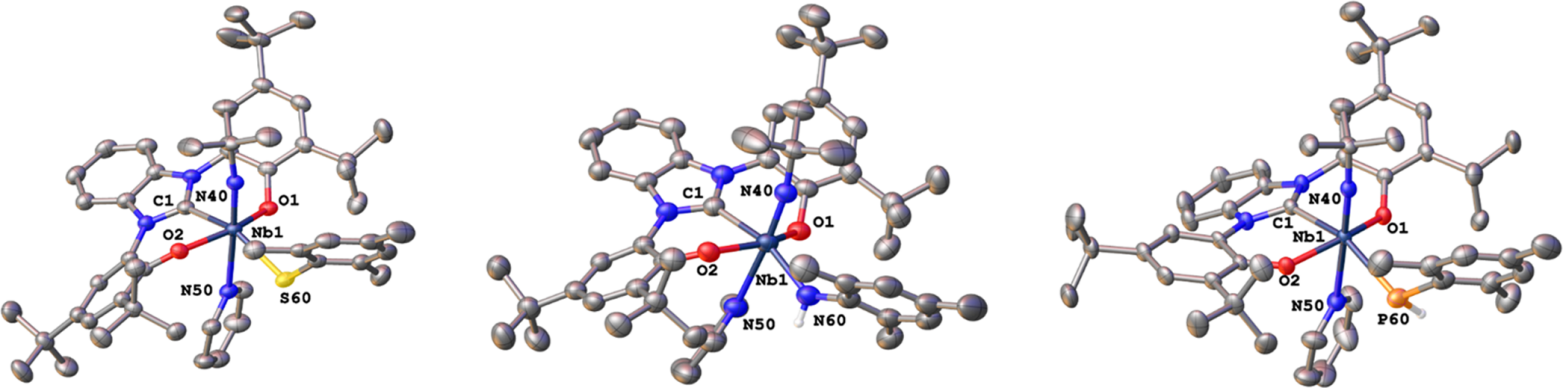
Molecular structures of the thiomesitolate complex **3**, the primary mesityl amido complex **4**, and the primary mesityl phosphanido complex **5**. Hydrogen atoms (except selected ones) and solvent lattice molecules have been omitted for clarity. Ellipsoids are shown at a probability level of 50%.

X-ray-quality single crystals were grown either by slow evaporation of *n*-pentane at room temperature in the case of **3** or from concentrated *n*-pentane solutions at −40 °C in the case of **4** and **5** (Figure 3). Despite numerous attempts, no X-ray-quality crystals of the mesitolate complex **2** could be obtained. The niobium center is hexa-coordinated in a strongly distorted octahedral fashion by the OCO donor atoms, the imido ligand, the anionic co-ligand, and an additional equivalent of pyridine. The Nb1–N50 distances (pyridine nitrogen) are in the range of 2.458(3)–2.498(3) Å and are significantly longer than in previously reported niobium pyridine adducts (0.1–0.15 Å).^89^ The Nb1–C1 bond distances were found to be 2.244(3), 2.254(4), and 2.287(3) Å for **3**, **4**, and **5**, respectively, changing little over the complex series. Similarly, with values of 1.762(3), 1.764(4), and 1.761(3) Å for **3**, **4**, and **5**, the niobium imido distances seem to be rather unaffected by anionic co-ligand in these systems. The Nb-EMes distance is however strongly dependent on the nature of the donor atom E and was found to be 2.4880(8) Å for E being the sulfur in **3**, 2.065(4) Å for E being nitrogen in **4**, and 2.5977(11) Å for E being phosphorous in **5**. For complex **4**, the difference between the imido and the amido group can be seen clearly in the vastly different Nb–N bond distances (1.764(4) Å for Nb1–N40 and 2.065(4) Å for Nb1–N60).

**Figure 4:**
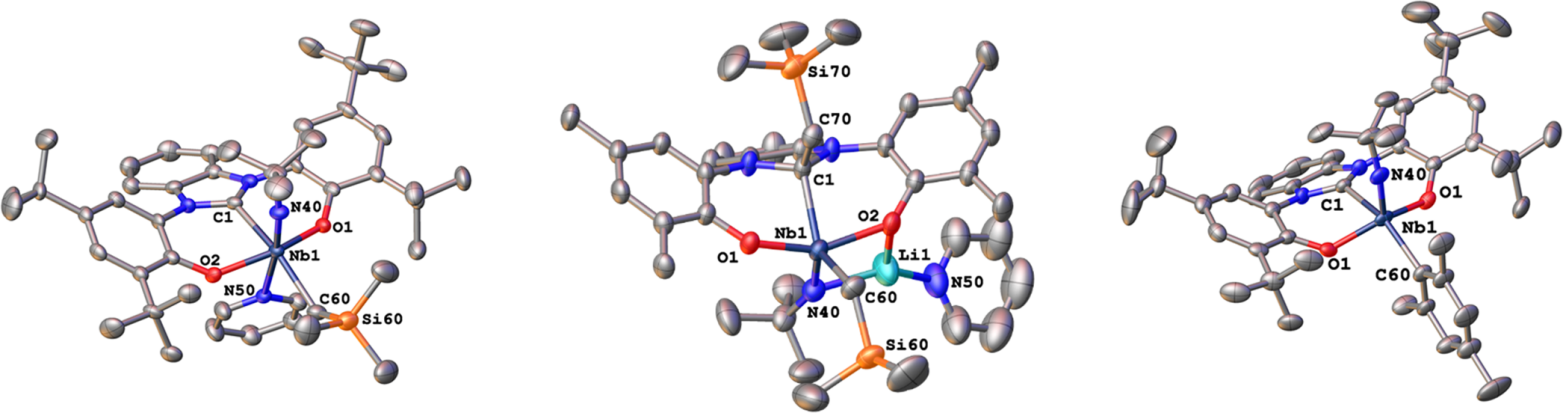
Molecular structures of the neosilyl complex **6**, the anionic neosilyl complex **7**, and the mesityl (aryl) complex **8**. Hydrogen atoms, solvent lattice molecules, have been omitted for clarity. Ellipsoids are shown at a probability level of 50%.

Turning from heteroatom donor ligands to carbon-based donor groups, alkyl as well as aryl complexes are potentially interesting targets. To our delight, mixing 1.1 equiv of neosilyl lithium with **1** in diethyl ether leads to the clean transformation to the desired alkyl complex **6** (Scheme 2). Besides the typical ligand shifts obtained in all salt metathesis reactions, reported in the ^1^H NMR spectrum of the corresponding complexes (*vide supra*), the presence of two additional resonances at 0.82 and 0.61 ppm corresponding to the methylene- and the TMS-protons of the neosilyl ligand indicates the successful formation of the desired mono-alkyl complex **6**. The presence of a silicon atom is further confirmed by a resonance at 0.57 ppm in the ^29^Si NMR spectrum of complex **6**. Albeit the verification of a carbene complex could not be achieved using ^13^C{^1^H} NMR spectroscopy (quadrupolar moment of the ^93^Nb nucleus, *vide supra*), the integrity of the benzimidazol-2-ylidene unit could be proven indirectly *via*^1^H-^15^N HMBC spectroscopy. This revealed a cross-peak at 7.70/180.8 ppm, in which the ^15^N resonance at 180.8 ppm is typical for the benzimidazol-2-ylidene unit and in the comparable range to previous complexes (*vide supra* and Table 1). A cross-peak at 1.11/445.5 ppm in the ^1^H-^15^N HMBC spectrum of **6** (Figure S46) confirms the identity of a *tert*-butyl imido complex. Unambiguous proof for the formation of the desired complex was given by X-ray diffraction analysis performed on single crystals grown by slow evaporation of *n*-pentane. The carbene niobium distance Nb1–C1 was found to be 2.310(4) Å, being the longest carbene niobium distance reported herein. The alkyl carbon C60 was found to be *trans* to the carbene carbon C1 showing an Nb1–C60 distance of 2.230(4) Å and a C1–Nb1–C60 angle of 157.94(15)°. The niobium alkyl bond distance is in the typical range to previously reported niobium(V) alkyl complexes.^69,89^ The clean and direct formation of complex **6** was somewhat surprising since it was shown for related zirconium complexes that in the presence of polar, coordinating agents (THF, PMe_3_), the alkyl group can (reversibly) migrate from the metal center onto the carbene unit, alkylating the latter.^76,114^ However, this reaction might be less favored when going from group IV to group V metals, which can be seen by the similar “innocence” of NHC tantalum alkyl–alkylidene and alkyl–imido complexes in coordinating solvent (*e.g.*, THF) recently reported by Camp and co-workers.^115,116^ To further verify if this “innocence” of the benzimidazolylidene unit is caused by the higher electropositivity of group IV *vs* group V metals, we investigated the reaction between complex **1-Py** and 2 equiv of neosilyl lithium (Scheme 2). This results in the formation of a novel complex **7**, which has two neosilyl groups attached to it. The presence of two neosilyl groups can be confirmed by ^1^H NMR spectroscopy revealing the presence of two distinct singlet signals at 0.61 and −0.04 ppm each integrating to nine protons (Figure S47) and by ^29^Si NMR spectroscopy showing two signals at 1.30 and 0.05 ppm (Figure S50). To further elucidate the connectivity of the two neosilyl groups, we recorded 2D ^1^H-^13^C HSQC and ^1^H-^13^C HMBC spectra of the complex (Figures S52 and S53). This revealed that the proton resonances at 1.05/0.61 ppm as well as 1.27/–0.04 ppm integrating to 2:9 protons each belong to the two different neosilyl groups. We also found that the proton resonance at 1.05 ppm shows a cross-peak at 1.05/37.6 ppm in the ^1^H-^13^C HSQC spectrum. Since the resonance at 37.6 ppm in the ^13^C{^1^H} NMR spectrum of the complex is quite weak and broadened, it can be assumed that this resonance (1.05/37.6 ppm) belongs to a neosilyl ligand directly attached to the niobium center. The broadening of the ^13^C{^1^H} resonance can be traced back to the large quadrupolar moment of niobium. This is in line with the observation of the corresponding TMS resonance at 0.61 ppm, which is similar to the TMS resonance in complex **6**. Moving on to the second neosilyl group, a cross-peak at 1.27/111.4 ppm in the ^1^H-^13^C HMBC spectrum is clearly visible. Furthermore, ^1^H-^15^N HMBC spectroscopy revealed that the methylene protons of the neosilyl group at 1.27 ppm are also coupling to a new nitrogen center at 108.0 ppm. Since the imido nitrogen atom was found at 1.22/417.3 ppm, the only other nitrogen atoms (asides from the pyridine donor, compare Table 1) in the molecule are centered on the (former) benzimidazolylidene framework. The coupling of the methylene protons on the neosilyl group with these benzimidazole nitrogen atoms can only be explained by a nucleophilic attack of a second neosilyl group at the carbene carbon atom. The benzimidazol-2-ylidene ligand is thereby converted into a trianionic benzimidazolide ligand. This is also in line with the large shift of the ^15^N signals arising from the benzimidazolylidene nitrogens, which have been commonly observed between 178.8 and 181.0 ppm^71^ (Table 1), to 108.0 ppm found in **7**. This further suggests that the observed cross-coupling 1.27/111.4 ppm belongs to the former carbene center. In line with the formulation of an anionic complex, in which the dianionic OCO benzimidazolylidene would have been converted into a trianionic species is the presence of a lithium atom resonating at 1.35 ppm in the ^7^Li NMR spectrum of complex **7** (Figure S48). Furthermore, the unusual high-field resonance of the ^15^N imido nitrogen at 417.3 ppm (Figure S54) compared to the one of the alkyl complex **6** at 445.5 ppm (Figure S46) suggests that the lithium counterion might be interacting with the imido nitrogen atom (*vide infra*). Unambiguous proof for the proposed connectivity was given by X-ray diffraction analysis performed on single crystals grown from *n*-pentane at −40 °C within 2 h as green needles (Figure 4, middle). The molecular structure clearly proves the presence of two different neosilyl groups. One is directly attached to the niobium center, while the second one has undergone a nucleophilic attack on the benzimidazolylidene ligand, transforming the dianionic OCO ligand into a trianionic ligand with a carbanionic donor. The niobium carbon distance Nb1–C1 of the new ligand was found to be 2.235(3) Å and is in the same range as the other alkyl (neosilyl) distance Nb1–C60 2.231(3) Å. The transformation of the carbene ligand into a carbanion was furthermore confirmed by the drastic change within the N1–C1 and N2–C1 bond distances going from 1.363(3) and 1.402(3) Å in complex **1-Py** or 1.363(5) and 1.359(5) Å in complex **6** to 1.482(3) and 1.478(3) Å in complex **7**. This elongation of the carbon–nitrogen distance strongly indicates the disruption of the “aromatic” system that formerly stabilized the NHC ligand. The lithium counterion Li1 is coordinated between the imido nitrogen atom N40 and one of the phenolate oxygen donors O2 (as suggested by ^1^H-^15^N HMBC spectroscopy, *vide supra*) and is additionally coordinated by the pyridine donor previously being bound to the niobium center. The coordination of the lithium ion toward N40 and O2 is also reflected in the corresponding distances to the niobium center. With an Nb1–N40 distance of 1.812(3) Å, the imido bond is substantially longer compared to all other niobium imido bonds reported within here and in the literature.^3,48,49,89,100^ Similarly, the Nb1–O2 distance is substantially elongated with 2.0678(19) *vs* 1.9223(19) Å for Nb1–O1. Overall, the niobium ion in complex **7** is five-fold coordinate in a strongly distorted square pyramidal environment (τ_5_ = 0.37). Changing to aryl donors, we found that **1-Py** reacts smoothly with 1 equiv of mesityl lithium^117^ to form the anticipated mesityl complex **8** (Scheme 2). ^1^H NMR spectroscopy revealed five resonances for the mesityl ligand signaling that each mesityl proton is in a different chemical surrounding and integrating in a ratio of 1:1:3:3:3 going from the low-field to high-field signals (Figure S55). The identity of an imido complex is proven by the typical ^15^N resonance at 454.2 ppm (Figure S60) comparable to all other examples reported herein (Table 1). Notably, no sign of pyridine was found in the ^1^H NMR spectrum of **8** suggesting its decoordination upon ligation with the aryl ligand and recrystallization. This was further confirmed by X-ray crystallography on the compound performed on single crystals grown by slow evaporation of a concentrated *n*-pentane solution (Figure 4, right). As already indicated by ^1^H NMR spectroscopy, the pyridine donor is missing. The niobium center is in a distorted square pyramidal coordination environment (τ_5_ = 0.21) coordinated by the OCO-pincer ligand, the imido nitrogen, and the mesityl-carbon. The niobium carbon bond distances were found to be 2.310(9) Å for the Nb1–C1 carbene distance and 2.229(9) Å for the Nb1–C60 carbanion distance. Although **8** is a five-fold coordinate, the Nb1–C1 carbene bond distance is longer than for most of the other (sterically more congested) sixfold coordinated complexes reported within this study (Table S2). Similar to the comparison of complexes **1-Py** and **1**, the decoordination of pyridine does not or only marginally affect the bond metrics between the niobium carbon bond Nb1–C1 and the niobium imido bond Nb1–N40 when comparing the structural parameters of complex **6** with **8** (Tables S1 and S2). The Nb1–C60 carbanion distance is slightly longer compared to other Nb(V) aryl complexes^89^ but almost identical to the niobium alkyl bond in **6**. Notably, “overarylation” similar to complex **7** was not observed, even if an excess of mesityl lithium was used in the reaction.

**Figure 5:**
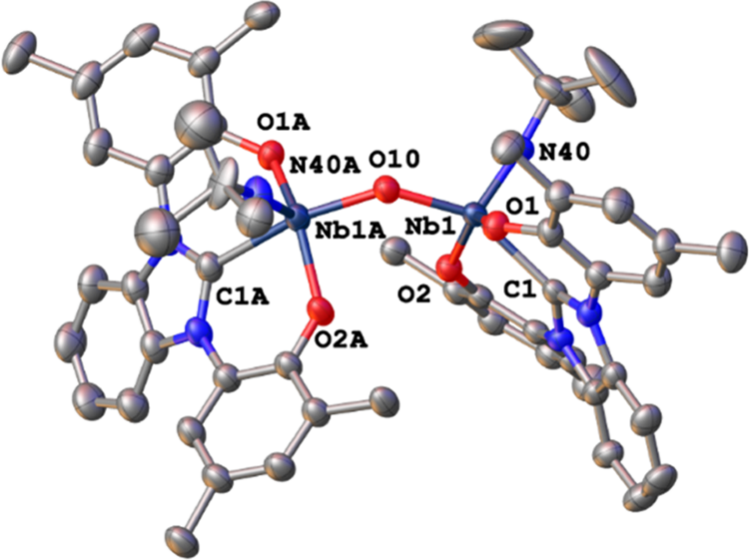
Molecular structure of complex **9**. Hydrogen atoms and ligand *^t^*Bu groups have been omitted for clarity. Ellipsoids are shown at a probability level of 50%.

Finally, we would like to mention that both alkyl and aryl complexes **6** and **8** are of course very prone to undergo hydrolysis, if the solvents used for their synthesis were not rigorously dried and stored over 3 Å molecular sieves. During several crystallization attempts, we observed the formation of the hydrolyzed μ-oxo complex **9**. However, a direct synthesis of complex **9** starting from **1-Py**, **6**, or **8** was not successful up to this point, why no NMR data or other analytical data except for its crystal structure will be reported here. Neither the reaction of complex **1-Py** with silver oxide Ag_2_O nor the reaction with an excess of HMDSO (extruding TMS-Cl) has yet led to the clean isolation of complex **9**. Addition of 1 equiv of water to THF solutions of the complex led to the formation of multiple species from which no useful products could be separated. Therefore, we believe, that **9** is only forming slowly with very small amounts of advantageous and substoichiometric amounts of water being present in the crystallization solvents. Complex **9** (Figure 5) is isostructural to a similar μ-oxo dimolybdenum(V) complex reported by us.^73^ Both niobium centers in **9** are coordinated in a distorted square pyramidal fashion displaying τ_5_ values of 0.19 and 0.27 for Nb1 and Nb1A, respectively. The niobium carbene distances are found to be 2.292(2) and 2.277(2) Å, while the distances to the bridging oxygen atom were found to be 1.9198(15) and 1.9280(15) Å for Nb1–O10 and Nb1A–O10, respectively, and are substantially shorter than the niobium phenolate distances (compare Table S2).

## Conclusions

We have reported the synthesis of 10 new niobium NHC complexes supported by the dianionic bis-phenolate benzimidazolylidene ligand **L^1^** starting from the new niobium benzimidazolylidene complex **1-Py**. Albeit **1-Py** and the pyridine-free complex **1** show irreversible reduction processes in their cyclic voltammogram, the isolation of a reduced niobium(IV) complex is more complicated compared to analogous vanadium complexes.^74^ Among others, these new complexes cover a series of mesityl-substituted chalcogen and pnictogen donor complexes **2**–**5**. Complexes **4** and **5** are potentially interesting precursors for the synthesis of extremely π-loaded^118^ bis-imido and imido-phosphinidene complexes for catalysis and group transfer reactivity.^3,119^ In addition, we reported three alkyl and aryl complexes **6**–**8**. We observed that in the presence of an excess of alkylation reagent (neosilyl lithium in the present case), a nucleophilic attack on the carbene center occurs to yield a new trianionic OCO-pincer ligand in complex **7**. Since a similar kind of reactivity was reported to be reversible in the literature,^76^ this could be an interesting feature in group transfer reactivity and catalysis using the NHC ligand as a chemically noninnocent ligand. Overall, it can be assumed that the complexes reported here will be interesting and intriguing reagents to further explore the reactivity and utility of NHC-based niobium imido complexes.

## Experimental Section

### General Remarks

If not stated otherwise, all transformations were conducted in an argon-filled glovebox under inert conditions. Solvents were dried by an MBraun SPS system and stored over activated molecular sieves (3 Å) for at least 1 day prior to use. C_6_D_6_ was dried over sodium/benzophenone followed by vacuum transfer and three freeze–pump–thaw cycles. The proligand **[H**_**3**_**L**^**1**^**][Cl]**(75,76) mesityl lithium^117^ and the niobium precursor [Nb(N*^t^*Bu)Py_2_Cl_3_]^124^ were synthesized according to the literature. LiOMes and LiNHMes were obtained by deprotonating the corresponding phenol or aniline in pentane using 1.2 equiv of *n*-BuLi and filtering off the white products. In a similar way, KSMes and KPHMes were obtained by deprotonating the corresponding thiophenol and primary phosphine using KHMDS in toluene.^108^ LiCH_2_TMS was purchased as a solution in pentane, and the solvent was evaporated under high vacuum at room temperature to give a very pyrophoric off-white solid. NMR spectra were collected at ambient temperature on a Bruker AV-300 (MHz), Ascent 400 (400 MHz). ^1^H and ^13^C{^1^H} NMR chemical shifts (δ) are reported in ppm and were calibrated to residual solvent peaks. ^7^Li, ^15^N, ^29^Si, and ^31^P NMR spectra are calibrated *vs* LiCl, NH_3_, SiMe_4_, and H_3_PO_4_, respectively, as an external standard. Structural assignments were made with additional information from gCOSY, gHSQC, and gHMBC experiments. IR spectra were collected using a Bruker Alpha IR spectrometer with an ATR measurement setup used inside an argon-filled glovebox with approx. 0.5 mg of sample. Cyclic voltammetry was recorded using a BioLogic potentiostat and a three-electrode array (working electrode: glassy carbon, counter electrode: platinum, reference electrode: silver). Solvents for cyclic voltammetry (THF and DCM, electrochemical grade) have been dried over activated molecular sieves for at least 48 h prior to use. The supporting electrolyte NBu_4_PF_6_ was dried under high vacuum (1 × 10^–3^ mbar) at 60 °C for 24 h and was stored in an argon-filled glovebox. All electrochemical experiments have been performed in 0.1 M solutions of NBu_4_PF_6_ in the corresponding solvent at 0.001 M solutions of the analyte under strictly inert conditions in an argon-filled glovebox.

### Synthetic Procedures

#### [Nb(V)L^1^(N^*t*^Bu)(Py)Cl] (**1-Py**)

In a 100 mL J. Young Schlenk flask, proligand **[H**_**3**_**L**^**1**^**][Cl]** (1.69 g, 1.0 equiv, 3.00 mmol) was dissolved in THF (20 mL). A solution of lithium diisopropylamide (1.06 g, 3.3 equiv, 9.90 mmol) in THF (5 mL) was added dropwise at ambient temperature. The mixture turned bright green during the addition. After 30 min at room temperature, a solution of [Nb(V)(N^*t*^Bu)Py_2_Cl_3_] (1.29 g, 1.0 equiv, 3.00 mmol) in THF (5 mL) was added. After 15 h at ambient temperature, the solvent was removed *in vacuo*. The residue was suspended in diethyl ether, centrifuged, and filtered through a pipette equipped with a glass fiber filter to remove lithium chloride. The filtrate was concentrated to approx. 5 mL of volume and stored at −40 °C. Large colorless blocks were obtained within 12 h. Crystals were separated, washed with *n*-pentane, and dried *in vacuo*. The supernatant was concentrated and stored at −40 °C for a second batch of faint-yellow to colorless crystals. Yield: 65% (1.56 g, 1.95 mmol). ^1^H NMR (400 MHz, benzene-*d*_6_) δ 8.74 (m, 2H, Py-*H*), 7.85 (m, 2H, aryl-*H*), 7.75 (d, *J* = 2.2 Hz, 2H, aryl-*H*), 7.63 (d, *J* = 2.3 Hz, 2H, aryl-*H*), 6.92 (m, 2H, aryl-*H*), 6.48 (m, 1H, Py-*H*), 6.25 (m, 2H, Py-*H*), 1.91 (s, 18H, C(C*H*_3_)_3_), 1.34 (s, 18H, C(C*H*_3_)_3_), 0.93 (s, 9H, Nb=N(C*H*_3_)_3_). ^13^C{^1^H} NMR (101 MHz, benzene-*d*_6_) δ 154.8 (aryl-*C*), 149.5 (Py-*C*H), 140.2 (aryl-*C*), 140.0 (aryl-*C*), 136.7 (Py-*C*H), 134.2 (aryl-*C*), 126.3 (aryl-*C*), 124.4 (aryl-*C*H), 123.6 (Py-*C*H), 122.8 (aryl-*C*H), 116.3 (aryl-*C*H), 114.1 (aryl-*C*H), 68.2 (Nb=N*C*(CH_3_)_3_), 36.4 (*C*(CH_3_)_3_), 34.7 (*C*(CH_3_)_3_), 31.8 (C(*C*H_3_)_3_), 31.4 (Nb=NC(*C*H_3_)_3_), 30.7 (C(*C*H_3_)_3_). ^15^N NMR (71 MHz, benzene-*d*_6_) δ 454.7 (Nb=*N*^*t*^Bu), 289.2 (*N*_Py_), 178.8 (*N*_benz_). Elemental analysis calcd for C_44_H_58_N_4_O_2_Cl_1_Nb_1_ C 65.79 H 7.28 N 6.97 found C 66.12 H 7.35 N 6.81.

#### [Nb(V)L^1^(N^*t*^Bu)Cl] (**1**)

In a 20 mL scintillation vial, complex **1** (80 mg, 1.0 equiv, 0.100 mmol) was dissolved in benzene (2 mL). A solution of tris(perfluorophenyl) borane (51 mg, 1.0 equiv, 0.100 mmol) in benzene (2 mL) was added dropwise at room temperature. After 15 h, the faint green solution was filtered through a pipette equipped with a glass fiber filter and solvents were removed *in vacuo*. The residue was redissolved in *n*-pentane, filtered again, concentrated to approx. 1–2 mL, and stored at −40 °C for crystallization. Faint-yellow crystals were obtained after two recrystallization cycles. Yield: 48% (35 mg, 0.048 mmol). ^1^H NMR (400 MHz, benzene-*d*_6_) δ 7.98 (m, 2H, aryl-*H*), 7.78 (d, *J* = 2.4 Hz, 2H, aryl-*H*), 7.62 (d, *J* = 2.4 Hz, 2H, aryl-*H*), 7.01 (m, 2H, aryl-*H*), 1.87 (s, 18H, C(C*H*_3_)_3_), 1.35 (s, 18H, C(C*H*_3_)_3_), 0.86 (s, 9H, Nb=N(C*H*_3_)_3_). ^13^C{^1^H} NMR (101 MHz, benzene-*d*_6_) δ 154.2 (aryl-*C*), 140.3 (aryl-*C*), 134.3 (aryl-*C*), 126.1 (aryl-*C*), 124.7 (aryl-*C*H), 122.8 (aryl-*C*H), 116.3 (aryl-*C*H), 114.1 (aryl-*C*H), 69.3 (Nb=N*C*(CH_3_)_3_), 36.3 (*C*(CH_3_)_3_), 34.7 (*C*(CH_3_)_3_), 31.8 (C(*C*H_3_)_3_), 31.4 (Nb=NC(*C*H_3_)_3_), 30.6 (C(*C*H_3_)_3_). ^15^N NMR (71 MHz, benzene-*d*_6_) δ 460.9 (Nb=*N*^*t*^Bu). Elemental analysis calcd for C_39_H_53_N_3_O_2_Cl_1_Nb_1_ C 64.68 H 7.38 N 5.80 found C 65.32 H 7.24 N 6.10. Note: the large deviation in the carbon values most likely arises from metal carbide formation, a typical problem in performing EA’s in early transition metal chemistry.

### General Procedure for the Salt Metathesis Reactions

In a 20 mL scintillation vial, niobium complex **1-Py** was dissolved in 5 mL of diethyl ether and cooled to −40 °C. In a separate vial, the corresponding lithium/potassium salt (1.0–1.5 equiv) was suspended or dissolved in diethyl ether and cooled to −40 °C as well. The resulting solution or suspension was then added slowly into the solution of the parent niobium complex. After the addition was complete, the cooling block was removed, leaving the mixture to stir at ambient temperature overnight. The slightly colored (faint orange to light brown if not stated otherwise) suspension was filtered through a pipette equipped with a glass fiber filter, and the solution was evaporated to dryness. The crude residue was dissolved in *n*-pentane (5–10 mL), filtered, and concentrated to approx. 1–2 mL of volume. Crystalline material was obtained overnight at −40 °C. The supernatant was discarded, and the crystals were washed with cold *n*-pentane and dried *in vacuo*. If necessary, the recrystallization process was repeated to afford the clean product.

#### [Nb(V)L^1^(N^*t*^Bu)(Py)(OMes)] (**2**)

From complex **1-Py** (161 mg, 1.0 equiv, 0.200 mmol) and lithium mesitolate (28 mg, 1.0 equiv, 0.200 mmol). Faint-yellow powder. Yield: 57% (105 mg, 0.114 mmol). ^1^H NMR (400 MHz, benzene-*d*_6_) δ 8.56 (m, 2H Py-*H*), 7.86 (m, 2H, aryl-*H*), 7.75 (d, *J* = 2.3 Hz, 2H, aryl-*H*), 7.59 (d, *J* = 2.3 Hz, 2H, aryl-*H*), 6.96 (s, 2H, Mes-*H*), 6.93 (m, 2H, aryl-*H*), 6.63 (m, 1H, Py-*H*), 6.34 (m, 2H, Py-*H*), 2.57 (s, 6H, C*H*_3_), 2.28 (s, 3H, C*H*_3_), 1.76 (s, 18H, C(C*H*_3_)_3_), 1.34 (s, 18H, C(C*H*_3_)_3_), 0.93 (s, 9H, Nb=N(C*H*_3_)_3_). ^13^C{^1^H} NMR (101 MHz, benzene-*d*_6_) δ 159.9 (aryl-*C*), 155.2 (aryl-*C*), 149.6 (Py-*C*H), 140.3 (aryl-*C*), 139.7 (aryl-*C*), 136.2 (Py-*C*H), 134.3 (aryl-*C*), 129.1 (Mes-*C*H), 127.5 (Mes-*C*), 127.0 (aryl-*C*), 126.8 (aryl-*C*), 124.2 (Py-*C*H), 123.5 (Py-*C*H), 122.5 (aryl-*C*H), 116.5 (aryl-*C*H), 114.0 (aryl-*C*H), 68.1 (Nb=NC(*C*H_3_)_3_), 36.3 ((C(*C*H_3_)_3_)), 34.6 (*C*(CH_3_)_3_), 32.2 (Nb=N*C*(CH_3_)_3_), 31.8 (C(*C*H_3_)_3_), 30.5 (C(*C*H_3_)_3_), 21.0 (*C*H_3_), 18.3 (*C*H_3_). ^15^N NMR (41 MHz, benzene-*d*_6_) δ 434.1 (Nb=*N*^*t*^Bu), 296.6 (*N*_Py_), 179.2 (*N*_benz_). Elemental analysis calcd for C_53_H_69_N_4_O_3_Nb_1_ C 70.49 H 7.70 N 6.20 found C 70.25 H 7.35 N 6.27.

#### [Nb(V)L^1^(N^*t*^Bu)(Py)(SMes)] (**3**)

From complex **1-Py** (80 mg, 1.0 equiv, 0.100 mmol) and potassium thiomesitolate (19 mg, 1.0 equiv, 0.100 mmol). Faint-yellow crystals. Yield: 51% (48 mg, 0.051 mmol). ^1^H NMR (400 MHz, benzene-*d*_6_) δ 8.51 (m, 2H, Py-*H*), 7.80 (m, 2H, aryl-*H*), 7.66 (d, *J* = 1.8 Hz, 2H, aryl-*H*), 7.63 (d, 2H, aryl-*H*), 6.89 (m, 2H, aryl-*H*), 6.86 (s, 2H, Mes-*H*), 6.45 (m, 1H, Py-*H*), 6.15 (m, 2H, Py-*H*), 2.91 (s, 6H, C*H*_3_), 2.17 (s, 3H, C*H*_3_), 1.89 (s, 18H, C(C*H*_3_)_3_), 1.33 (s, 18H, C(C*H*_3_)_3_), 0.94 (s, 9H, Nb=N(C*H*_3_)_3_). ^13^C{^1^H} NMR (101 MHz, benzene-*d*_6_) δ 155.0 (aryl-*C*), 149.6 (Py-*C*H), 140.9 (aryl-*C*), 140.4 (aryl-*C*), 139.6 (aryl-*C*), 139.5 (aryl-*C*), 136.4 (Py-*C*H), 134.2 (aryl-*C*), 132.9 (Mes-*C*), 126.3 (aryl-*C*), 124.3 (aryl-*C*H), 123.2 (Py-*C*H), 122.7 (aryl-*C*H), 116.5 (aryl-*C*H), 114.0 (aryl-*C*H), 69.1 (Nb=N*C*(CH_3_)_3_), 36.4 (*C*(CH_3_)_3_), 34.6 (*C*(CH_3_)_3_), 31.8 (C(*C*H_3_)_3_), 31.5 (Nb=NC(*C*H_3_)_3_), 31.0 (C(*C*H_3_)_3_), 25.7 (*C*H_3_), 25.6 (*C*H_3_), 21.0 (*C*H_3_). ^15^N NMR (71 MHz, benzene-*d*_6_) δ 456.0 (Nb=*N*^*t*^Bu), 285.4 (*N*_Py_), 179.8 (*N*_benz_). Elemental analysis calcd for C_53_H_69_N_4_O_2_S_1_Nb_1_·2C_4_H_10_O_1_ C 68.64 H 8.40 N 5.25 found C 68.52 H 8.09 N 5.33.

#### [Nb(V)L^1^(N^*t*^Bu)(Py)(NHMes)] (**4**)

From complex **1-Py** (161 mg, 1.0 equiv, 0.200 mmol) and lithium mesitylamide (28 mg, 1.0 equiv, 0.200 mmol). Faint orange crystals. Yield: 50% (92 mg, 0.050 mmol). ^1^H NMR (400 MHz, benzene-*d*_6_) δ 8.69 (s, 1H, N*H*), 8.57 (m, 2H, Py-*H*), 7.80 (m, 2H, aryl-*H*), 7.76 (d, *J* = 2.4 Hz, 2H, aryl-*H*), 7.59 (d, *J* = 2.4 Hz, 2H, aryl-*H*), 6.93 (m, 2H, aryl-*H*), 6.86 (m, 1H, Py-*H*), 6.83 (s, 2H, Mes-*H*), 6.56 (m, 2H, Py-*H*), 2.57 (s, 6H, C*H*_3_), 2.24 (s, 3H, C*H*_3_), 1.70 (s, 18H, C(C*H*_3_)_3_), 1.36 (s, 18H, C(C*H*_3_)_3_), 0.96 (s, 9H, Nb=N(C*H*_3_)_3_). ^13^C{^1^H} NMR (101 MHz, benzene-*d*_6_) δ 154.5 (aryl-*C*), 150.1 (Py-*C*H), 149.6 (Mes-*C*), 140.5 (aryl-*C*), 140.0 (aryl-*C*), 135.5 (Py-*C*H), 134.4 (aryl-*C*), 129.4 (Mes-*C*H), 127.6 (aryl-*C*), 126.8 (aryl-*C*), 124.4 (aryl-*C*HHH), 124.2, 123.5 (Py-*C*H), 122.5 (aryl-*C*HHH), 116.7 (aryl-*C*HHH), 114.0 (aryl-*C*HHH), 69.6 (Nb=N*C*(CH_3_)_3_), 36.2 (*C*(CH_3_)_3_), 34.7 (*C*(CH_3_)_3_), 32.0 (Nb=NC(*C*H_3_)_3_), 31.9 (C(*C*H_3_)_3_), 30.3 (C(*C*H_3_)_3_), 20.9 (*C*H_3_), 19.9 (*C*H_3_). ^15^N NMR (41 MHz, benzene-*d*_6_) δ 439.0 (Nb=*N*^*t*^Bu), 188.4 (*N*HMes), 179.4 (*N*_benz_). Elemental analysis calcd for C_53_H_70_N_5_O_2_Nb_1_ C 70.57 H 7.82 N 7.76 found C 70.77 H 7.60 N 7.65.

#### [Nb(V)L^1^(N^*t*^Bu)(Py)(PHMes)] (**5**)

From complex **1-Py** (331 mmol, 1.0 equiv, 0.400 mmol) and potassium mesitylphosphanide (114 mg, 1.5 equiv, 0.600 mmol), resulting in a blood red solution. Bright orange crystals. Yield: 74% (275 mg, 0.300 mmol). ^1^H NMR (400 MHz, benzene-*d*_6_) δ 8.63 (m, 2H, Py-*H*), 7.80 (m, 2H, aryl-*H*), 7.67 (d, *J* = 2.4 Hz, 2H, aryl-*H*), 7.63 (d, *J* = 2.4 Hz, 2H, aryl-*H*), 6.88 (s, 2H, Mes-*H*), 6.86 (m, 2H, aryl-*H*), 6.48 (m, 1H, Py-*H*), 6.21 (m, 2H, Py-*H*), 4.88 (d, *J* = 220.6 Hz, 1H, P*H*), 2.73 (s, 6H, C*H*_3_), 2.27 (s, 3H, C*H*_3_), 1.87 (s, 18H, C(C*H*_3_)_3_), 1.33 (s, 18H, C(C*H*_3_)_3_), 1.02 (s, 9H, Nb=N(C*H*_3_)_3_). ^13^C{^1^H} NMR (101 MHz, benzene-*d*_6_) δ 154.6 (aryl-*C*), 149.7 (Py-*C*H), 140.5 (Mes-*C*), 139.7 (aryl-*C*), 138.5 (Mes-*C*), 136.5 (Py-*C*H), 134.4 (aryl-*C*), 131.9 (Mes-*C*), 128.3 (Mes-*C*H),126.0 (aryl-*C*), 124.2 (aryl-*C*H), 123.6 (Py-*C*H), 122.7 (aryl-*C*H), 116.7 (aryl-*C*H), 113.8 (aryl-*C*H), 68.6 (Nb=N*C*(CH_3_)_3_), 36.4 (*C*(CH_3_)_3_), 34.6 (*C*(CH_3_)_3_), 32.1 (Nb=NC(*C*H_3_)_3_), 31.8 (C(*C*H_3_)_3_), 31.0 (C(*C*H_3_)_3_), 25.7 (*C*H_3_), 21.1 (*C*H_3_). ^31^P NMR (162 MHz, benzene-*d*_6_) δ −43.38 (br). ^15^N NMR (41 MHz, benzene-*d*_6_) δ 455.6 (Nb=N^*t*^Bu), 287.6 (*N*_Py_), 180.5 (*N*_benz_). IR (ATR, cm^–1^) 2340 (P*H*) Elemental analysis calcd for C_53_H_70_N_4_O_2_P_1_Nb_1_·KCl C 64.07 H 7.10 N 5.64 found C 64.17 H 7.38 N 5.65.

#### [Nb(V)L^1^(N^*t*^Bu)(Py)(CH_2_TMS)] (**6**)

From complex **1-Py** (82 mg, 1.0 equiv, 0.102 mmol) and (trimethylsilyl)methyllithium (11 mg, 1.1 equiv, 0.117 mmol). Faint-yellow blocks. Yield: 61% (52 mg, 0.061 mmol). ^1^H NMR (400 MHz, benzene-*d*_6_) δ 8.58 (m, 2H, Py-*H*), 7.81 (m, 2H, aryl-*H*), 7.70 (d, *J* = 2.5 Hz, 2H, aryl-*H*), 7.65 (d, *J* = 2.4 Hz, 2H, aryl-*H*), 6.88 (m, 2H, aryl-*H*), 6.46 (m, 1H, Py-*H*), 6.19 (m, 2H, Py-*H*), 1.90 (s, 18H, C(C*H*_3_)_3_), 1.34 (s, 18H, C(C*H*_3_)_3_), 1.11 (s, 9H, Nb=N(C*H*_3_)_3_), 0.82 (s, 2H, CH_2_), 0.61 (s, 9H). ^13^C{^1^H} NMR (101 MHz, benzene-*d*_6_) δ 154.2 (aryl-*C*), 149.4 (Py-*C*H), 140.3 (aryl-*C*), 139.6 (aryl-*C*), 135.9 (Py*-C*H), 134.6 (aryl-*C*), 126.0 (aryl-*C*), 123.9 (aryl-*C*H), 123.5 (Py-*C*H), 122.7 (aryl-*C*H), 117.2 (aryl-*C*H), 67.8 (Nb=N*C*(CH_3_)_3_), 42.1 (C*H*_2_TMS), 36.5 (*C*(CH_3_)_3_), 34.6 (*C*(CH_3_)_3_), 33.0 (Nb=NC(*C*H_3_)_3_), 31.8 (C(*C*H_3_)_3_), 31.1 (C(*C*H_3_)_3_), 4.2 (TMS). ^29^Si NMR (80 MHz, benzene-*d*_6_) δ 0.57. ^15^N NMR (71 MHz, benzene-*d*_6_) δ 445.5 (Nb=*N*^*t*^Bu), 296.6 (*N*_Py_), 180.9 (*N*_benz_). Elemental analysis calcd for C_48_H_69_N_4_O_2_Si_1_Nb_1_ C 67.42 H 8.13 N 6.55 found C 67.02 H 7.77 N 6.23.

#### [Nb(V)L^neo-silyl^(N^*t*^Bu)(CH_2_TMS)]Li·Py (**7**)

From complex **1-Py** (80 mg, 1.0 equiv, 0.100 mmol) and (trimethylsilyl)methyllithium (23 mg, 2.4 equiv, 0.120 mmol), resulting in a deep green solution. Dark green needles. Yield: 42% (40 mg, 0.042 mmol). ^1^H NMR (400 MHz, benzene-*d*_6_) δ 7.62 (d, *J* = 2.4 Hz, 2H, aryl-*H*), 7.53 (m, 2H, Py-*H*), 7.12 (d, *J* = 2.5 Hz, 2H, aryl-*H*), 6.97 (m, 2H, aryl-*H*), 6.72 (m, 1H, Py-H), 6.68 (m, 2H, aryl-*H*), 6.42 (m, 2H, Py-*H*), 1.57 (s, 18H, C(C*H*_3_)_3_), 1.35 (s, 18H, C(C*H*_3_)_3_), 1.27 (s, 2H, C*H*_2_Si(CH_3_)_3_), 1.23 (s, 9H, Nb=N(C*H*_3_)_3_), 1.06 (s, 2H, C*H*_2_Si(CH_3_)_3_), 0.61 (s, 9H, CH_2_Si(C*H*_3_)_3_), −0.04 (s, 9H, CH_2_Si(C*H*_3_)_3_). ^13^C{^1^H} NMR (101 MHz, benzene-*d*_6_) δ 149.9 (aryl-*C*), 148.9 (Py-*C*H), 141.9 (aryl-*C*), 139.0 (aryl-*C*), 138.3 (aryl-*C*), 137.8 (Py-*C*H), 130.6 (aryl-*C*), 124.4 (Py-*C*H), 118.8 (aryl-*C*H), 117.9 (aryl-*C*H), 117.4 (aryl-*C*H), 111.5 (carbanion), 103.5 (aryl-*C*H), 67.6 (Nb=N*C*(CH_3_)_3_), 37.6 (Nb-*C*H_2_TMS), 35.7 (*C*(CH_3_)_3_), 34.8 (*C*(CH_3_)_3_), 34.6 (Nb=NC(*C*H_3_)_3_), 31.8 (C(*C*H_3_)_3_), 30.5 (C(*C*H_3_)_3_), 15.1 (*C*H_2_TMS), 4.2 (Nb-CH_2_*TMS*), 0.7 (CH_2_*TMS*). ^7^Li NMR (156 MHz, benzene-*d*_6_) δ 1.35. ^29^Si NMR (80 MHz, benzene-*d*_6_) δ 1.30, 0.05. ^15^N NMR (41 MHz, benzene-*d*_6_) δ 417.3 (Nb=*N*^*t*^Bu), 108.0. Elemental analysis calcd for C_52_H_80_N_4_O_2_Si_1_Li_1_Nb_1_ C 67.80 H 8.75 N 6.08 found C 67.51 H 8.34 N 5.81.

#### [Nb(V)L^1^(N^*t*^Bu)(Py)(Mes)] (**8**)

From complex **1-Py** (80 mg, 1.0 equiv, 0.100 mmol) and mesityl lithium (14 mg, 1.1 equiv, 0.110 mmol). Colorless crystals. Yield: 48% (39 mg, 0.048 mmol). ^1^H NMR (400 MHz, benzene-*d*_6_) δ 7.81 (m, aryl-*H*), 7.78 (d, *J* = 2.4 Hz, 2H, aryl-*H*), 7.58 (d, *J* = 2.3 Hz, 2H, aryl-*H*), 7.19 (s, 1H, Mes-*H*), 6.95 (m, 2H, aryl-*H*), 6.72 (s, 1H, Mes-*H*), 3.36 (s, 3H, C*H*_3_), 2.63 (s, 3H, C*H*_3_), 2.24 (s, 3H, C*H*_3_), 1.58 (s, 18H, C(C*H*_3_)_3_), 1.35 (s, 18H, C(C*H*_3_)_3_), 1.02 (s, 9H, Nb=N(C*H*_3_)_3_). ^13^C{^1^H} NMR (101 MHz, benzene-*d*_6_) δ 184. 7 (Mes-*C*), 153.9 (aryl-*C*), 143.4 (Mes-*C*), 141.9 (Mes-*C*), 140.8 (aryl-*C*), 140.5 (aryl), 138.1 (Mes-*C*), 134.3 (aryl-*C*), 126.9 (Mes-*C*H), 126.6 (Mes-*C*H), 124.7 (aryl-*C*H), 122.6 (aryl-*C*H), 116.6 (aryl-*C*H), 114.1 (aryl-*C*H), 70.5 (Nb=N*C*(CH_3_)_3_), 36.1 (*C*(CH_3_)_3_), 34.7 (*C*(CH_3_)_3_), 32.9 (Nb=NC(*C*H_3_)_3_), 31.8 (C(*C*H_3_)_3_), 30.2 (*C*H_3_), 30.2 (C(*C*H_3_)_3_), 21.8 (*C*H_3_), 21.3 (*C*H_3_). ^15^N NMR (71 MHz, benzene-*d*_6_) δ 454.2 (Nb=*N*^*t*^Bu), 179.9 (*N*_benz_). Elemental analysis calcd for C_48_H_64_N_3_O_2_Nb_1_ C 71.36 H 7.98 N 5.20 found C 71.65 H 8.24 N 5.15.

### X-ray Crystallography

X-ray diffraction experiments were performed at the analytical facility of the University of Paderborn or at the University of Innsbruck. Data collection was performed using the ApexIII and ApexIV software package on a Bruker D8 Venture (Paderborn) or on a Bruker D8 Quest instrument (Innsbruck). Data refinement and reduction were performed using the Bruker ApexIII 2019 or ApexIV suite 2022. Using the OLEX2 software package,^120^ all structures were solved with SHELXT^121^ and refined with SHELXL.^122^ Strongly disordered solvent molecules have been removed using the SQUEEZE operation.^123^ All nonhydrogen atoms were refined anisotropically, and hydrogen atoms were included at the geometrically calculated positions and refined using a riding model. For further crystallographic details, see Tables S1 and S2 in the Supporting Information.

## Supporting Information Available

NMR spectra; IR spectra; crystallographic details; and cyclic voltammograms (PDF)
