# Supplementary material for: Reactivity and Structure of a Bis-phenolate Niobium NHC Complex
Source: ACS Org Inorg Au. 2022 Dec 5;3(1):59–71. doi: 10.1021/acsorginorgau.2c00028 (PMC9896488; doi:10.1021/acsorginorgau.2c00028)
Supplement: Supplementary file 1 — gg2c00028_si_001.pdf [file gg2c00028_si_001.pdf]

# Reactivity and Structure of a bis-Phenolate Niobium NHC Complex

Florian R. Neururer,[a] Konstantin Huter,[a] Michael Seidl,[a] and Stephan Hohloch*[a]

[a] University of Innsbruck, Faculty of Chemistry, Institute of Inorganic, General and Theoretical Chemistry, Innrain 80-82, 6020 Innsbruck, Austria. E-Mail: Stephan.Hohloch@uibk.ac.at

Supporting Information

## Table of contents

1. NMR spectra ........ 2
2. IR spectroscopy ........ 64
3. Crystallographic details ........ 72
4. Electrochemistry (Cyclic Voltammetry) ........ 84

# 1. NMR spectra

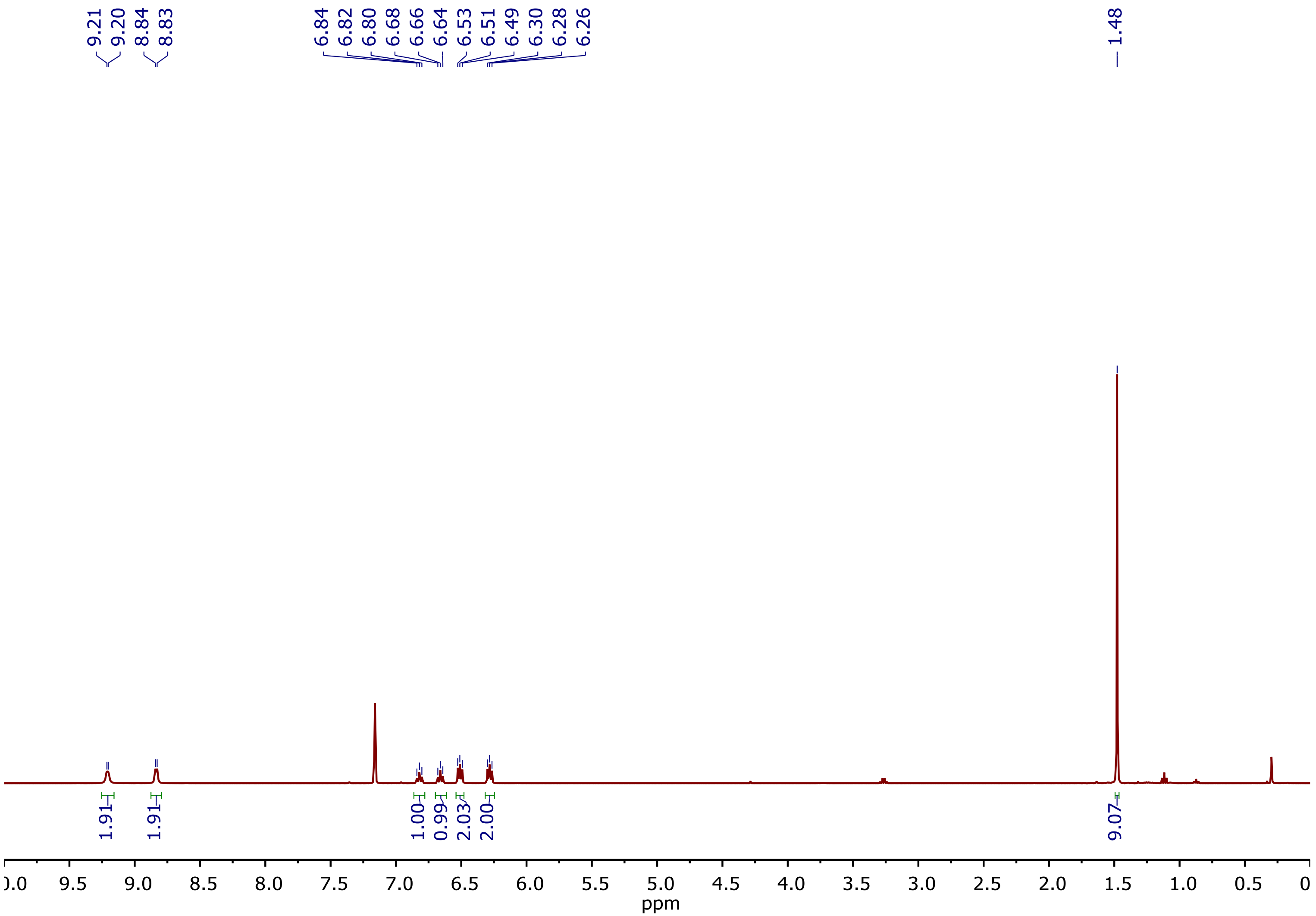


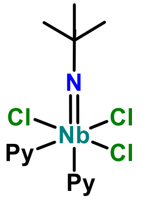


Figure S 1: $^{1}$H NMR of **Nb(N$^{t}$Bu)Cl$_3$Py$_2$** in $C_6D_6$ at 298 K (400 MHz)

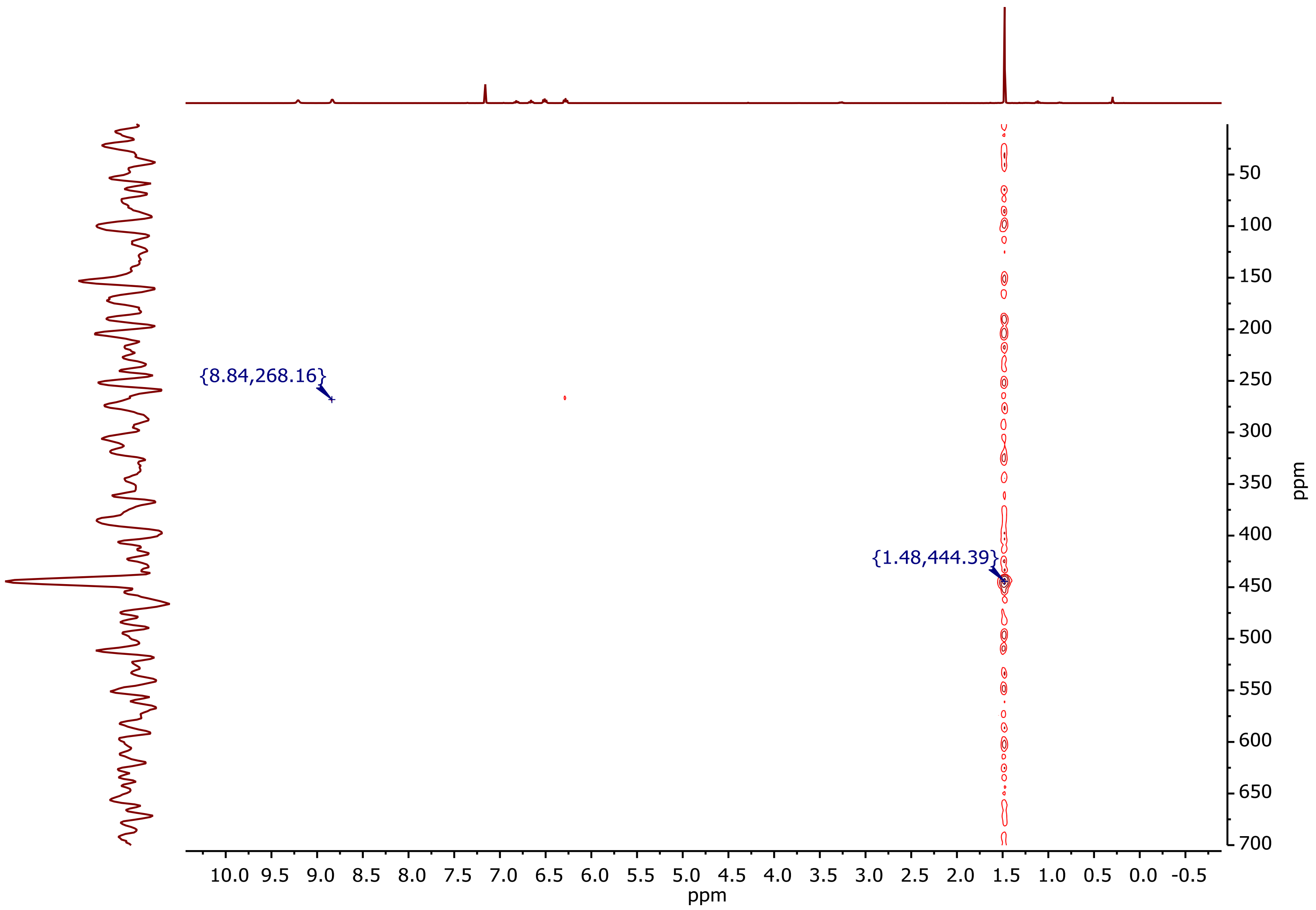


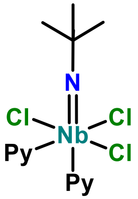


Figure S 2: $^{1}H$-$^{15}N$ HMBC of **Nb(N$^{t}$Bu)Cl$_3$Py$_2$** in $C_6D_6$ at 298 K. (41 MHz)

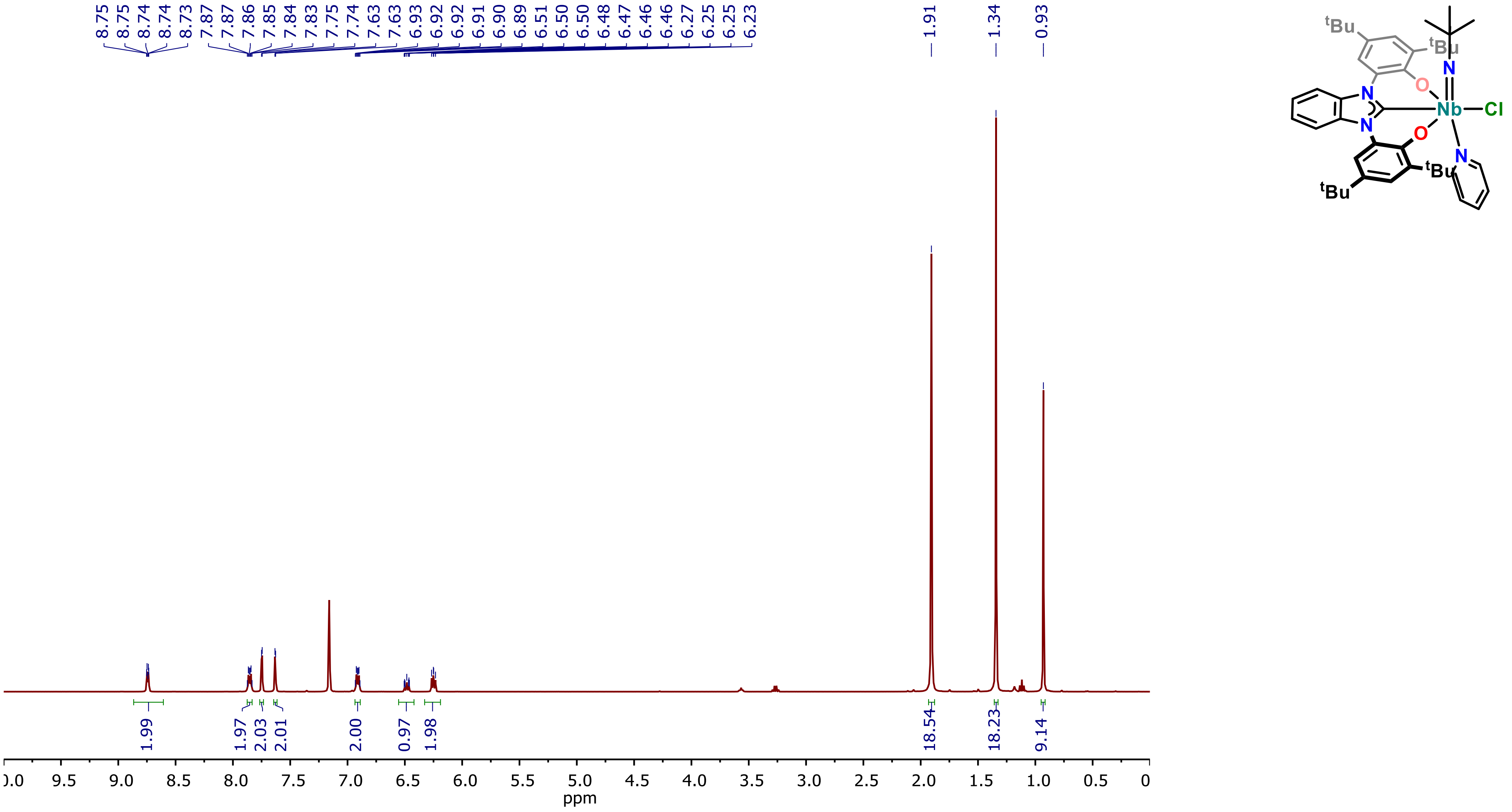


Figure S 3: $^{1}$H NMR of **1-Py** in $C_6D_6$ at 298 K. (400 MHz)

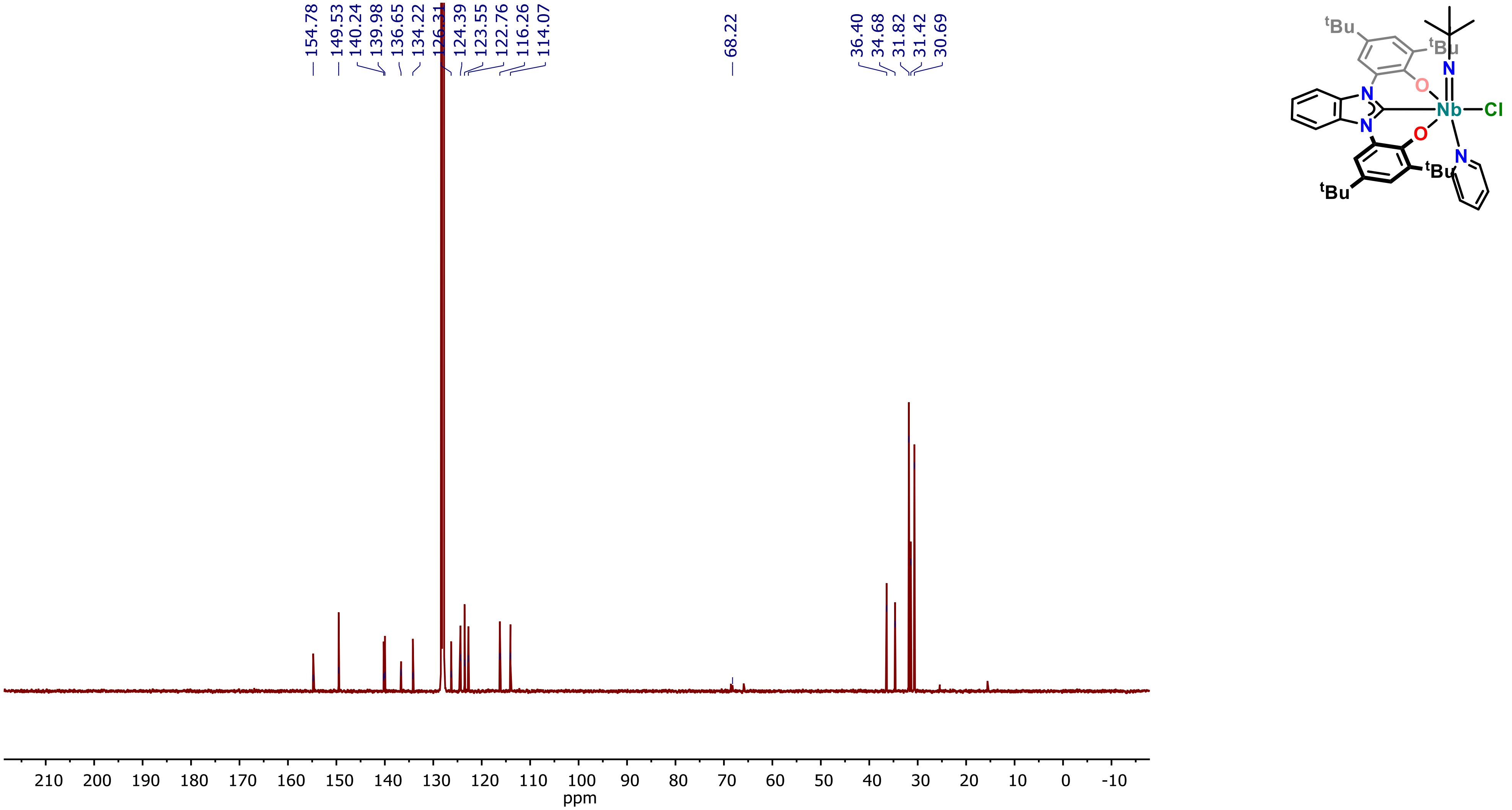


Figure S 4: $^{13}C\{^{1}H\}$ NMR of **1-Py** in $C_6D_6$ at 298 K. (101 MHz)

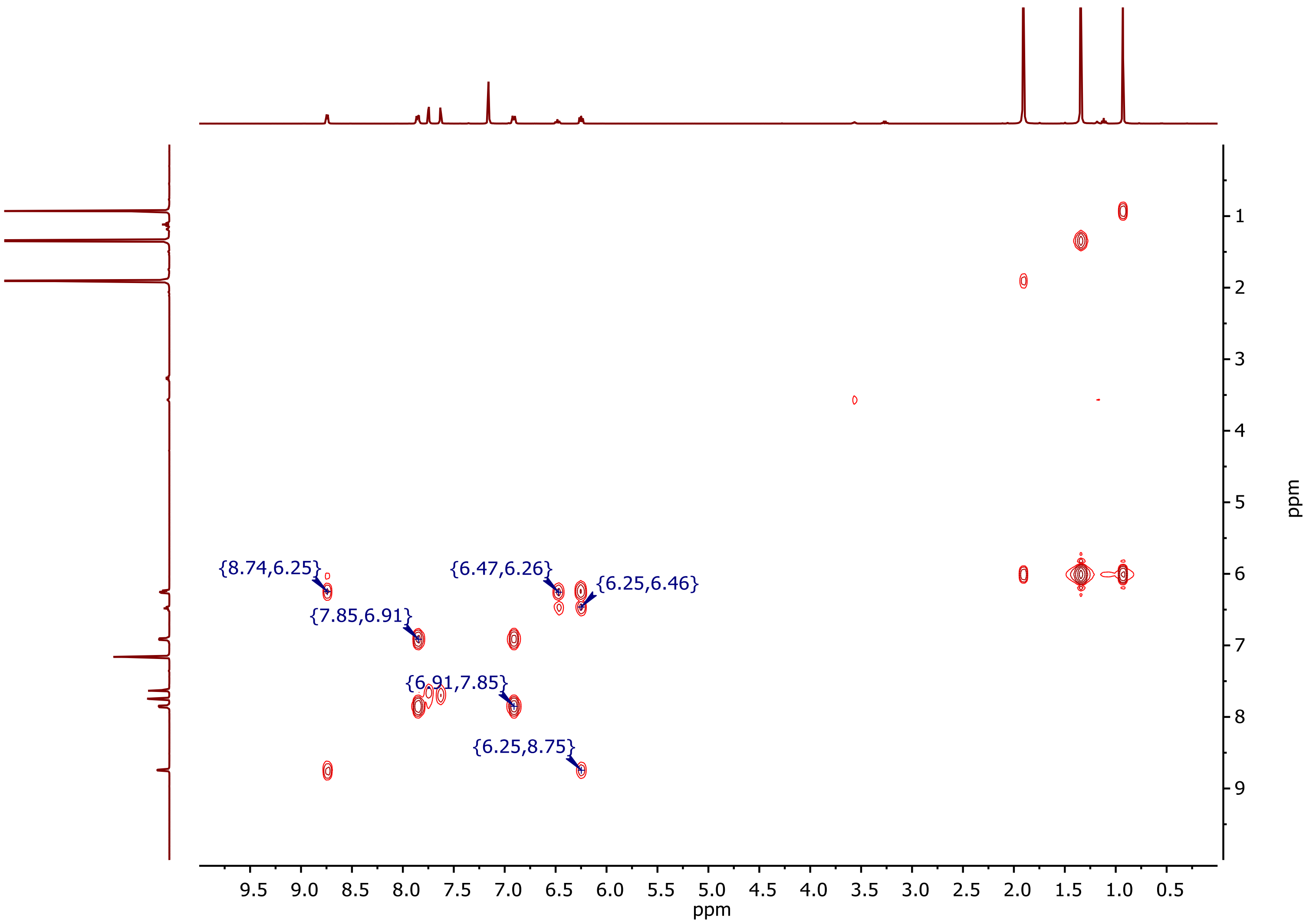


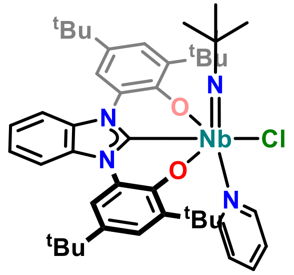


Figure S 5: $^{1}H$-$^{1}H$ COSY of **1-Py** in $C_6D_6$ at 298 K.

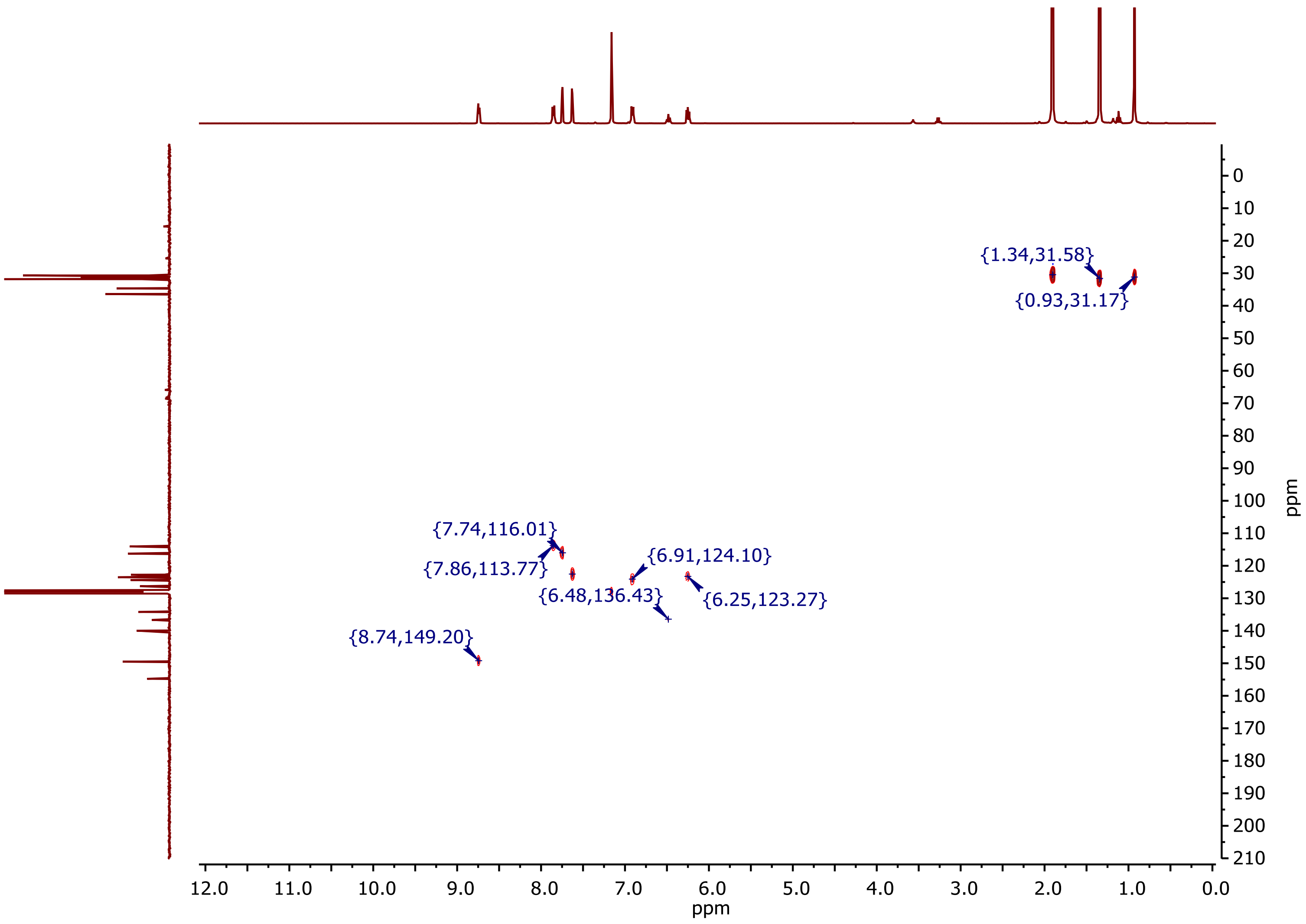


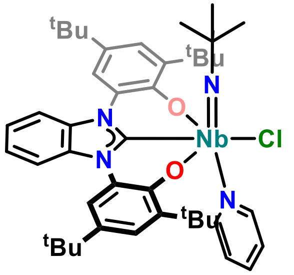


Figure S 6: $^{1}H$-$^{13}C\{^{1}H\}$ HSQC of **1-Py** in $C_6D_6$ at 298 K.

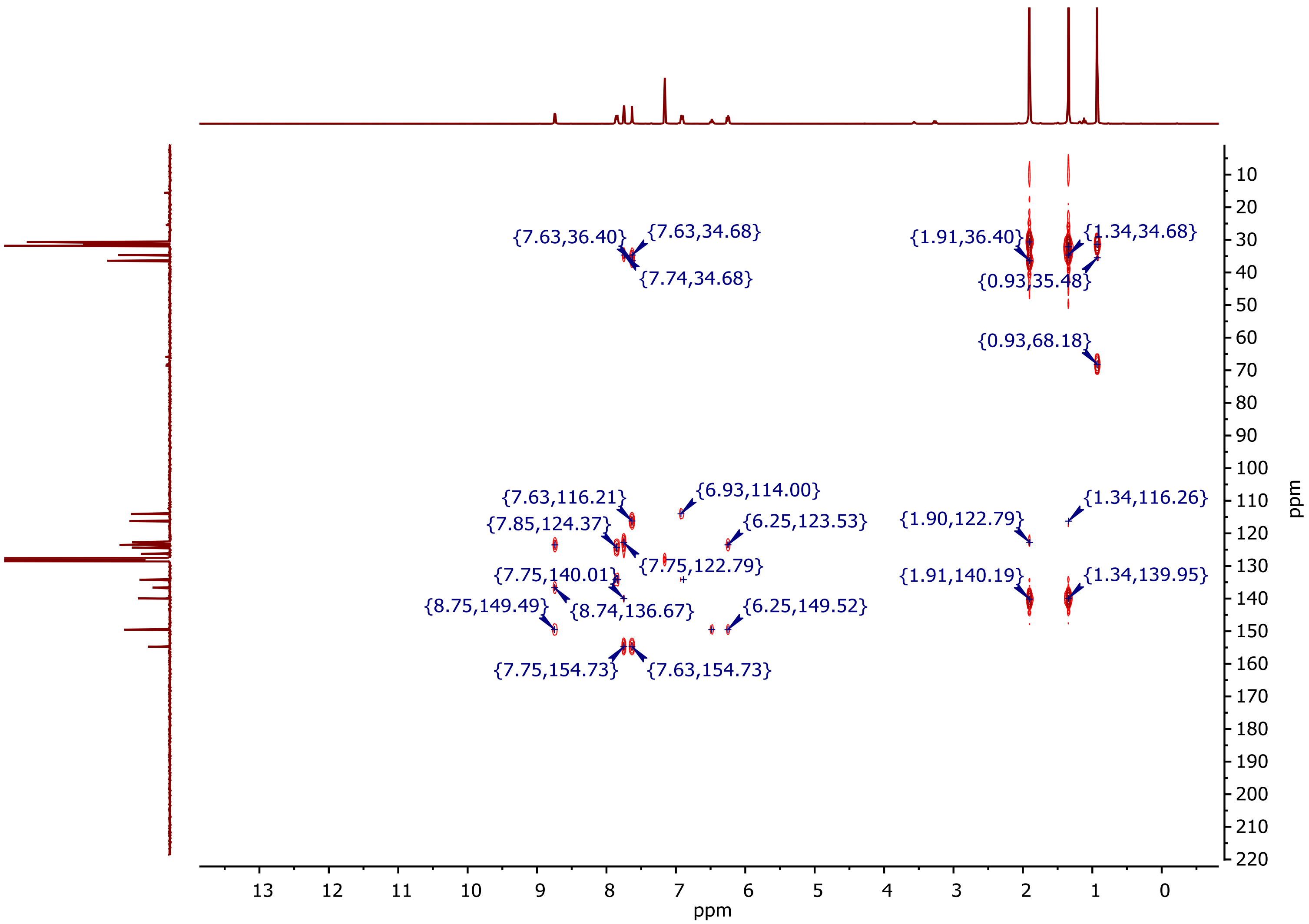


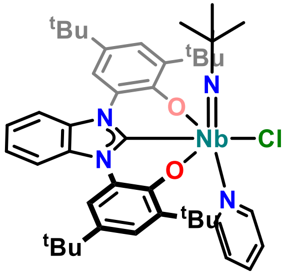


Figure S 7: $^{1}H$-$^{13}C\{^{1}H\}$ HMBC of **1-Py** in $C_6D_6$ at 298K.

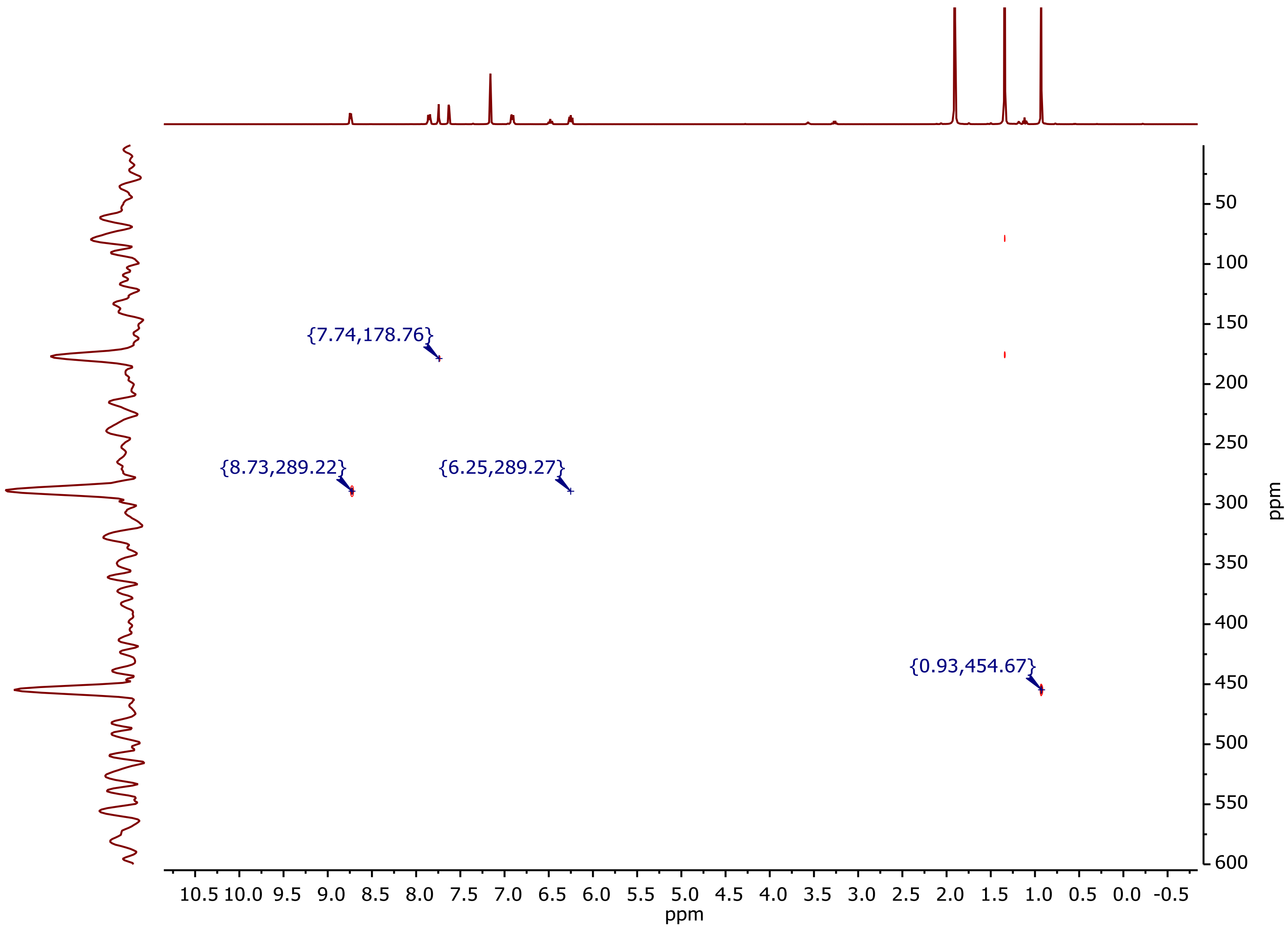


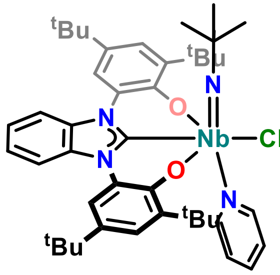


Figure S 8: $^{1}H$-$^{15}N$ HMBC of **1-Py** in $C_6D_6$ at 298K. (41 MHz)

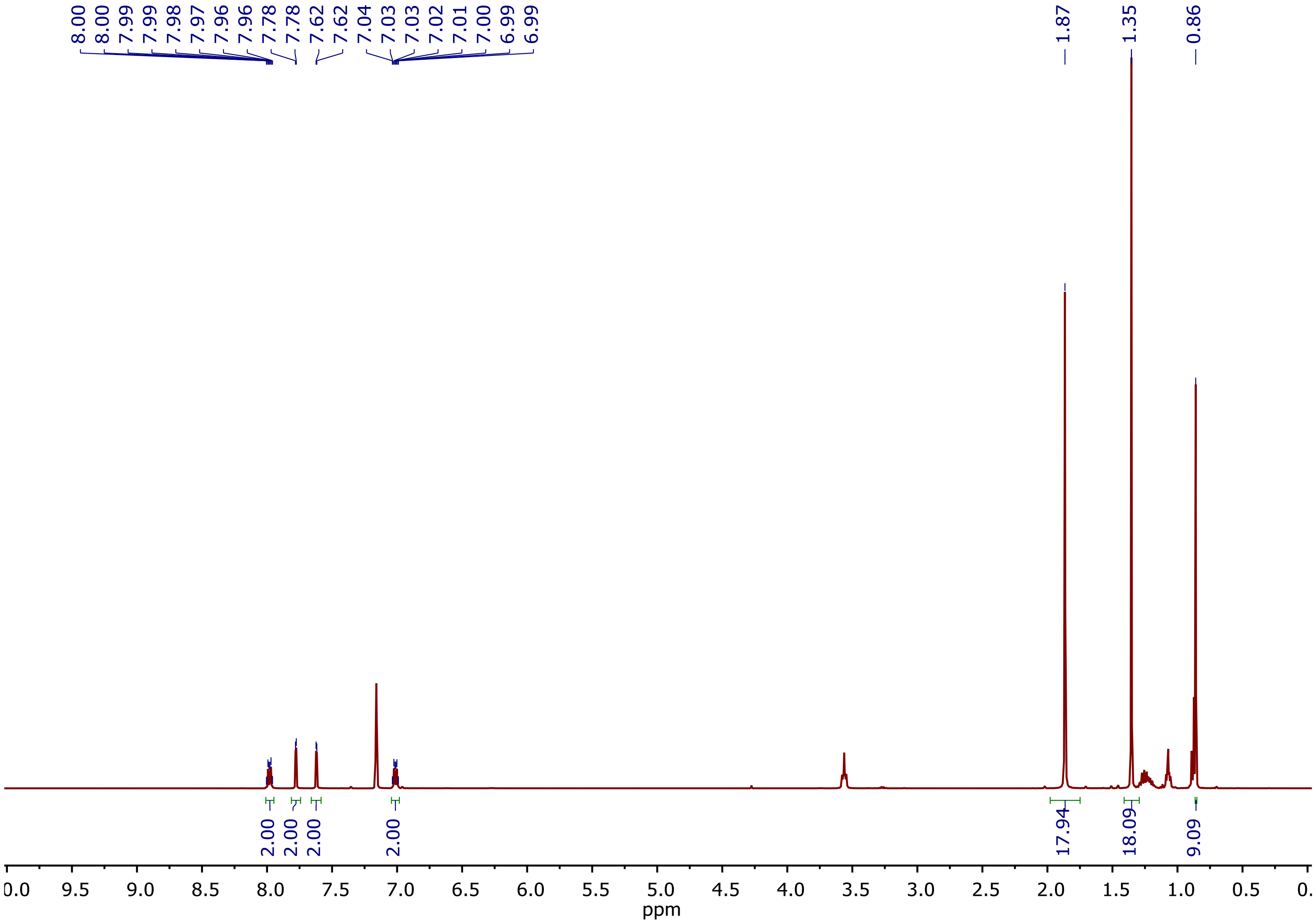


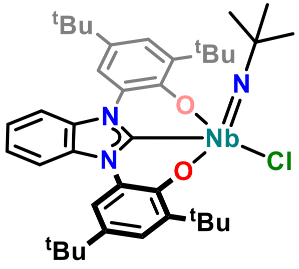


Figure S 9: $^{1}$H NMR of **1** in $C_6D_6$ at 298 K. (400 MHz)

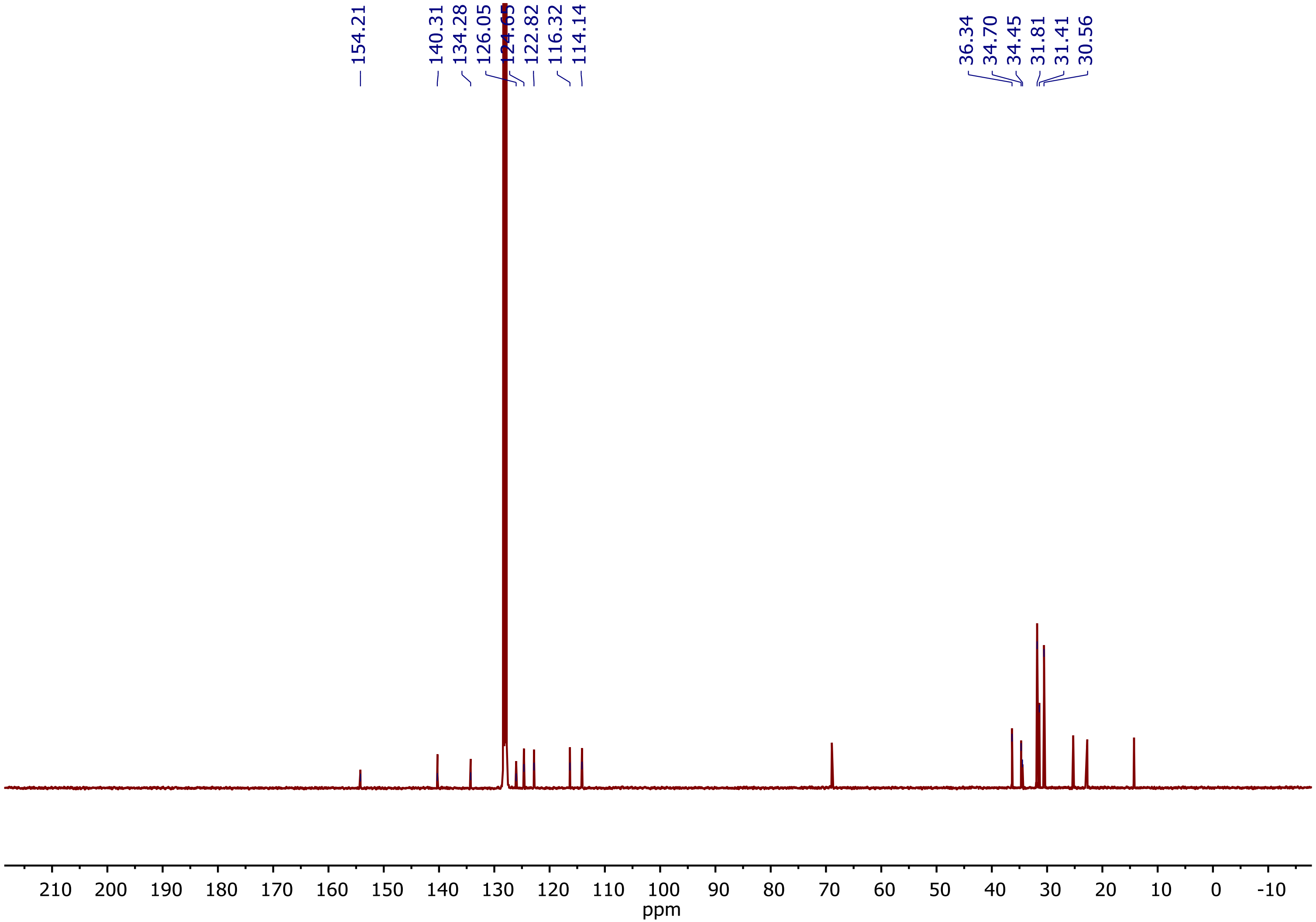


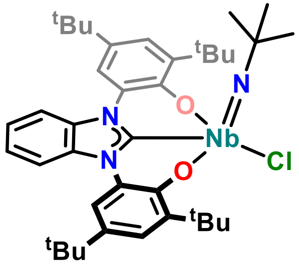


Figure S 10: $^{13}C\{^{1}H\}$ NMR of **1** in $C_6D_6$ at 298 K. (101 MHz)

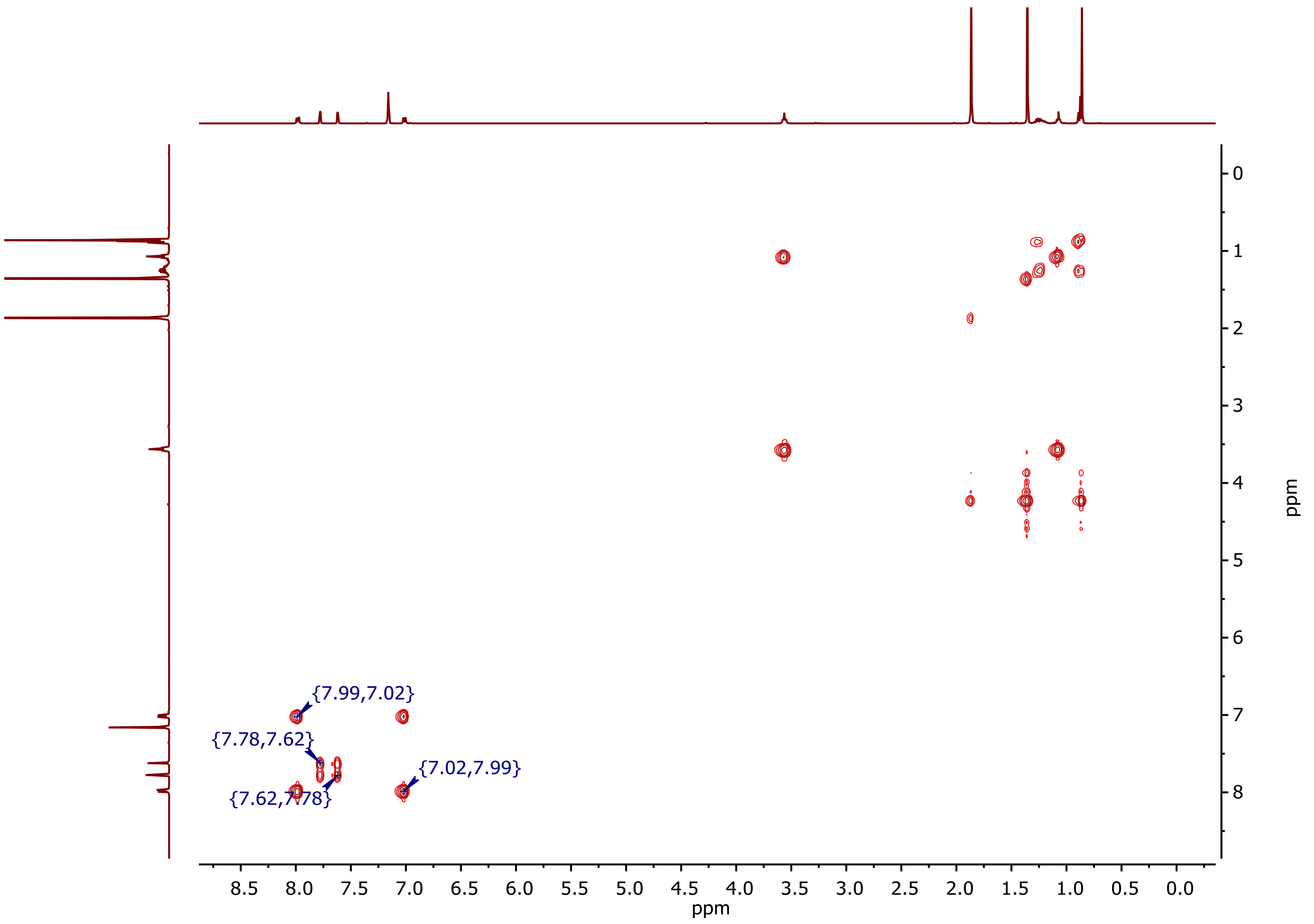


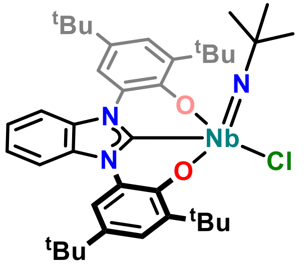


Figure S 11: $^{1}H$-$^{1}H$ COSY of **1** in $C_6D_6$ at 298 K.

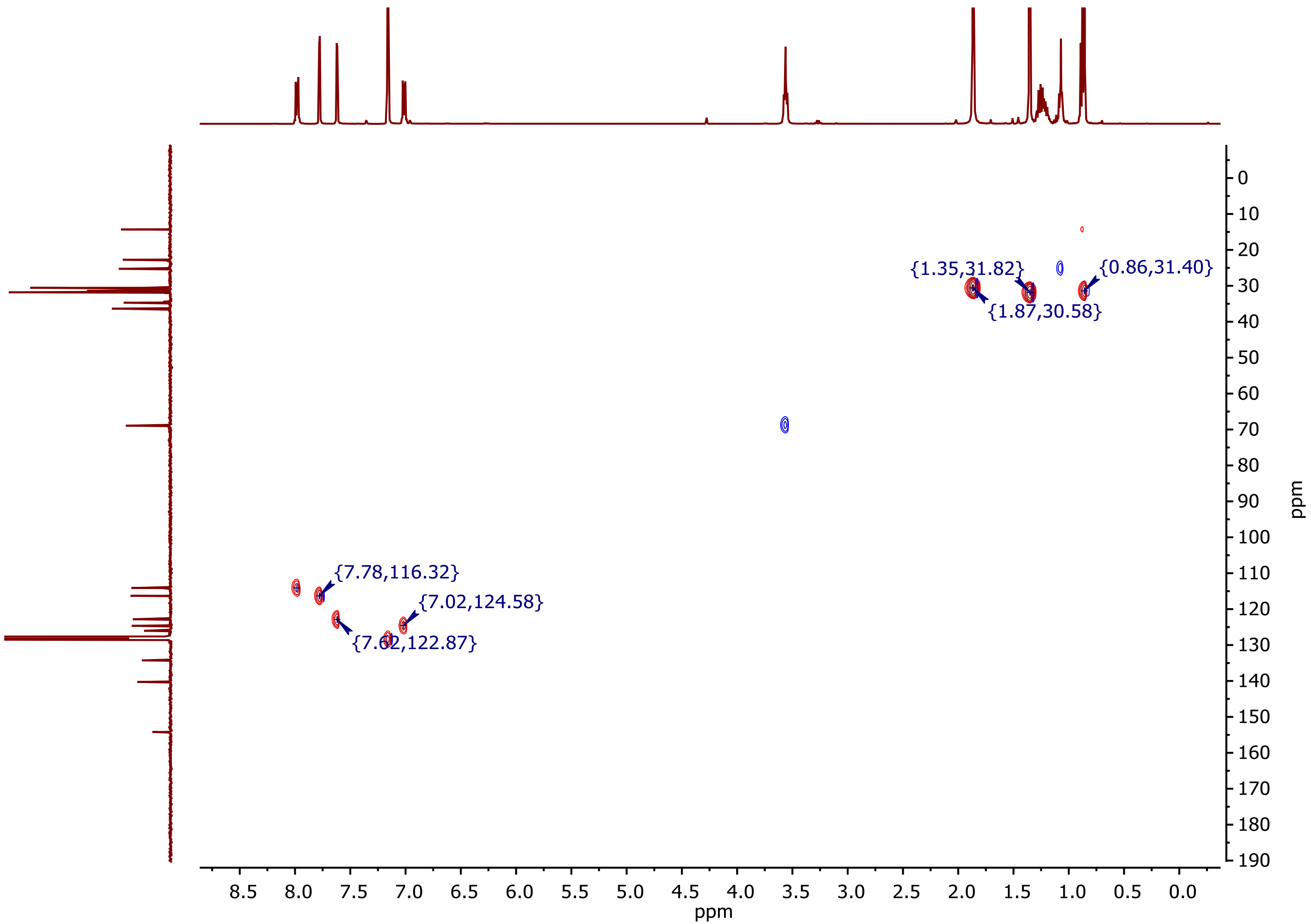


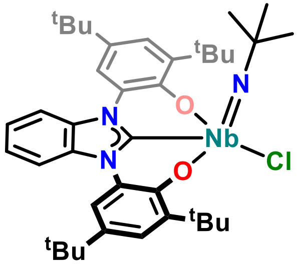


Figure S 12: $^{1}H$-$^{13}C\{^{1}H\}$ HSQC of **1** in $C_6D_6$ at 298 K.

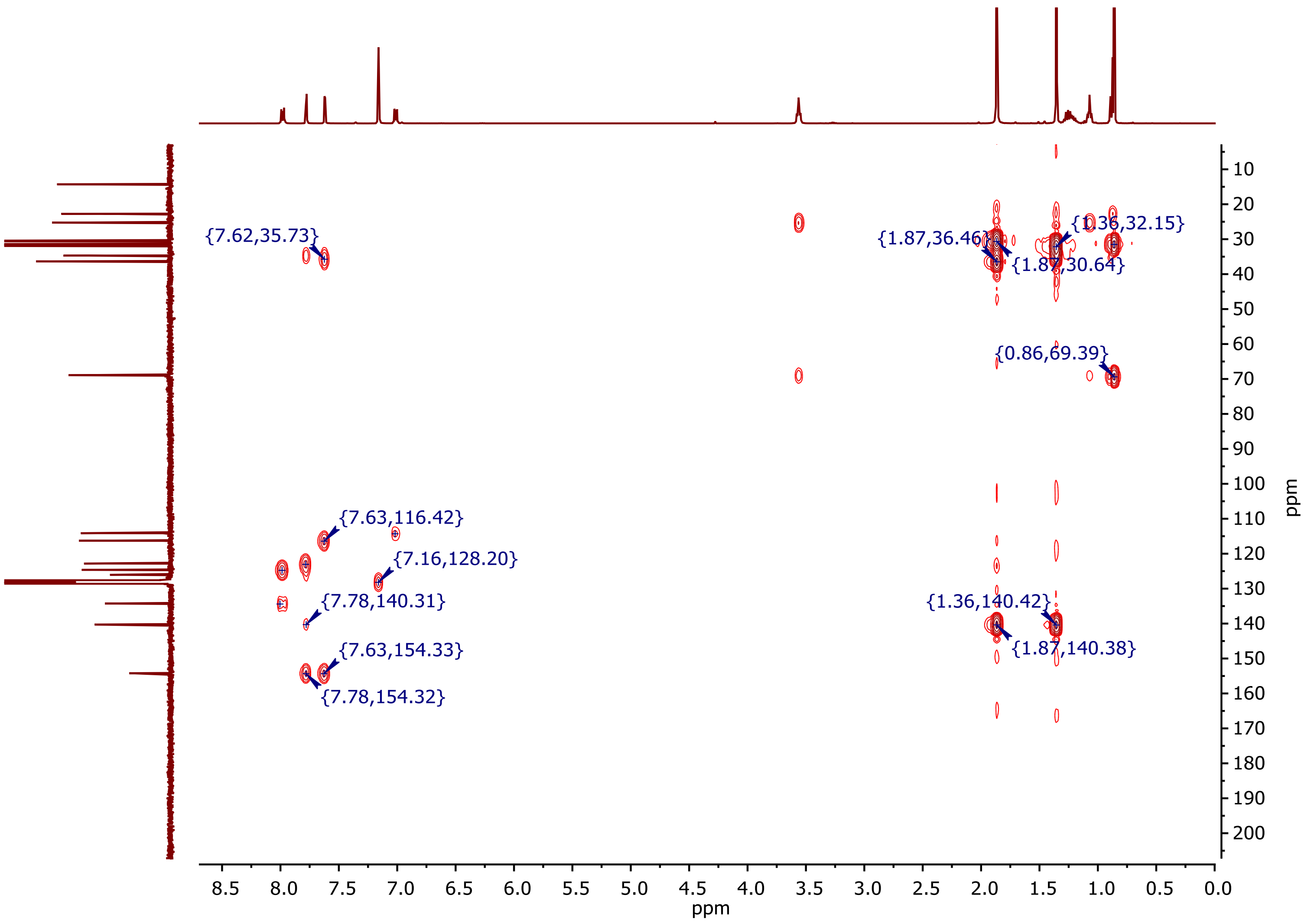


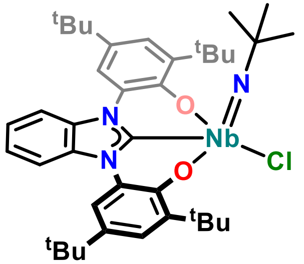


Figure S 13: $^1H$-$^{13}C\{^1H\}$ HMBC of **1** in $C_6D_6$ at 298K.

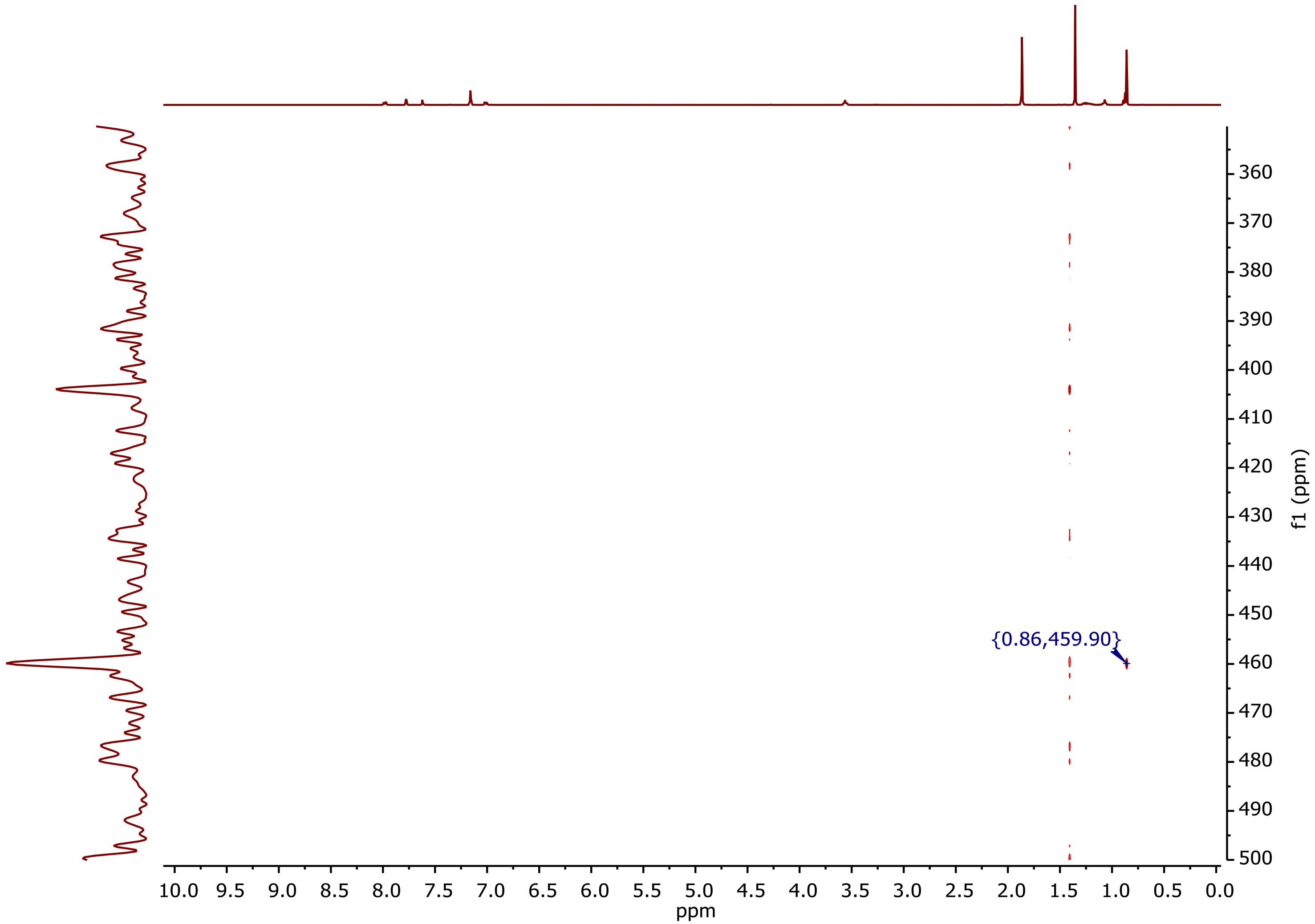


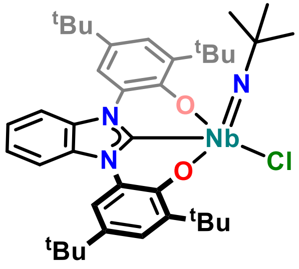


Figure S 14: $^{1}H$-$^{15}N$ HMBC of **1** in $C_6D_6$ at 298K. (41 MHz)

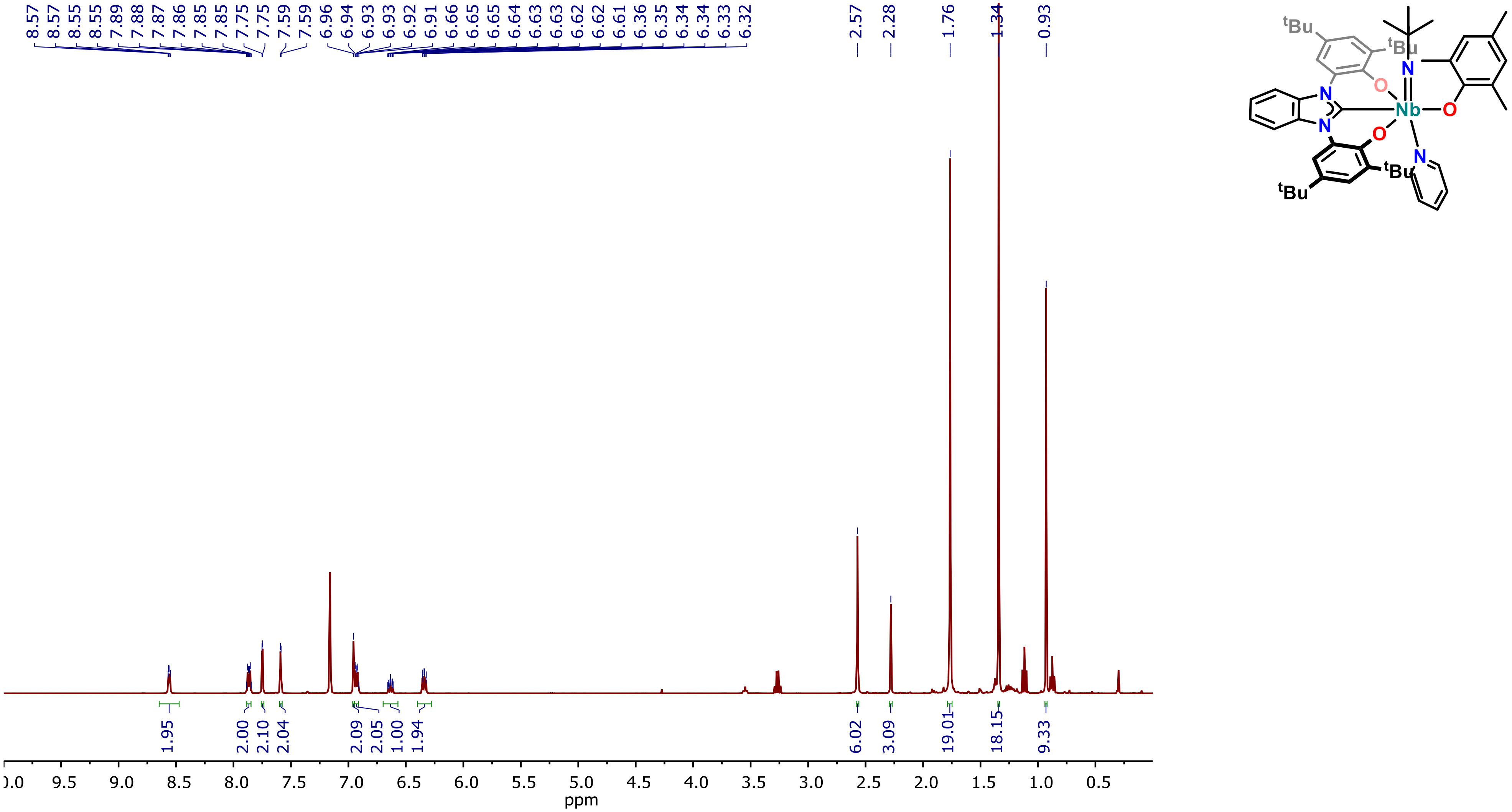


Figure S 15: $^{1}$H NMR of **2** in $C_6D_6$ at 298 K. (400 MHz)

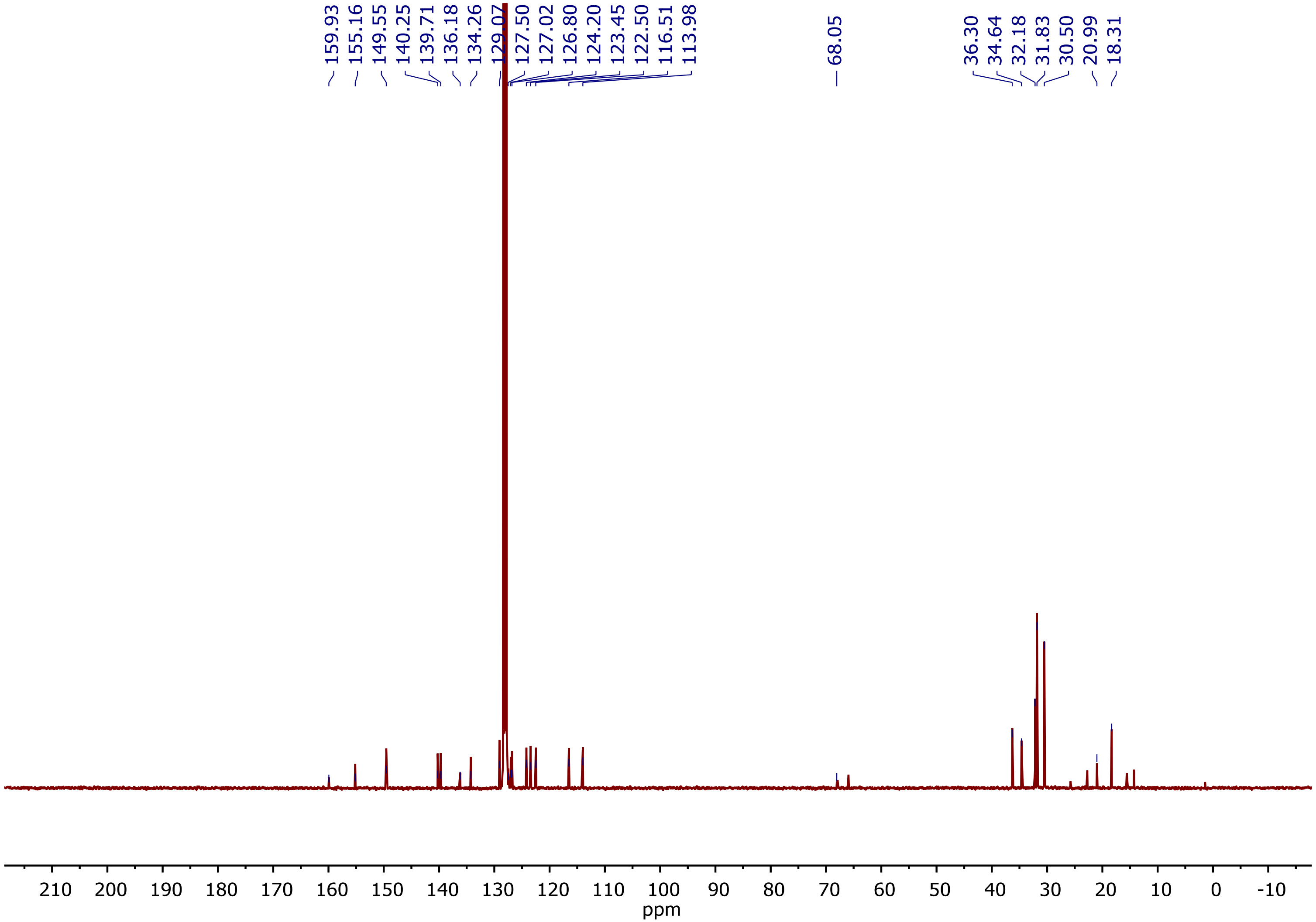


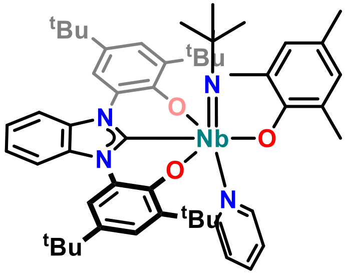


Figure S 16: $^{13}C\{^{1}H\}$ NMR of **2** in $C_6D_6$ at 298 K. (101 MHz)

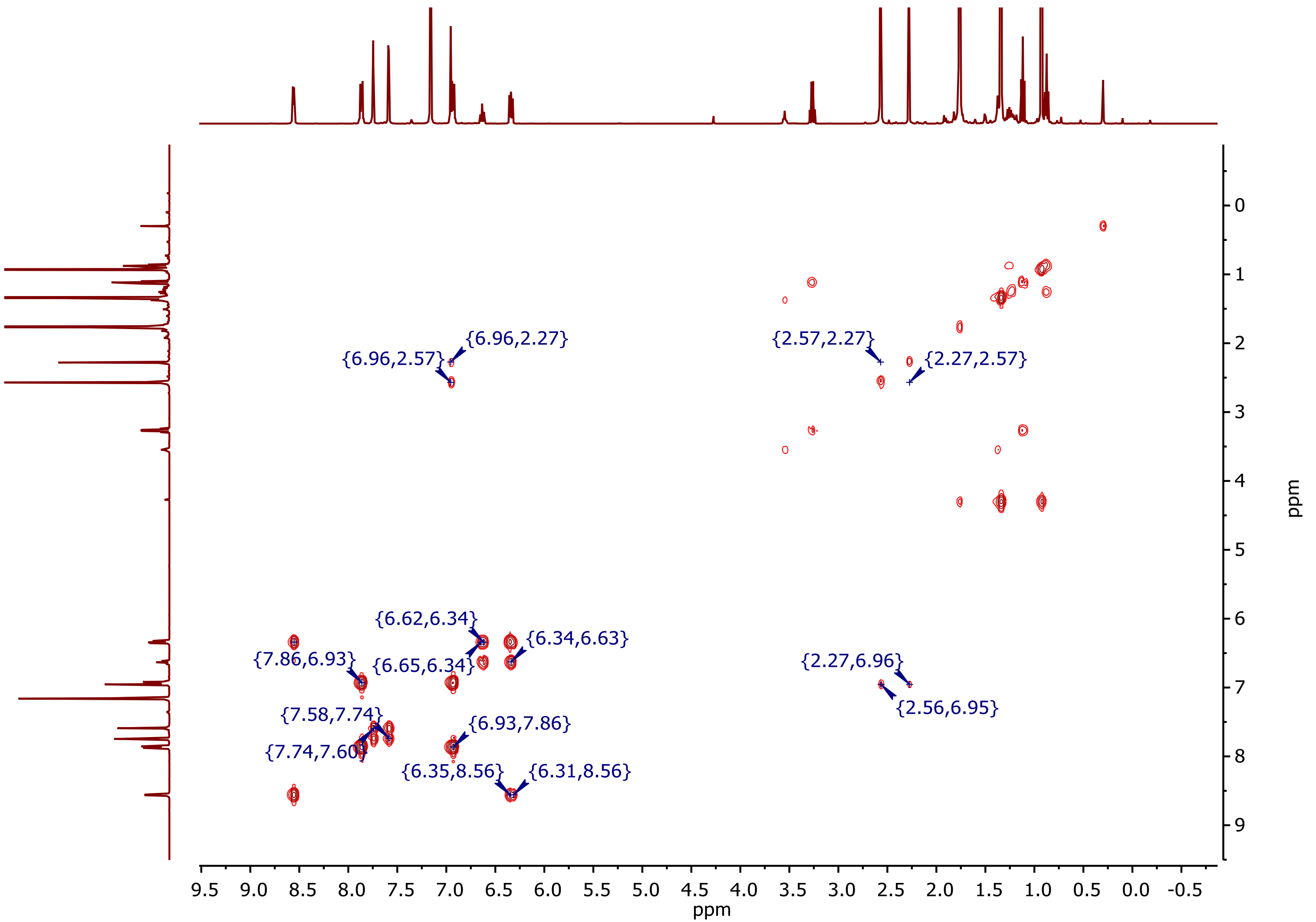


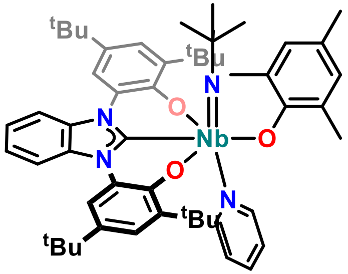


Figure S 17: $^{1}$H-$^{1}$H COSY of **2** in $C_6D_6$ at 298 K.

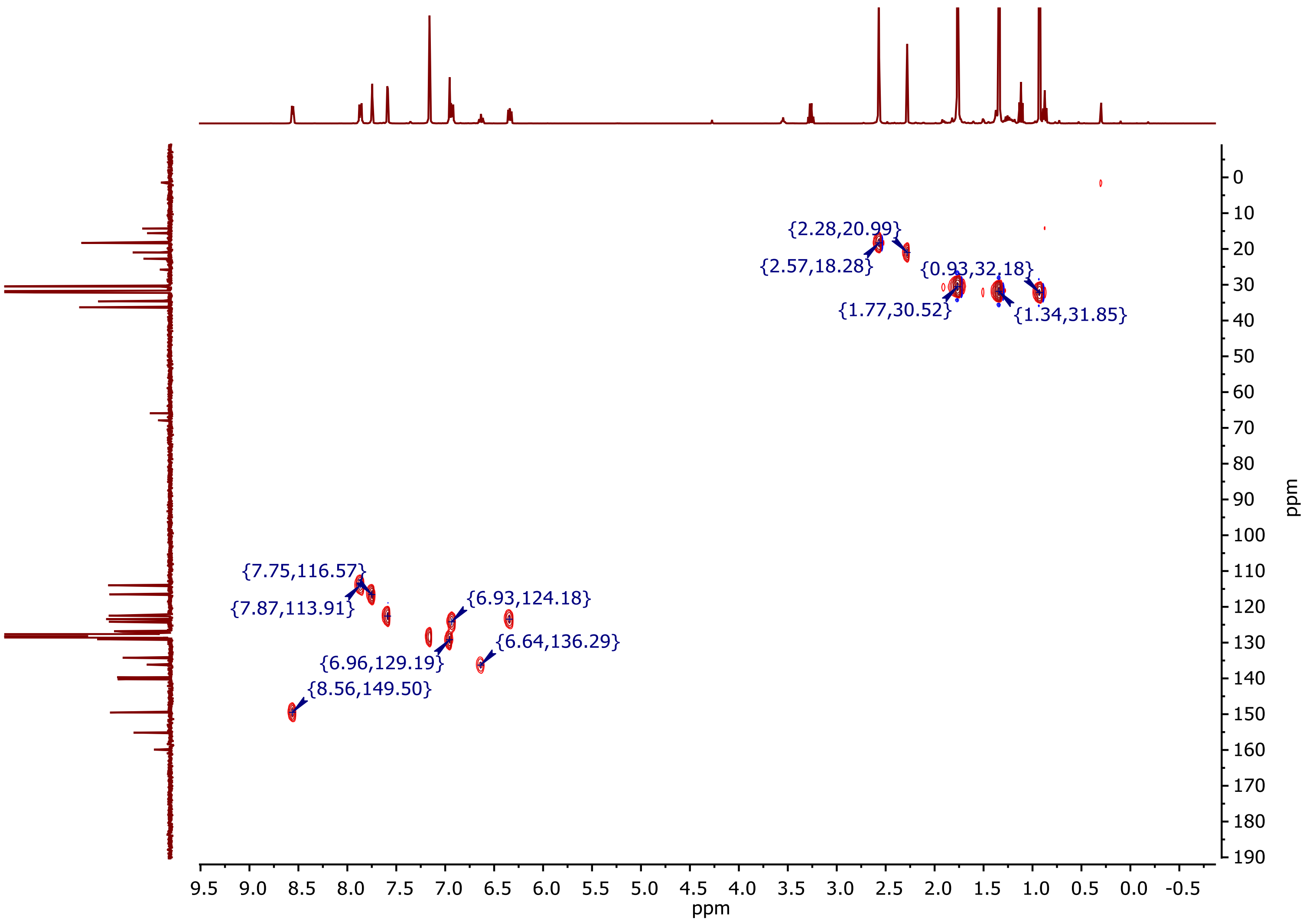


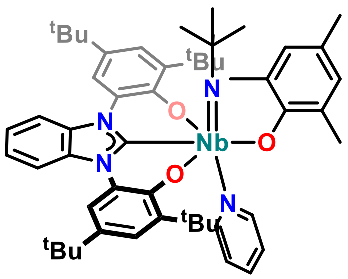


Figure S 18: $^{1}$H-$^{13}$C{$^{1}$H} HSQC of **2** in $C_6D_6$ at 298 K.

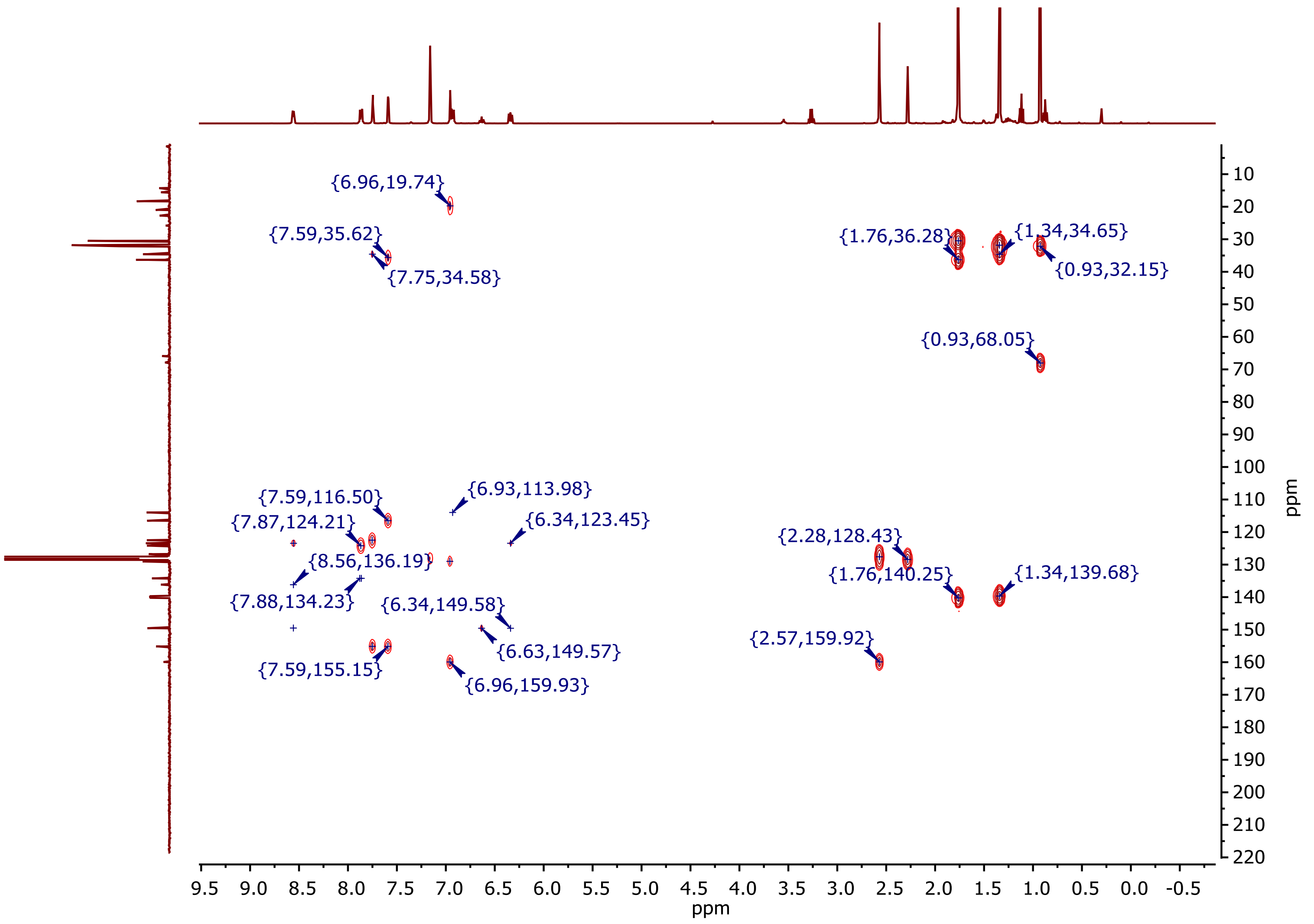


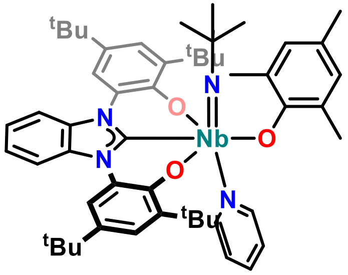


Figure S 19: $^{1}H$-$^{13}C\{^{1}H\}$ HMBC of **2** in $C_6D_6$ at 298K.

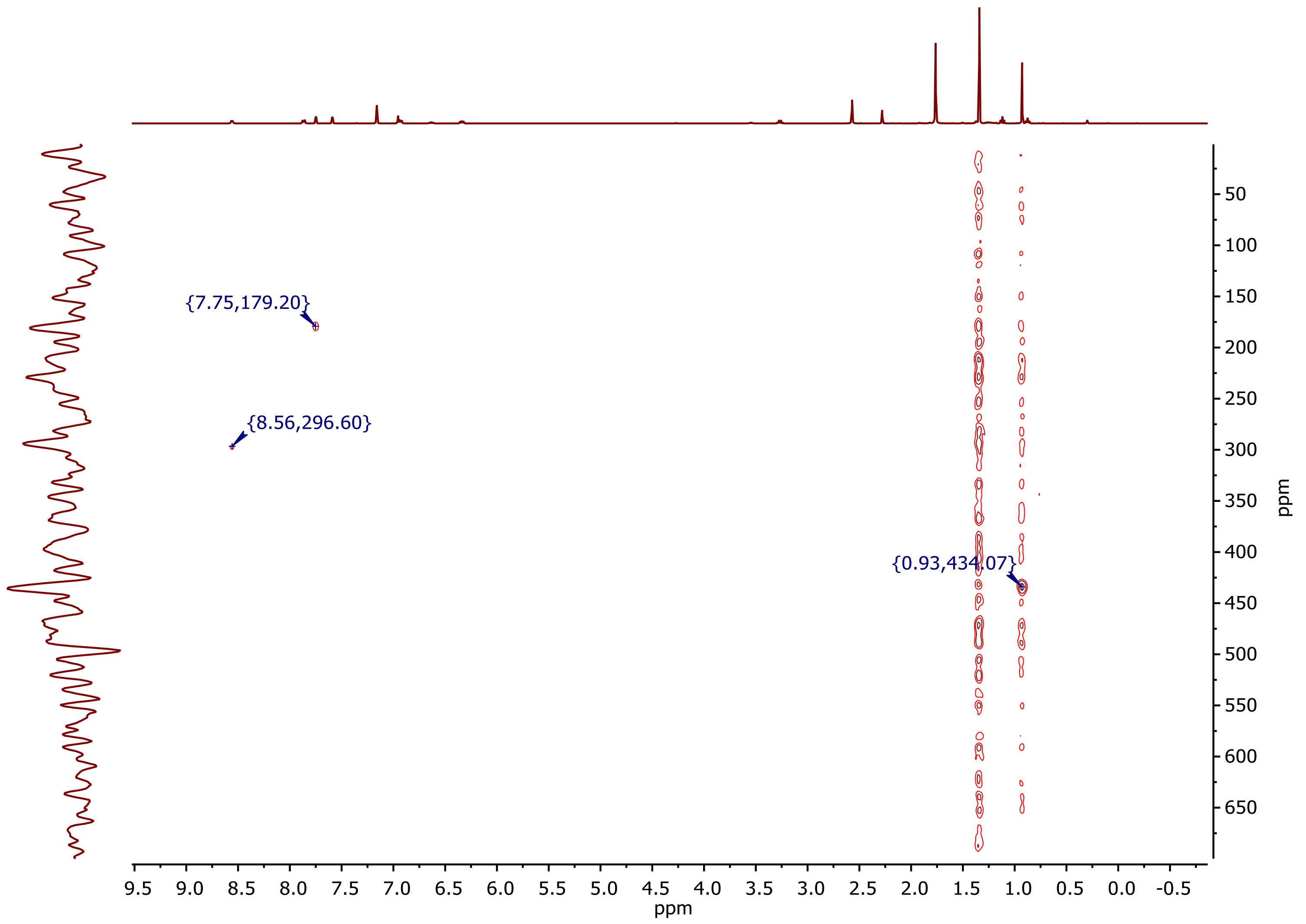


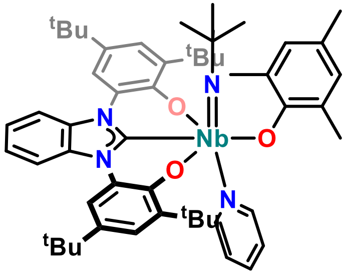


Figure S 20: $^{1}H$-$^{15}N$ HMBC of **2** in $C_6D_6$ at 298K. (41 MHz)

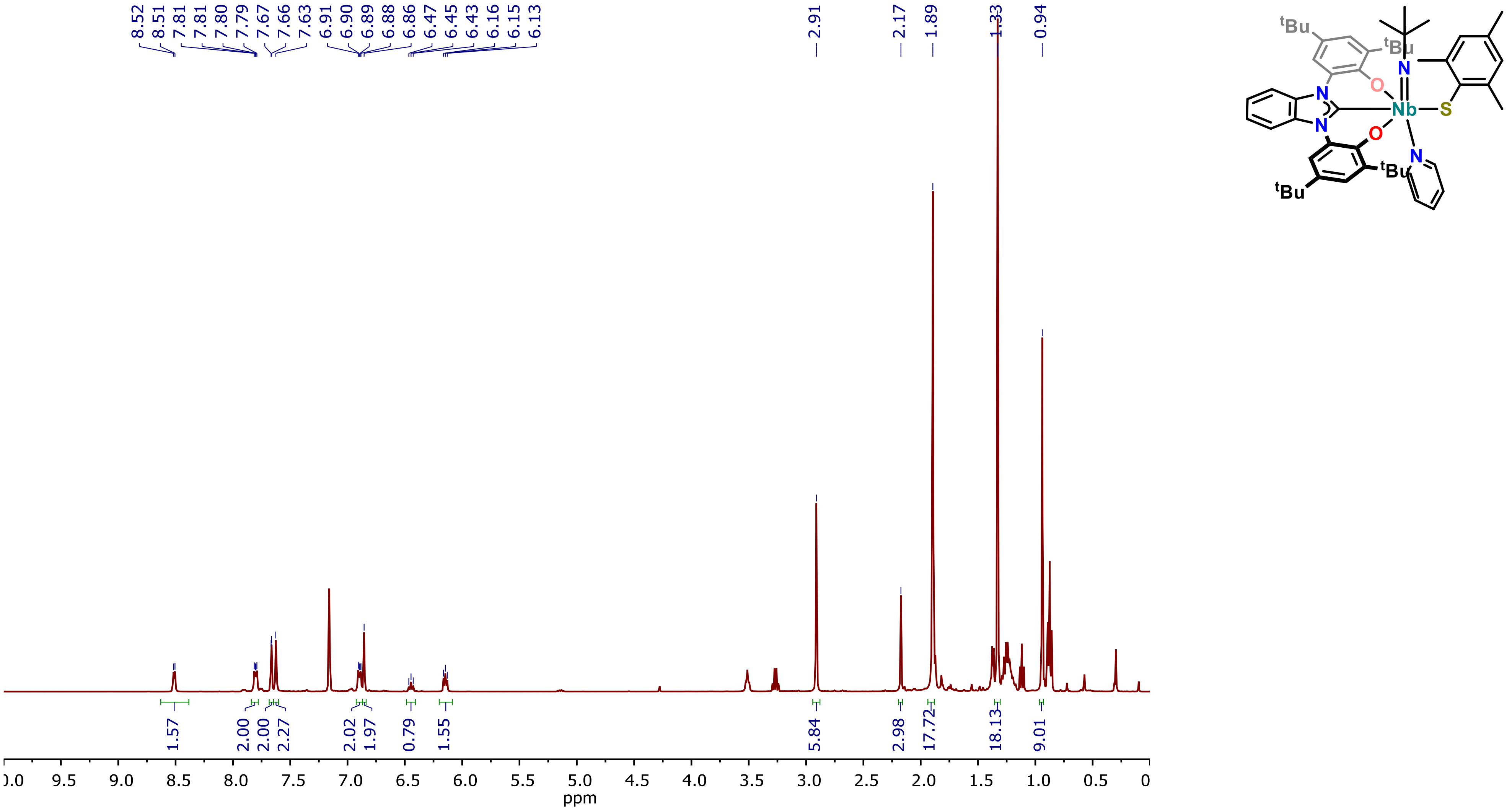


Figure S 21: $^{1}$H NMR of **3** in $C_6D_6$ at 298 K. (400 MHz)

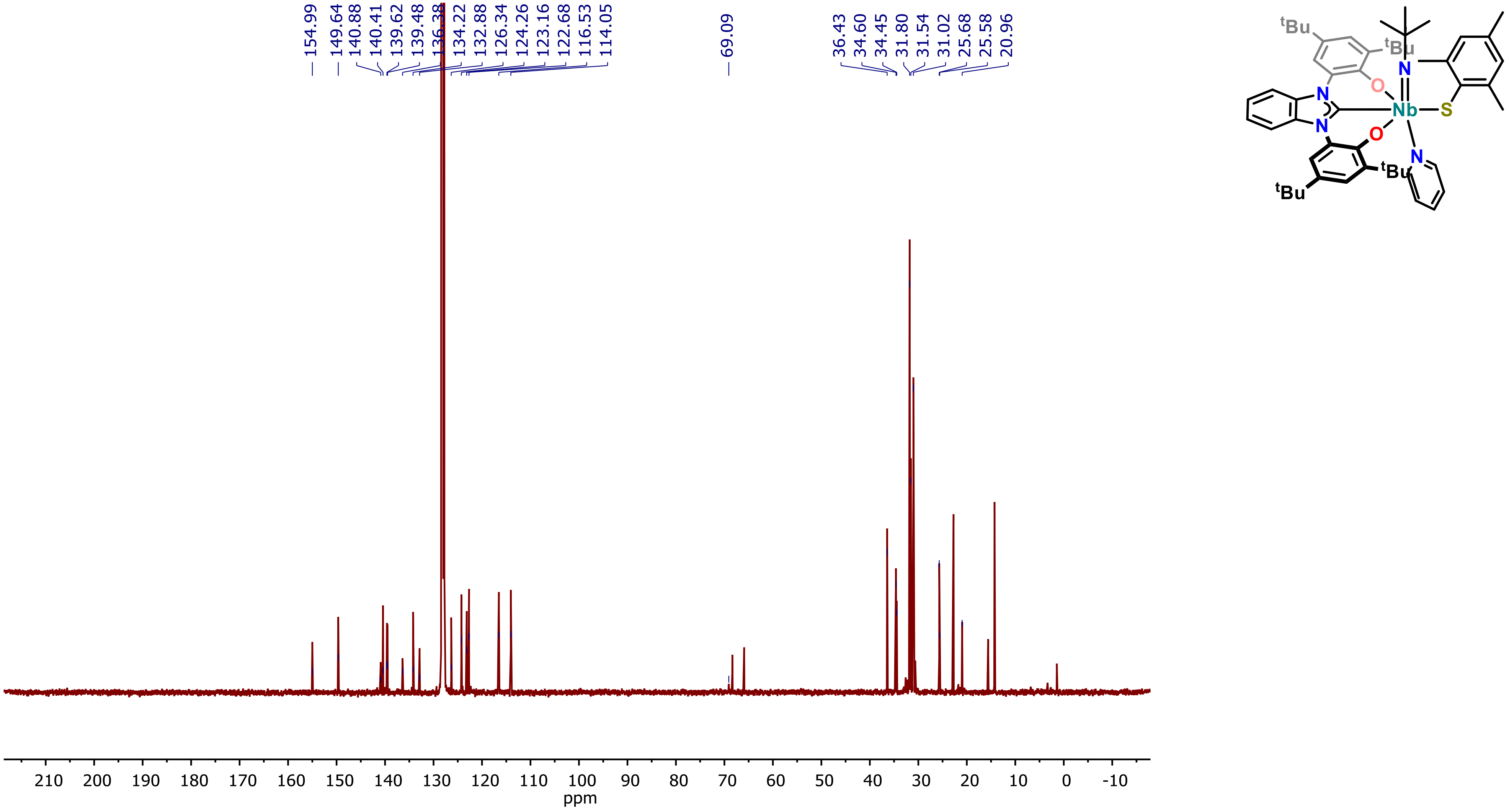


Figure S 22: $^{13}C\{^1H\}$ NMR of **3** in $C_6D_6$ at 298 K. (101 MHz)

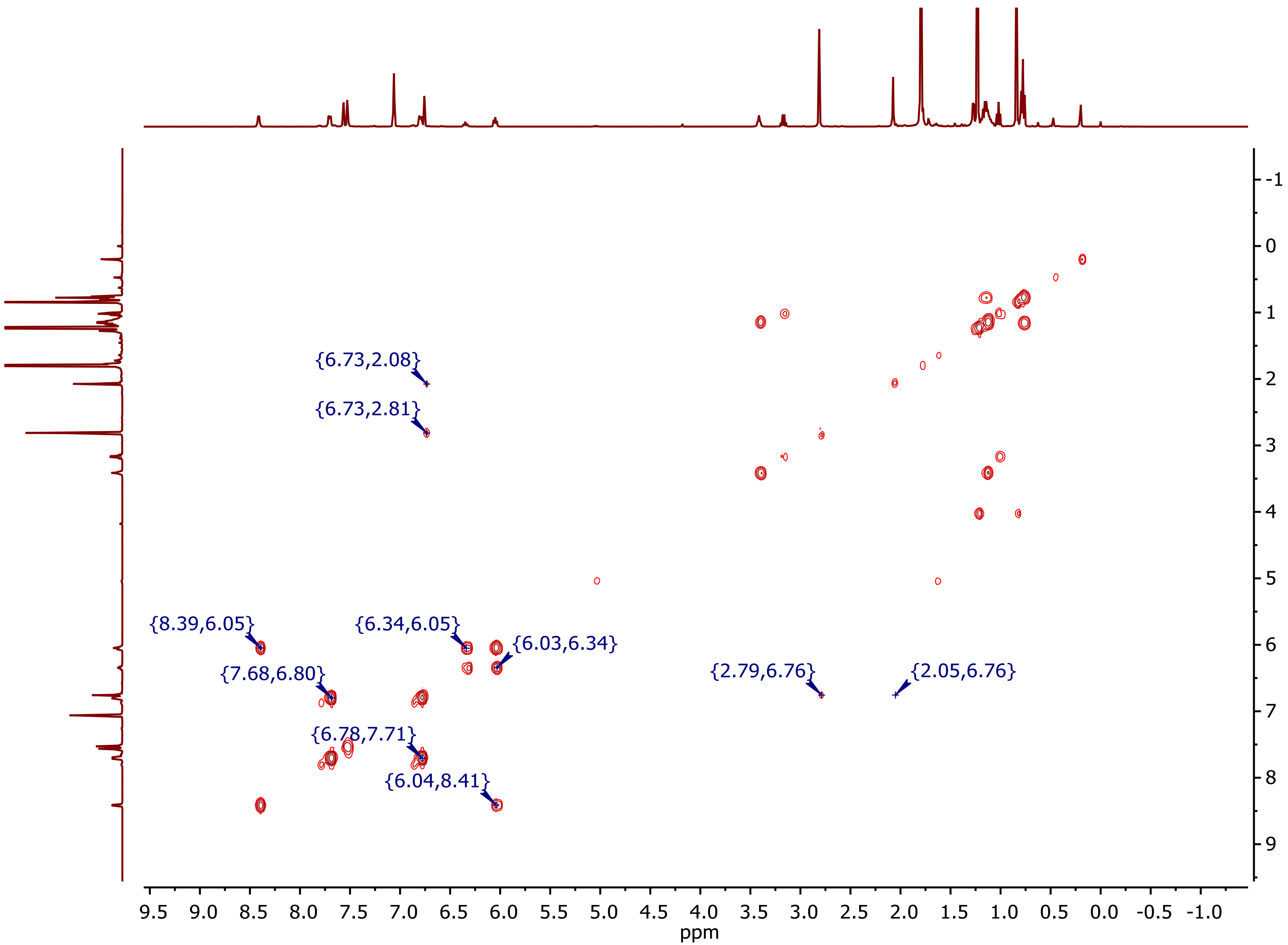


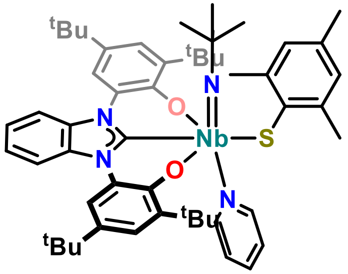


Figure S 23: $^{1}H$-$^{1}H$ COSY of **3** in $C_6D_6$ at 298 K.

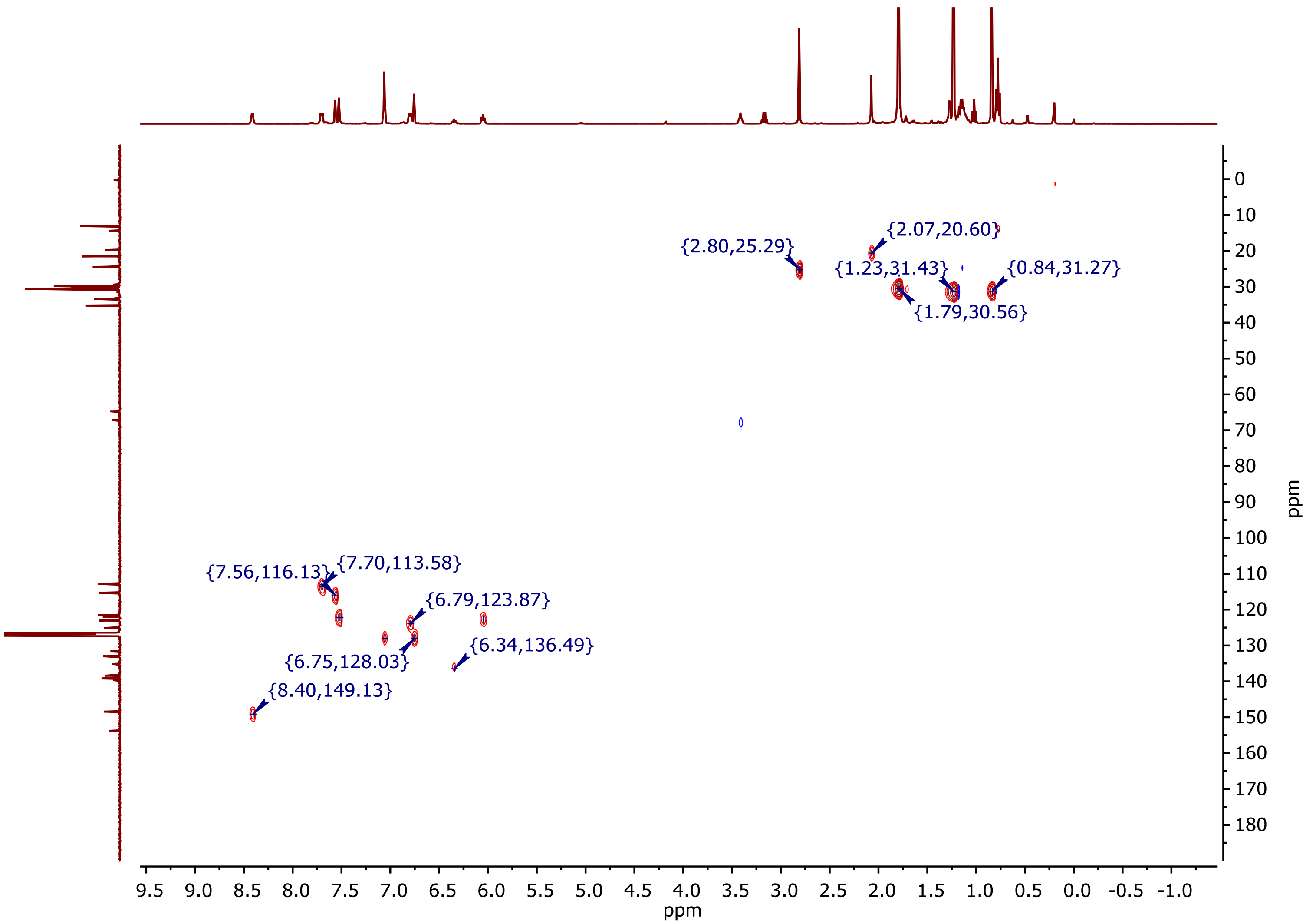


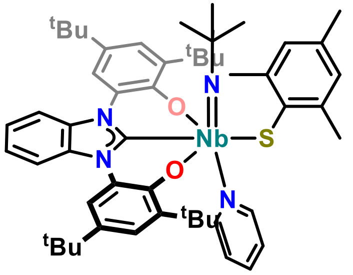


Figure S 24: $^{1}H$-$^{13}C\{^{1}H\}$ HSQC of **3** in $C_6D_6$ at 298 K.

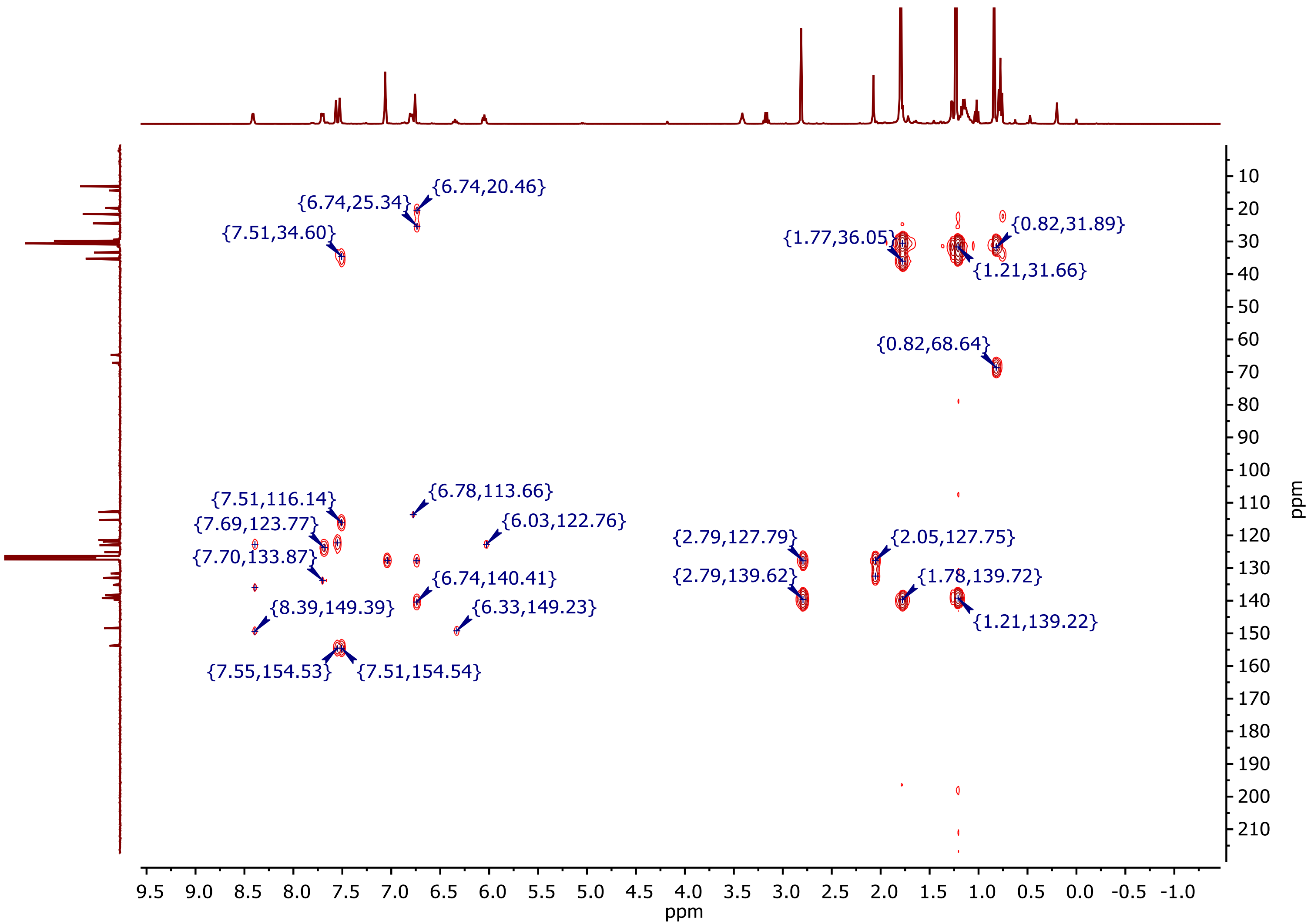


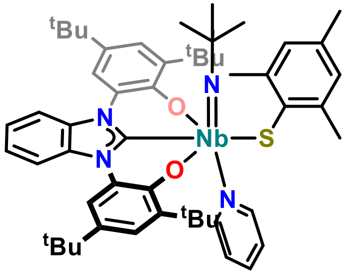


Figure S 25: $^{1}H$-$^{13}C\{^{1}H\}$ HMBC of **3** in $C_6D_6$ at 298K.

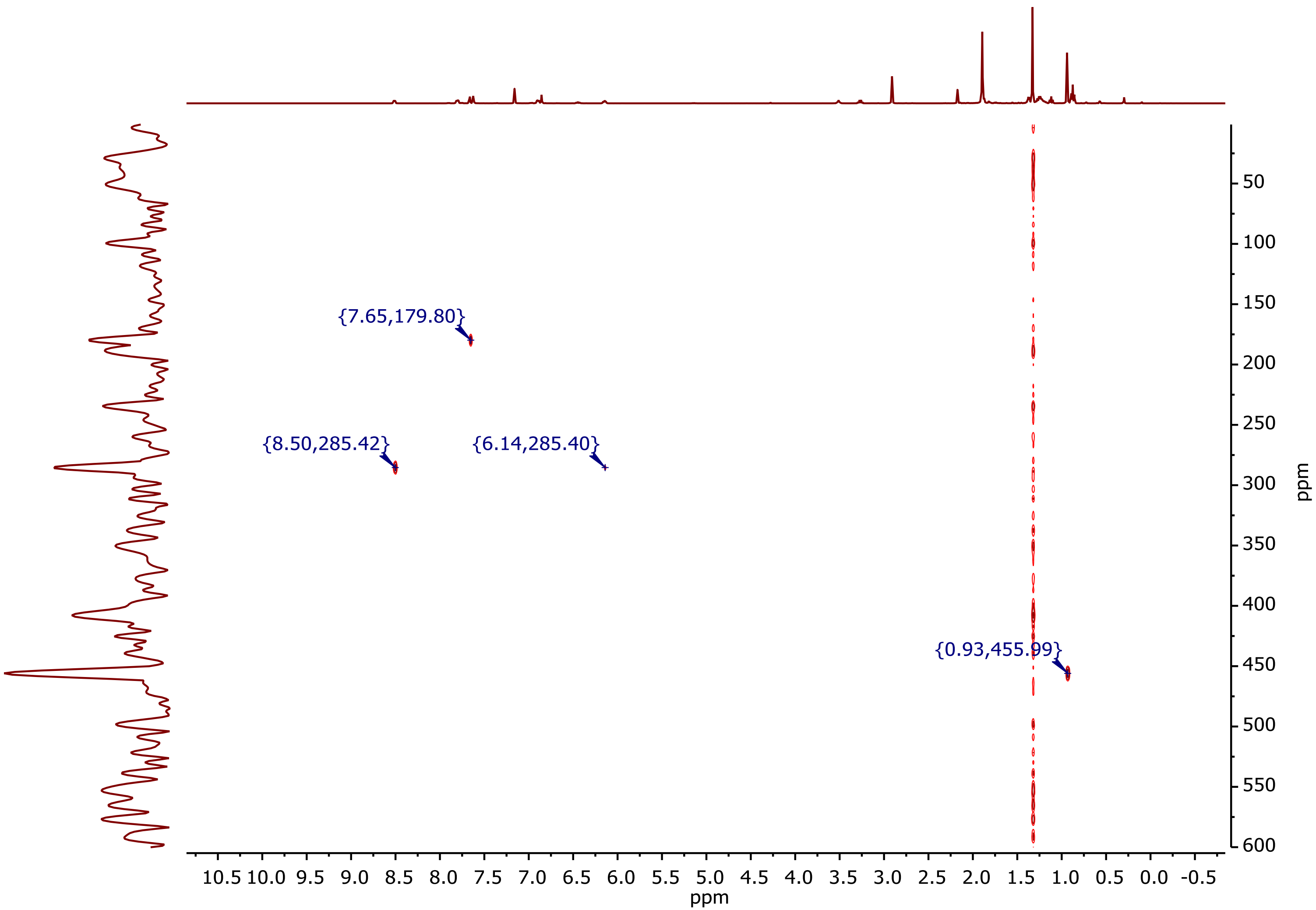


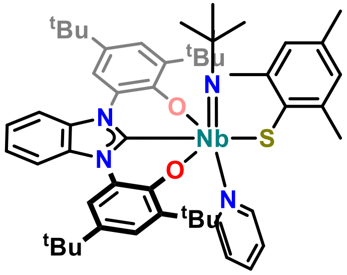


Figure S 26: $^{1}H$-$^{15}N$ HMBC of **3** in $C_6D_6$ at 298K. (41 MHz)

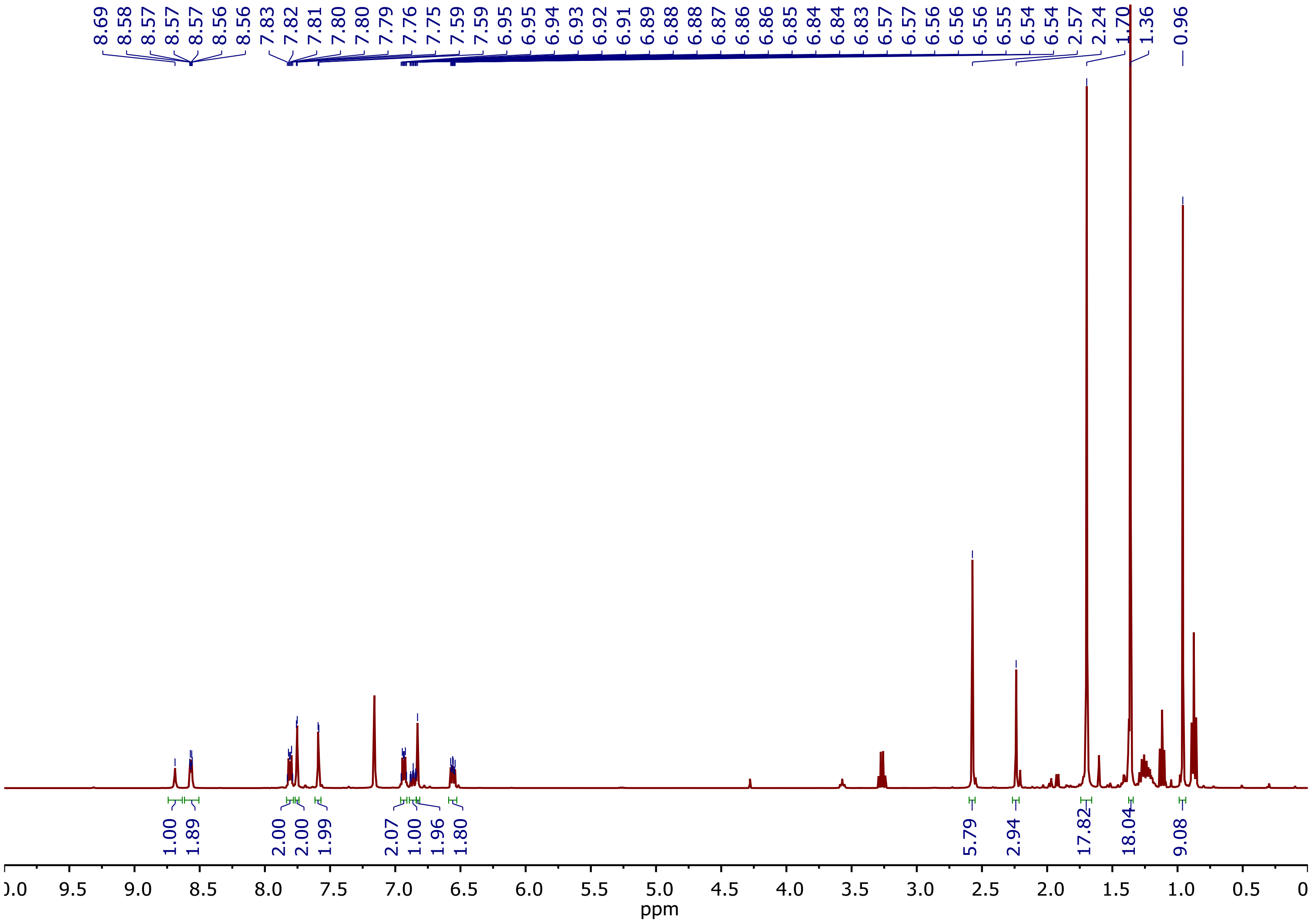

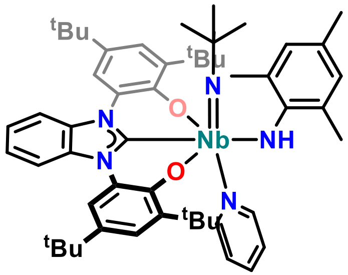


Figure S 27: $^{1}H$ NMR of **4** in $C_6D_6$ at 298 K. (400 MHz)

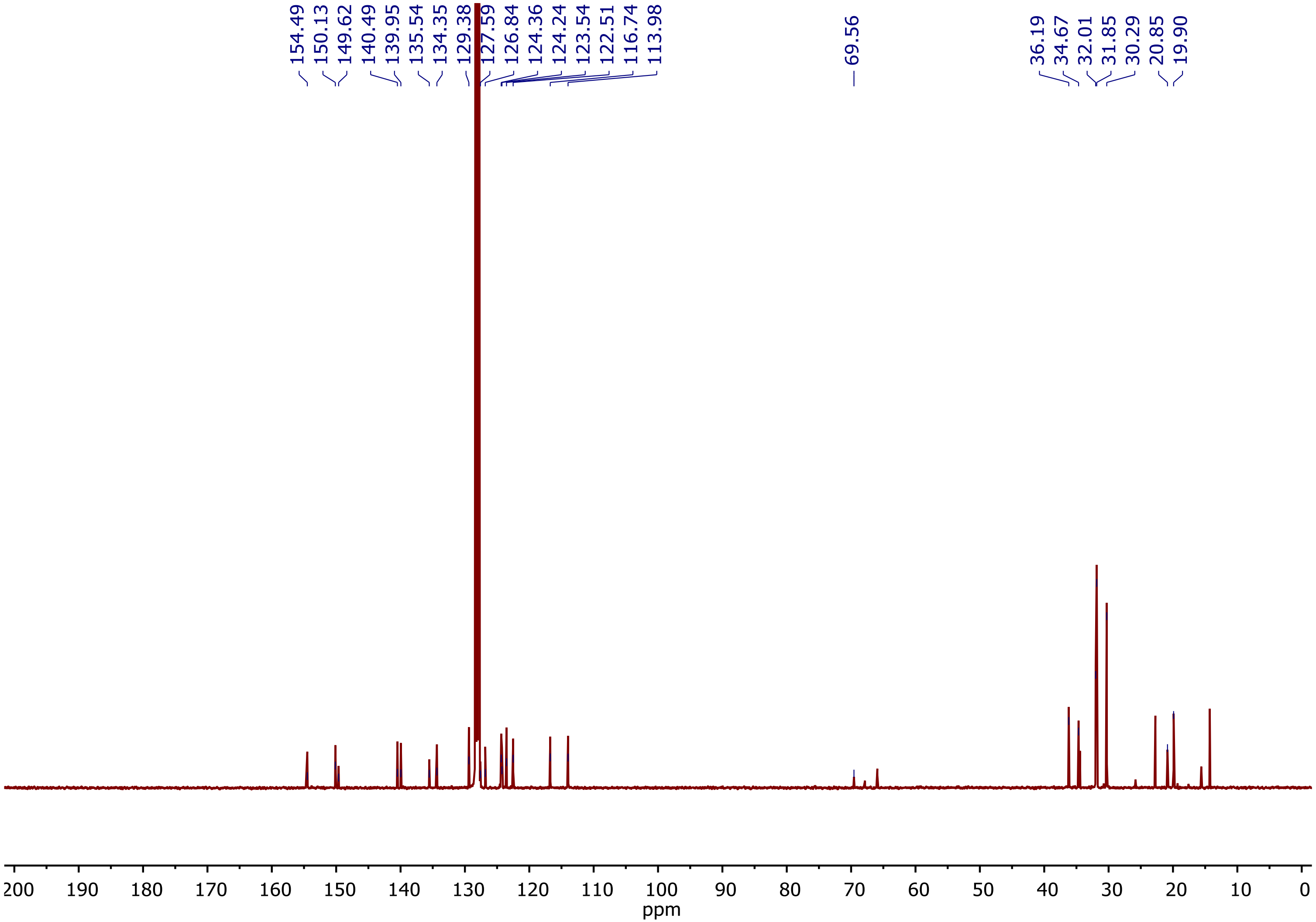


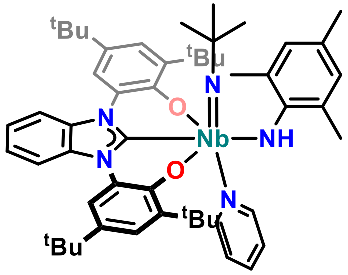


Figure S 28: $^{13}C\{^{1}H\}$ NMR of **4** in $C_6D_6$ at 298 K. (101 MHz)

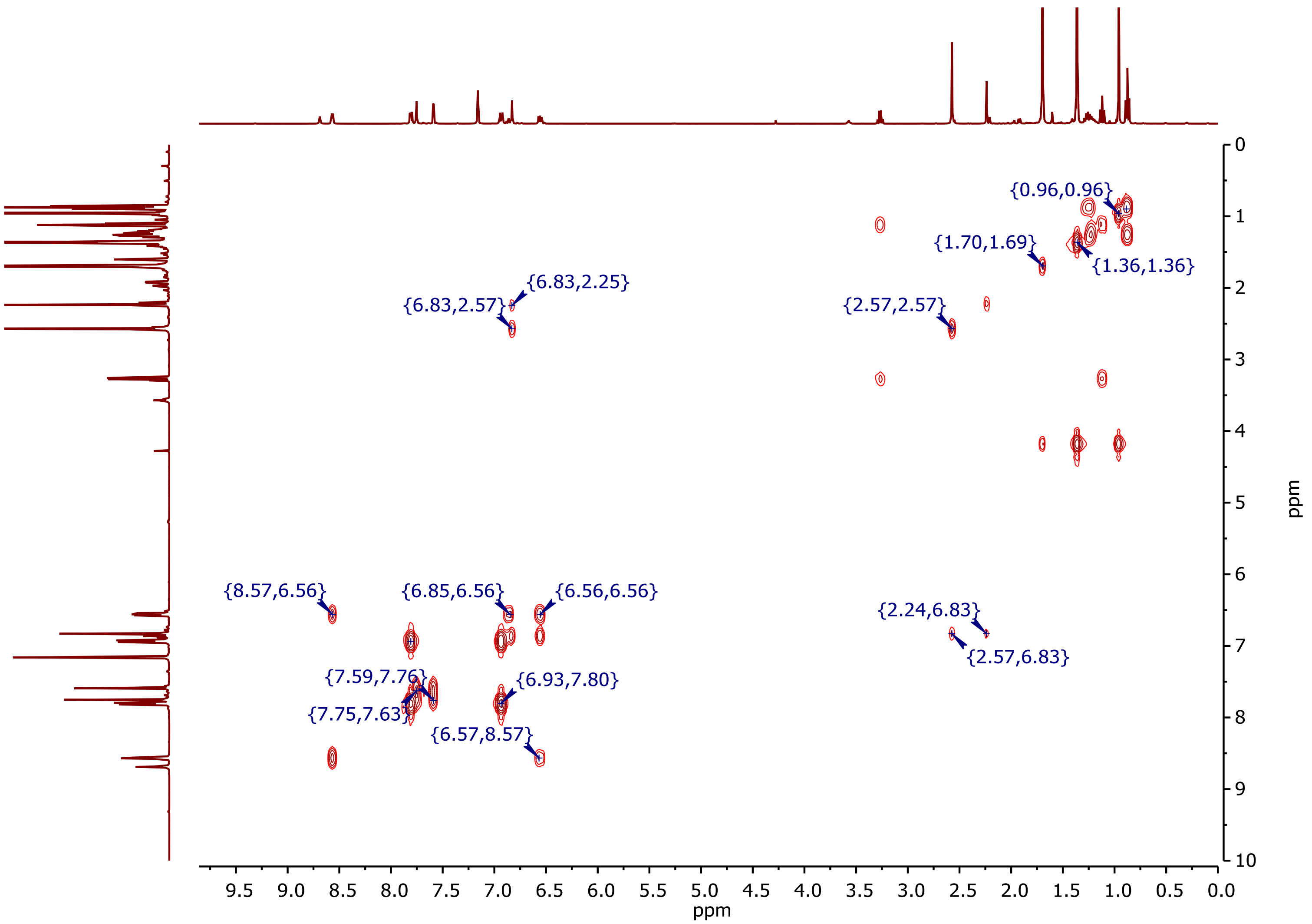


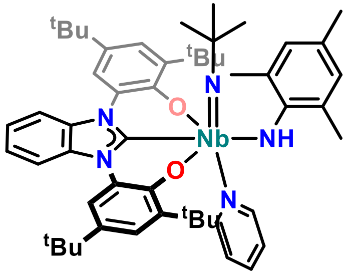


Figure S 29: $^{1}$H-$^{1}$H COSY of **4** in $C_6D_6$ at 298 K.

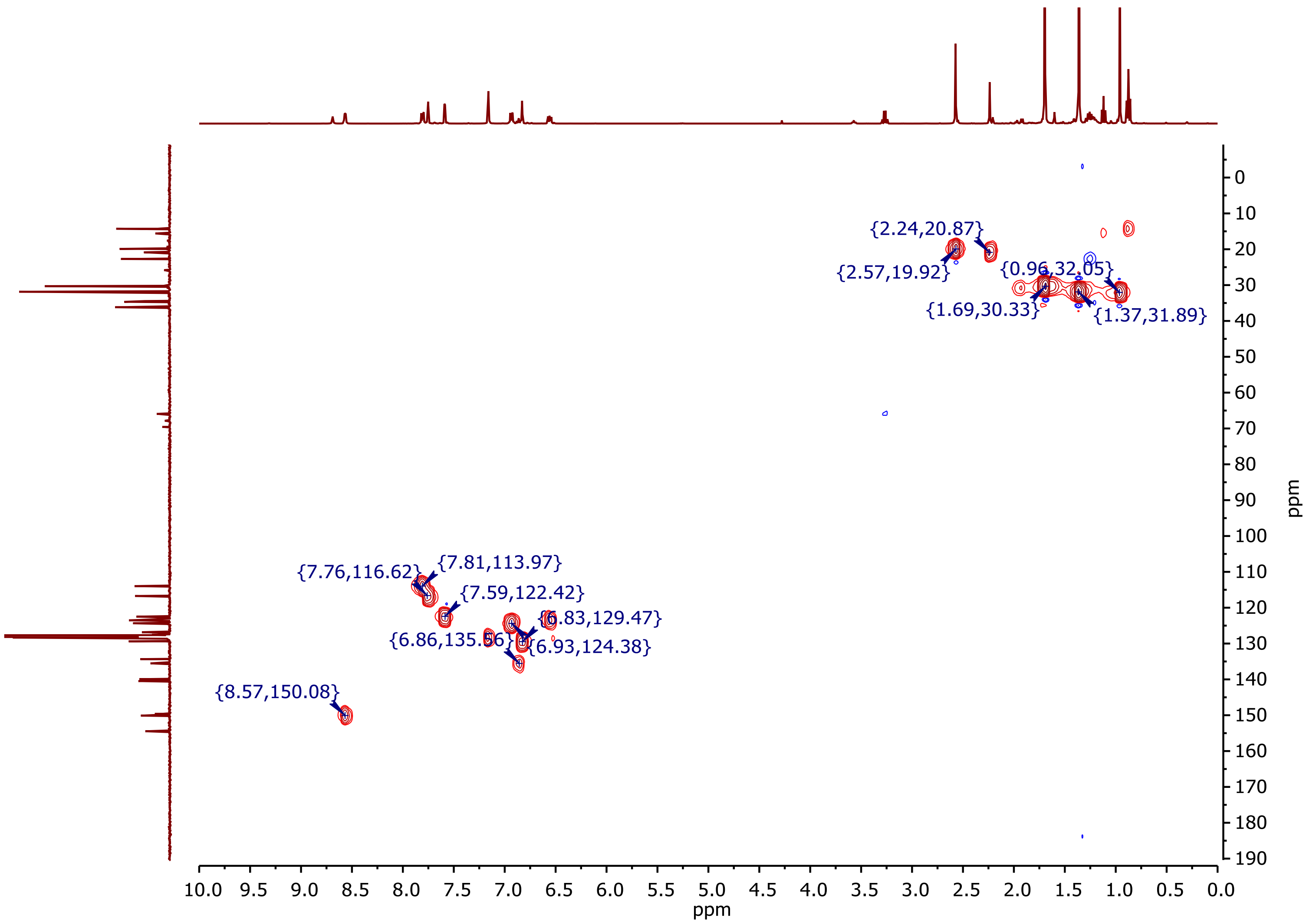


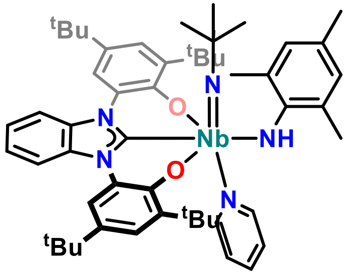


Figure S 30: $^{1}H$-$^{13}C\{^{1}H\}$ HSQC of **4** in $C_6D_6$ at 298 K.

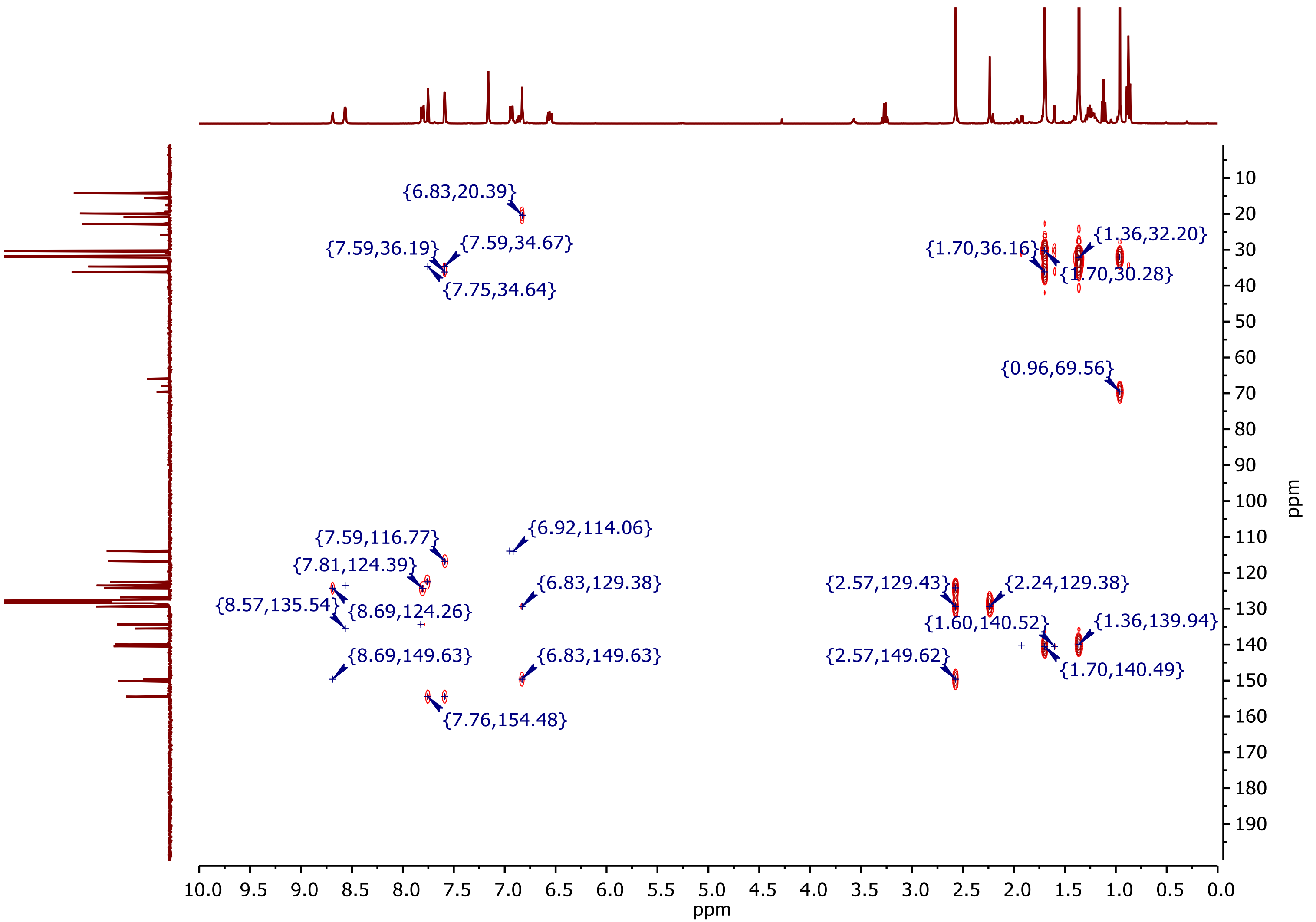


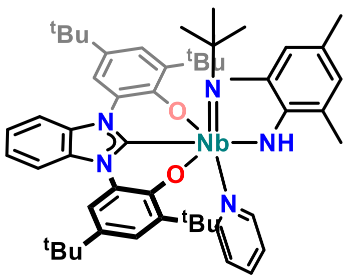


Figure S 31: $^{1}H$-$^{13}C\{^{1}H\}$ HMBC of **4** in $C_6D_6$ at 298K.

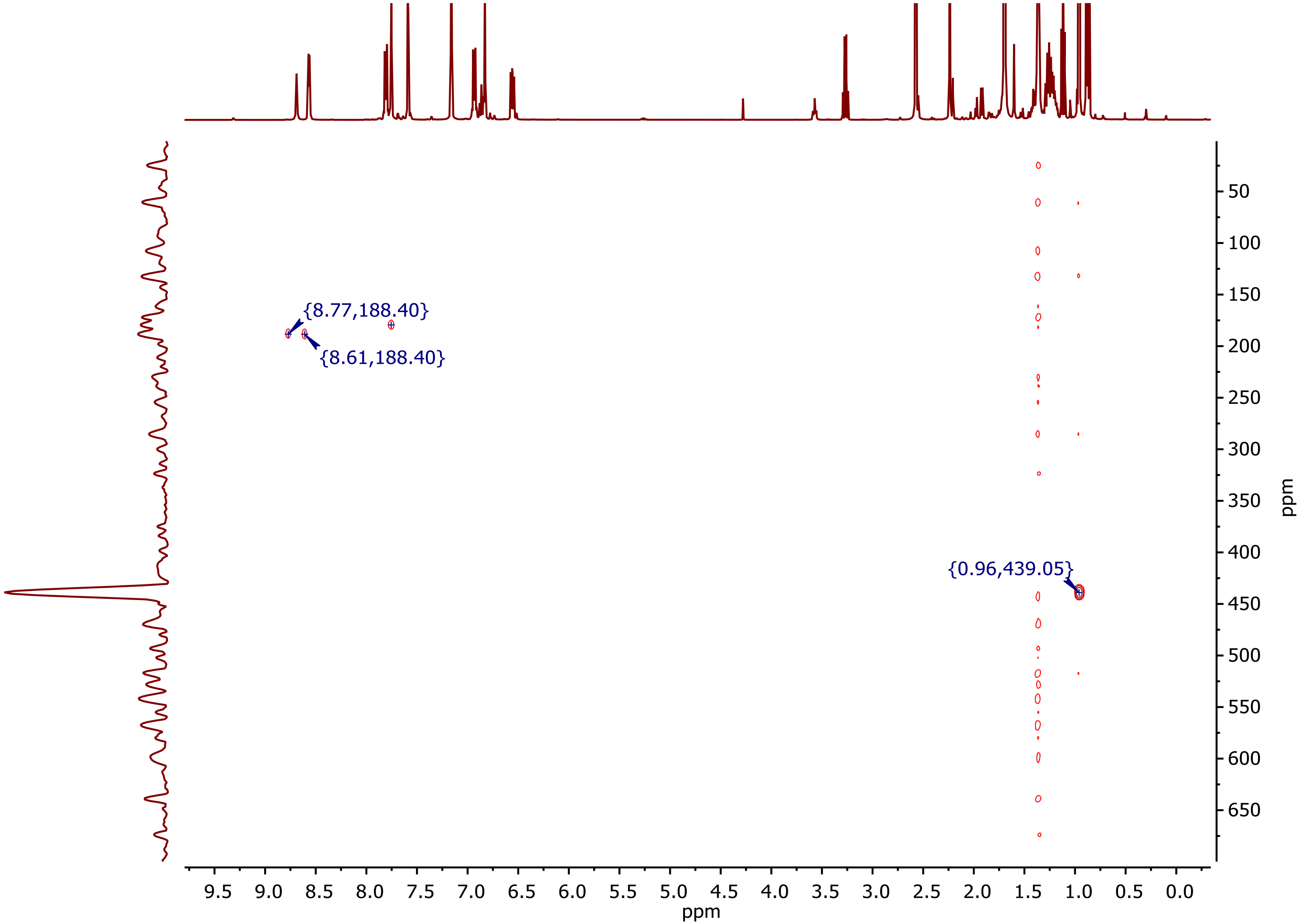


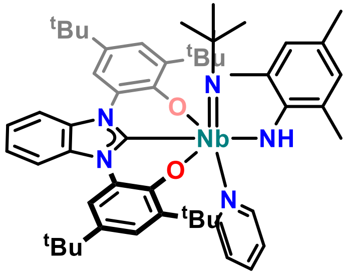


Figure S 32: $^{1}H$-$^{15}N$ HMBC of **4** in $C_6D_6$ at 298K. (41 MHz)

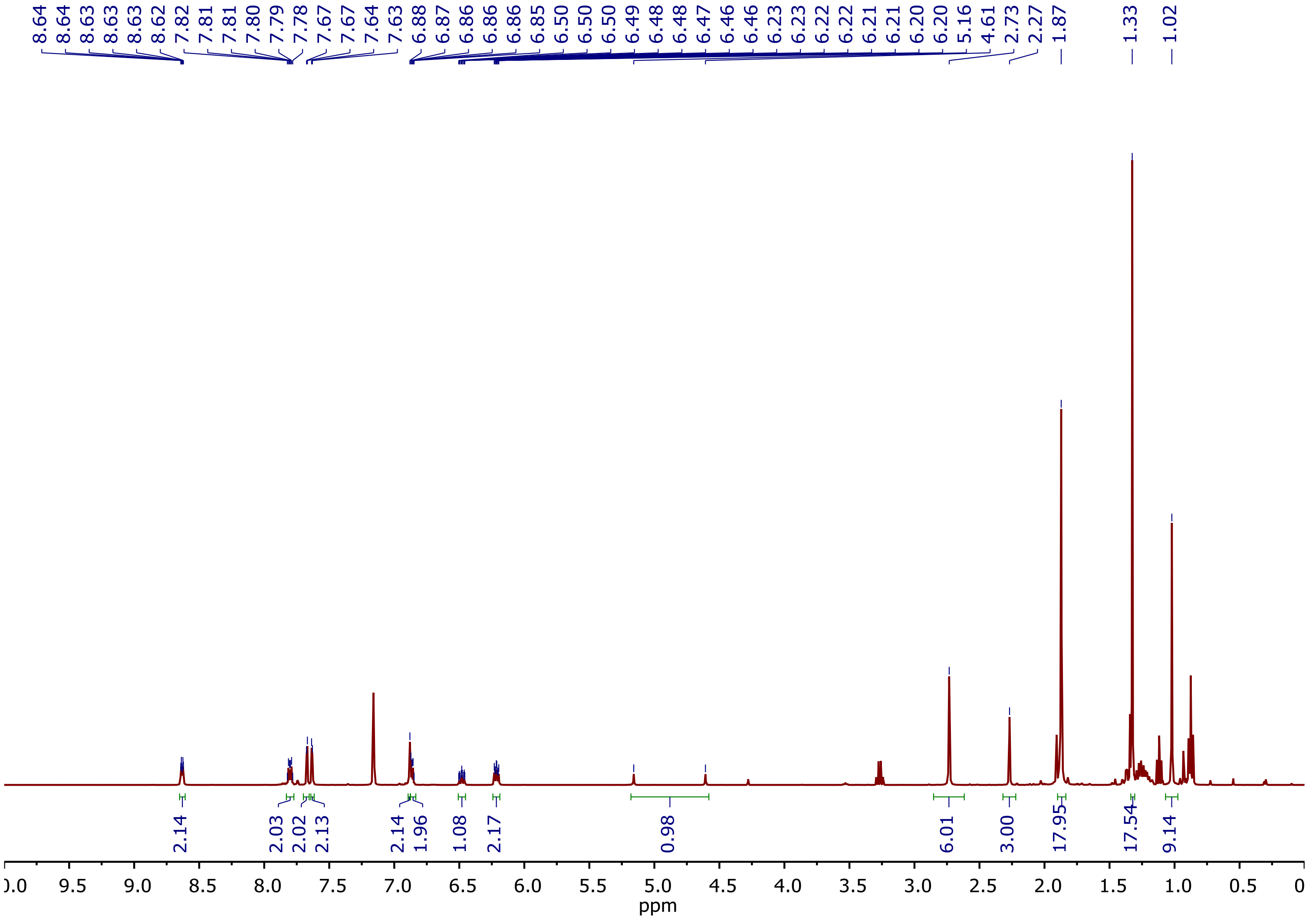

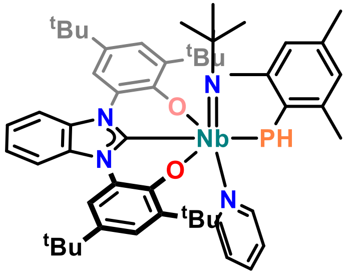

Figure S 33: $^{1}H$ NMR of **5** in $C_6D_6$ at 298 K. (400 MHz)

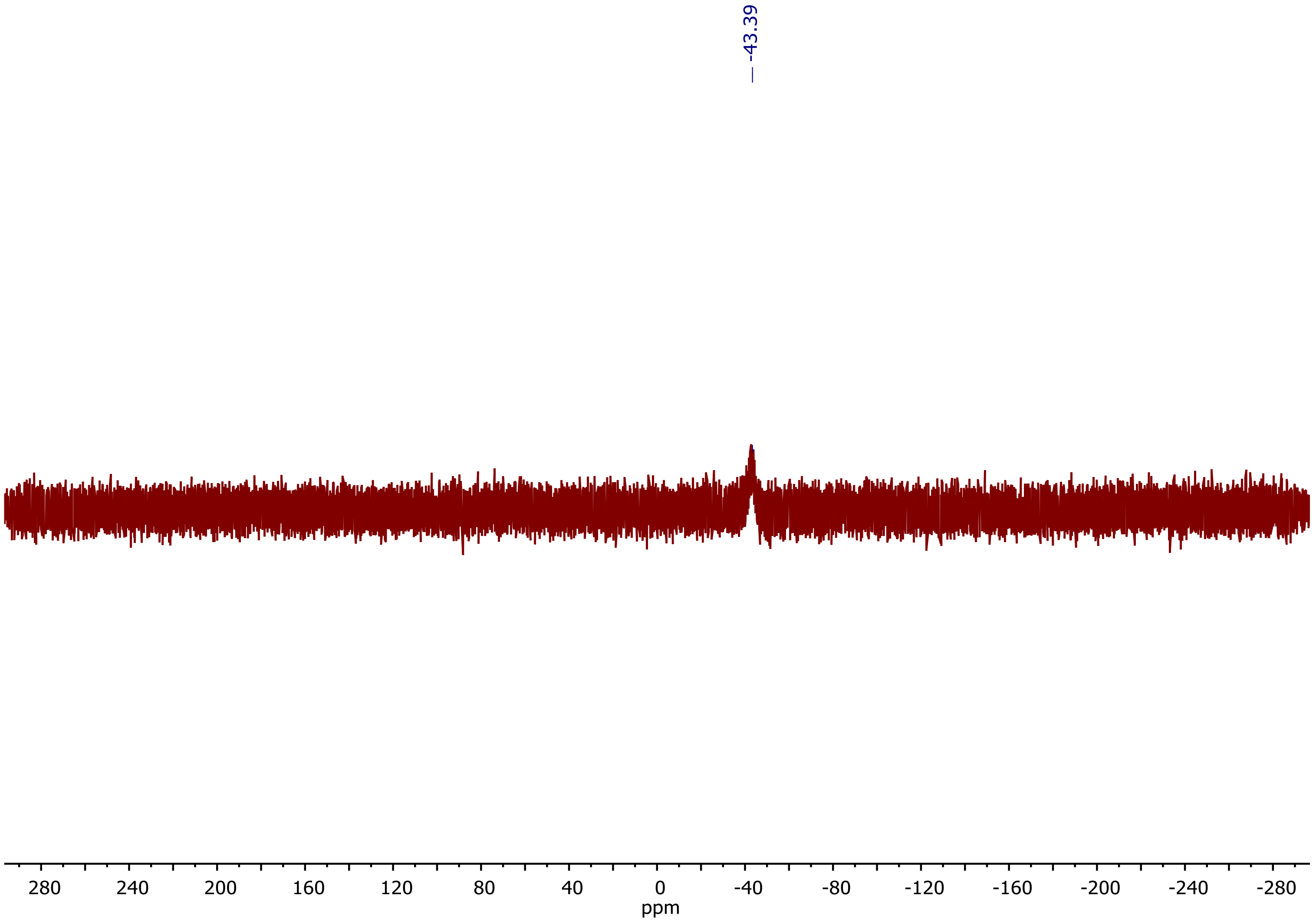


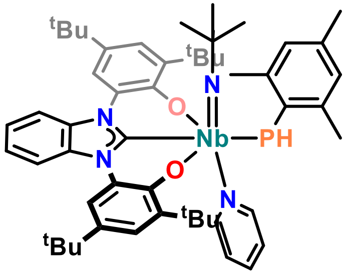


Figure S 34: $^{31}P\{^{1}H\}$ NMR of **5** in $C_6D_6$ at 298 K. (162 MHz)

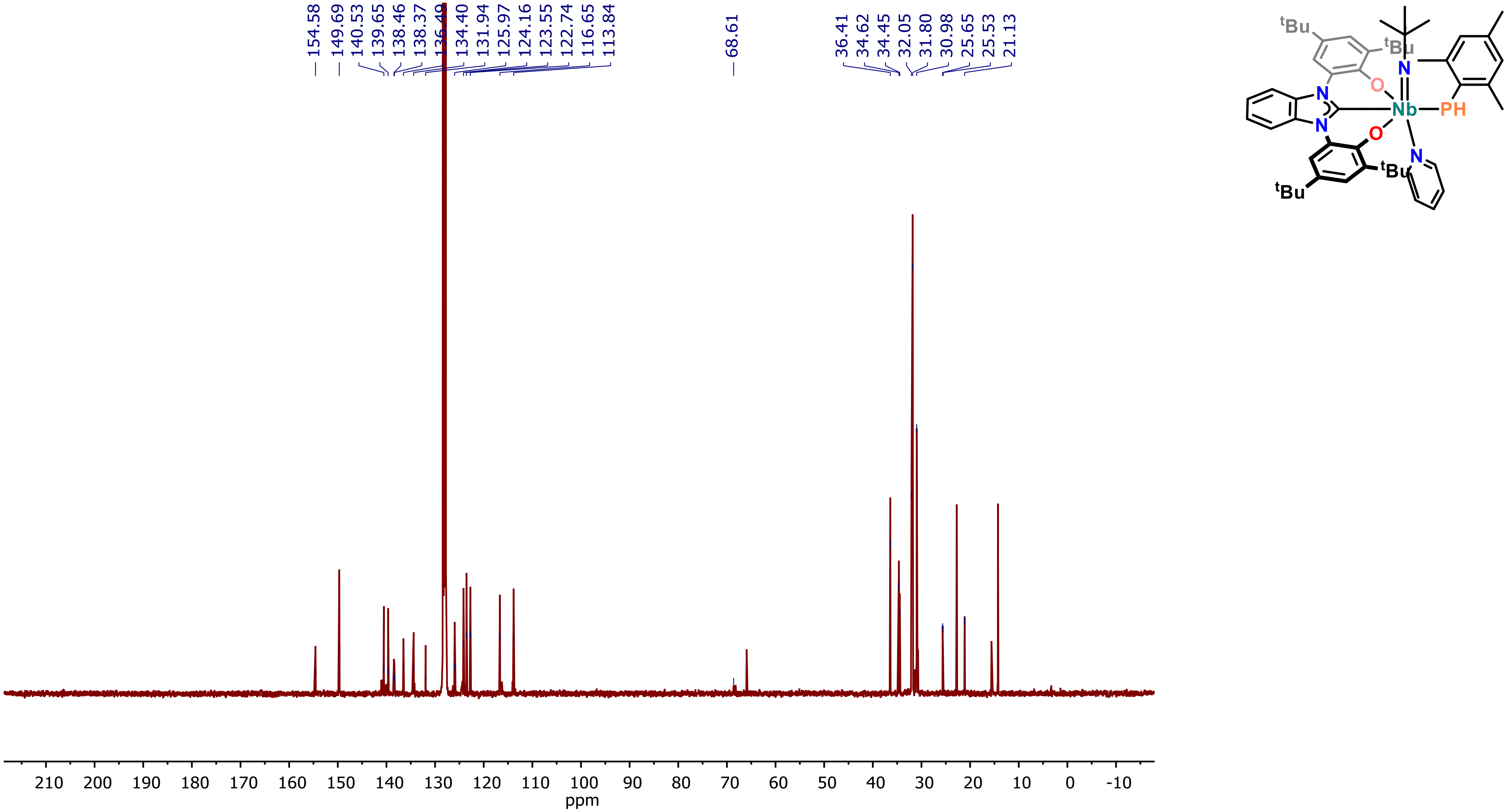


Figure S 35: $^{13}C\{^{1}H\}$ NMR of **5** in $C_6D_6$ at 298 K. (101 MHz)

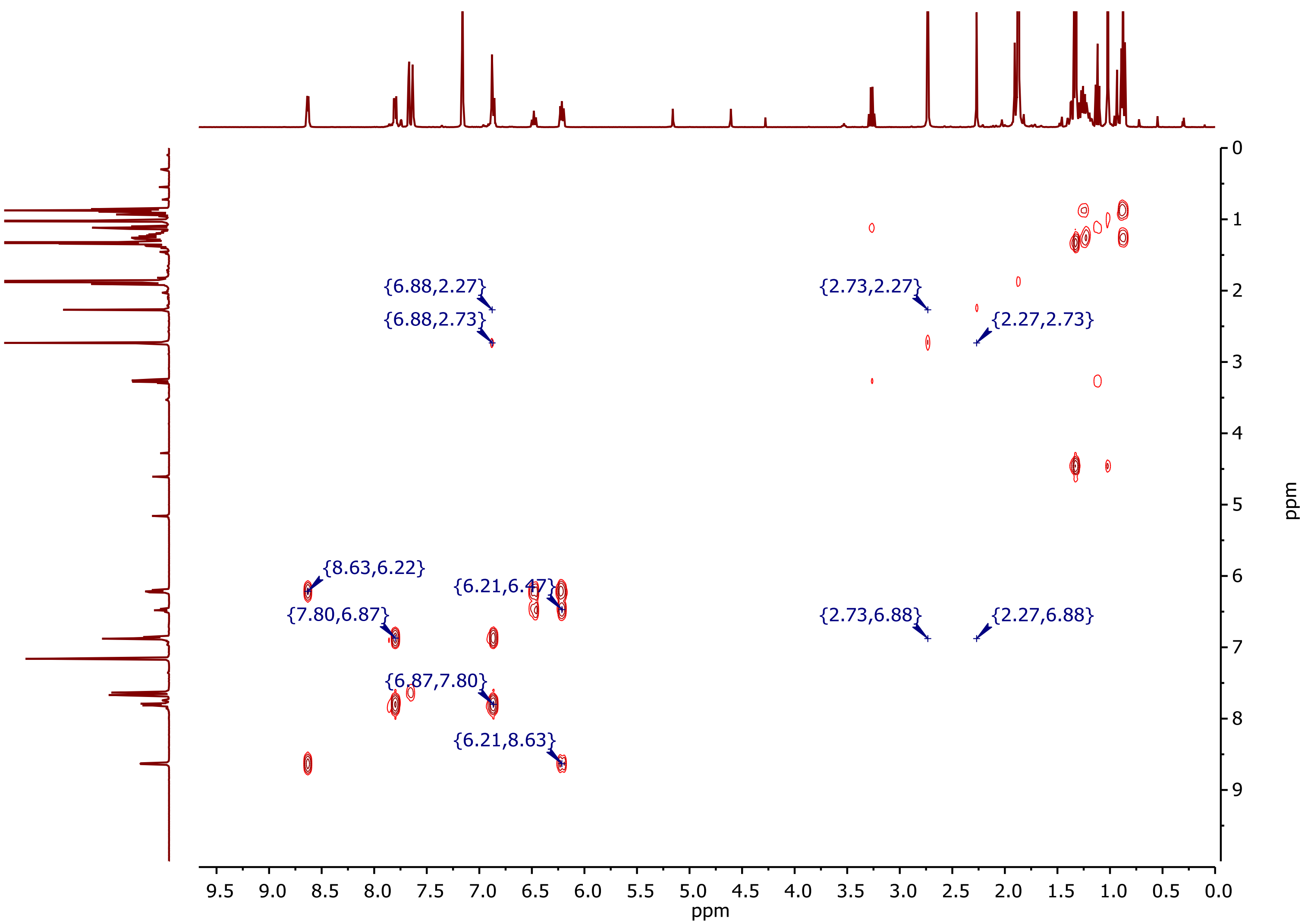


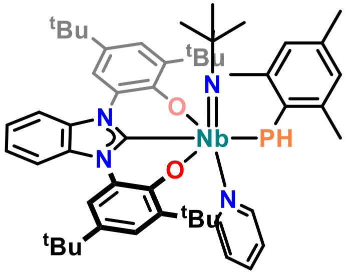


Figure S 36: $^{1}$H-$^{1}$H COSY of **5** in $C_6D_6$ at 298 K.

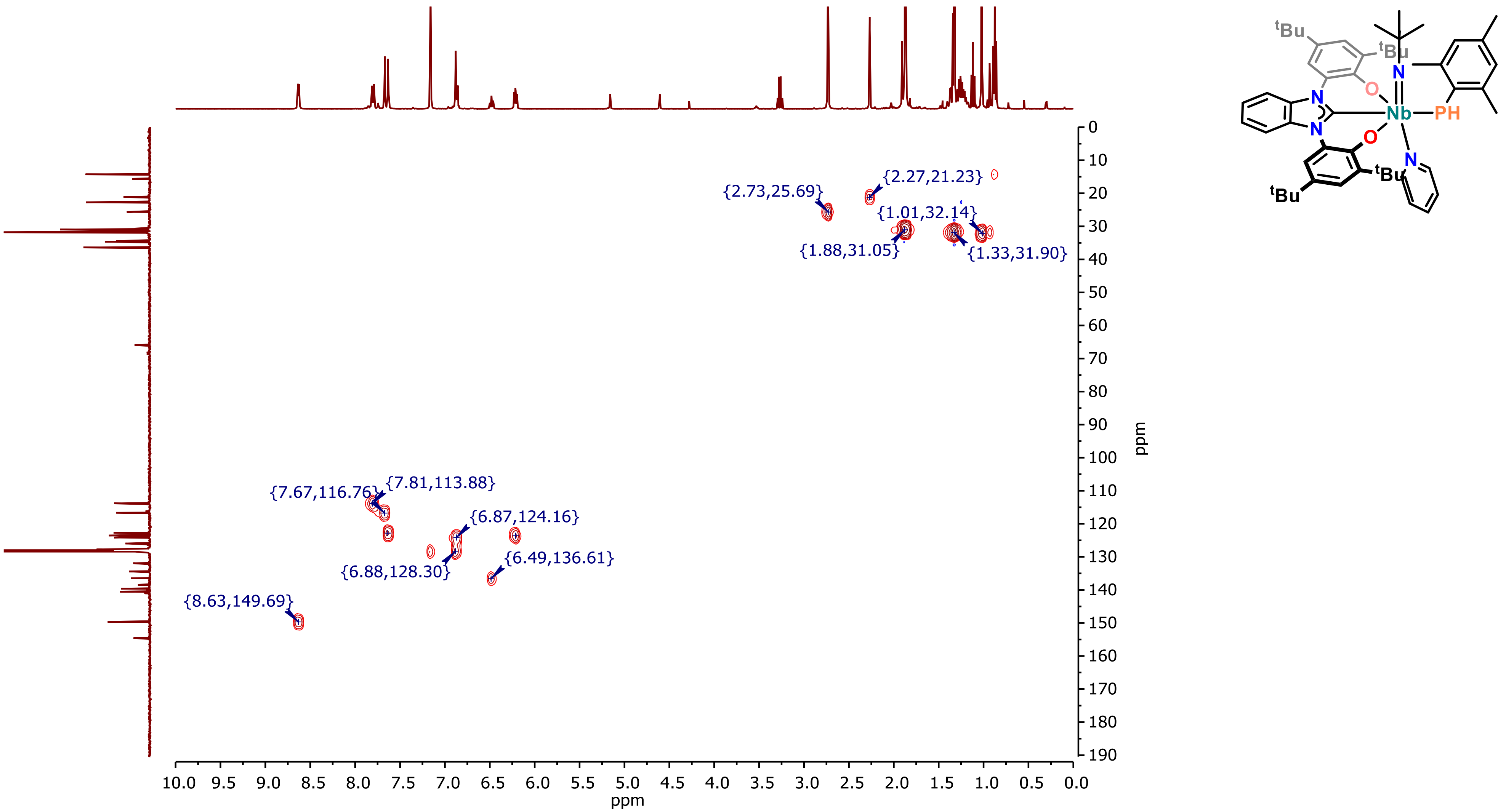


Figure S 37: $^{1}H$-$^{13}C\{^{1}H\}$ HSQC of **5** in $C_6D_6$ at 298 K.

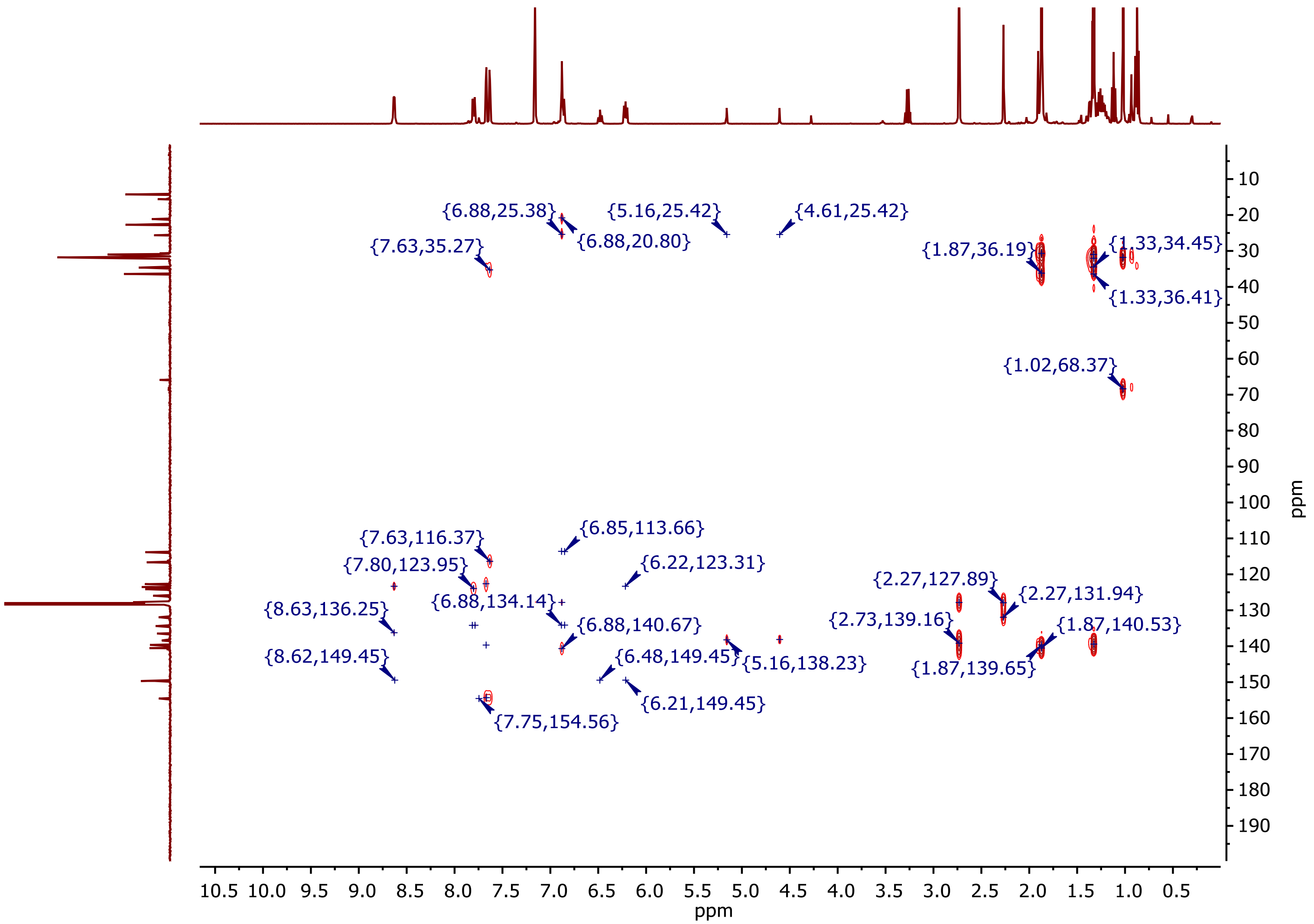


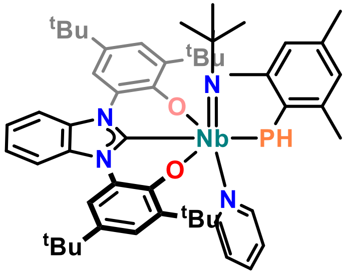


Figure S 38: $^{1}H$-$^{13}C\{^{1}H\}$ HMBC of **5** in $C_6D_6$ at 298K.

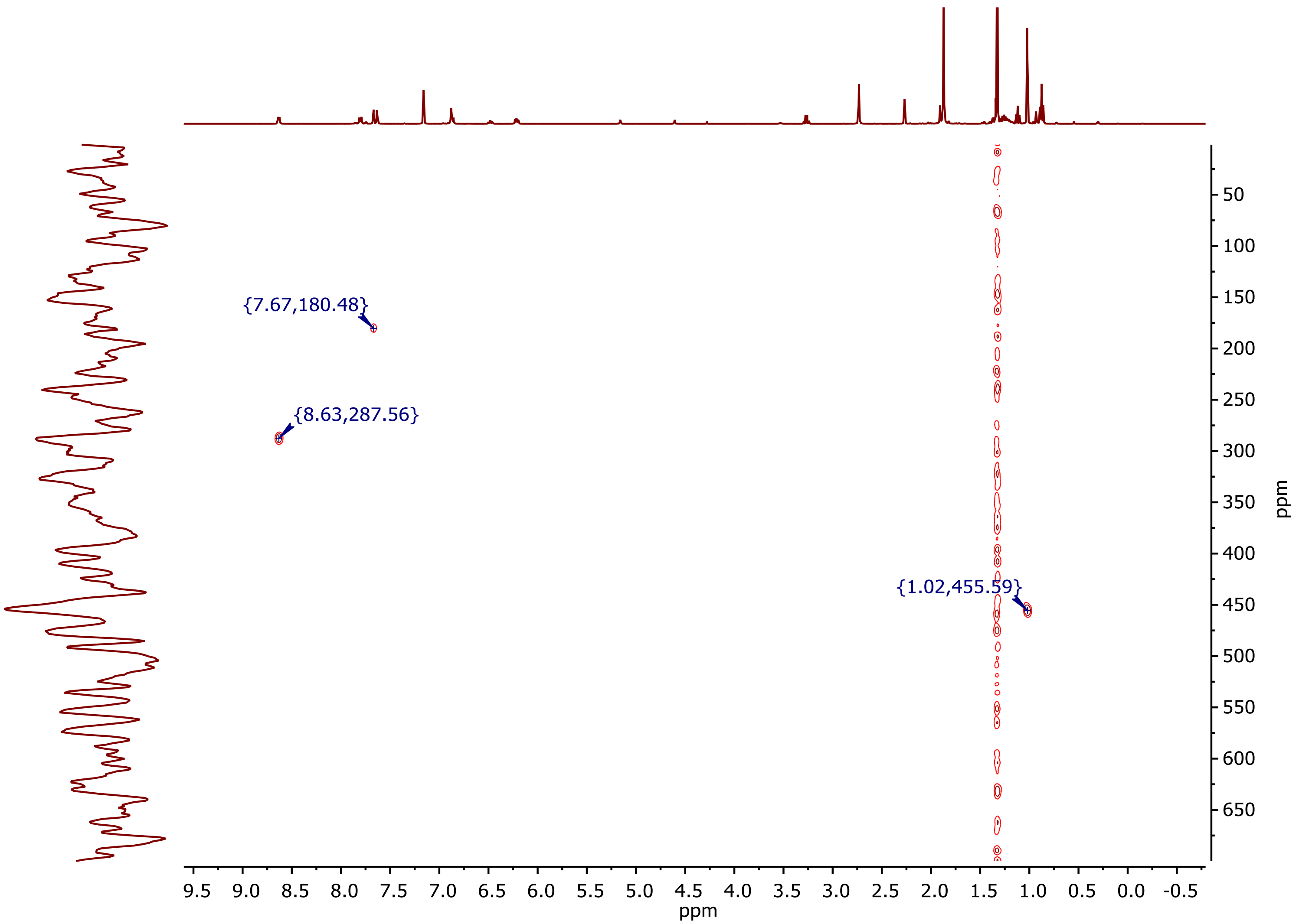


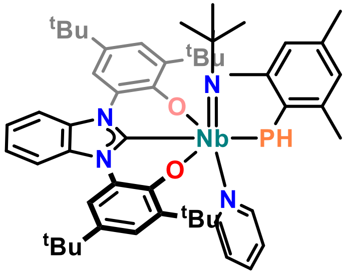


Figure S 39: $^{1}H$-$^{15}N$ HMBC of **5** in $C_6D_6$ at 298K. (41 MHz)

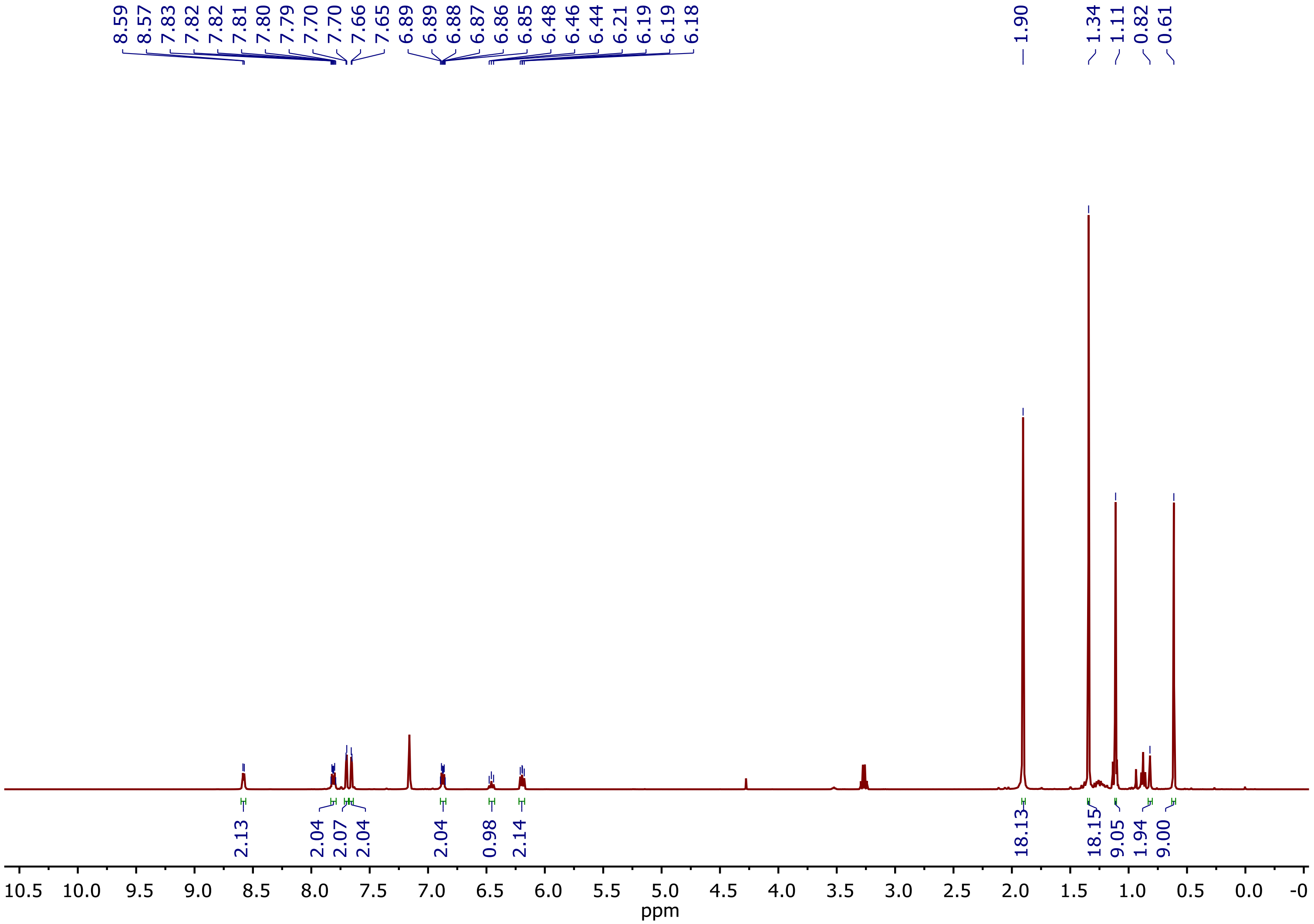


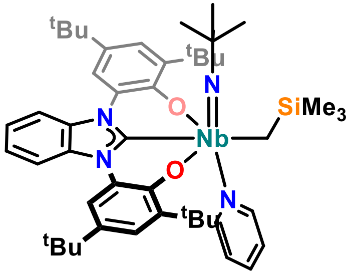


Figure S 40: $^{1}$H NMR of **6** in $C_6D_6$ at 298K. (400 MHz)

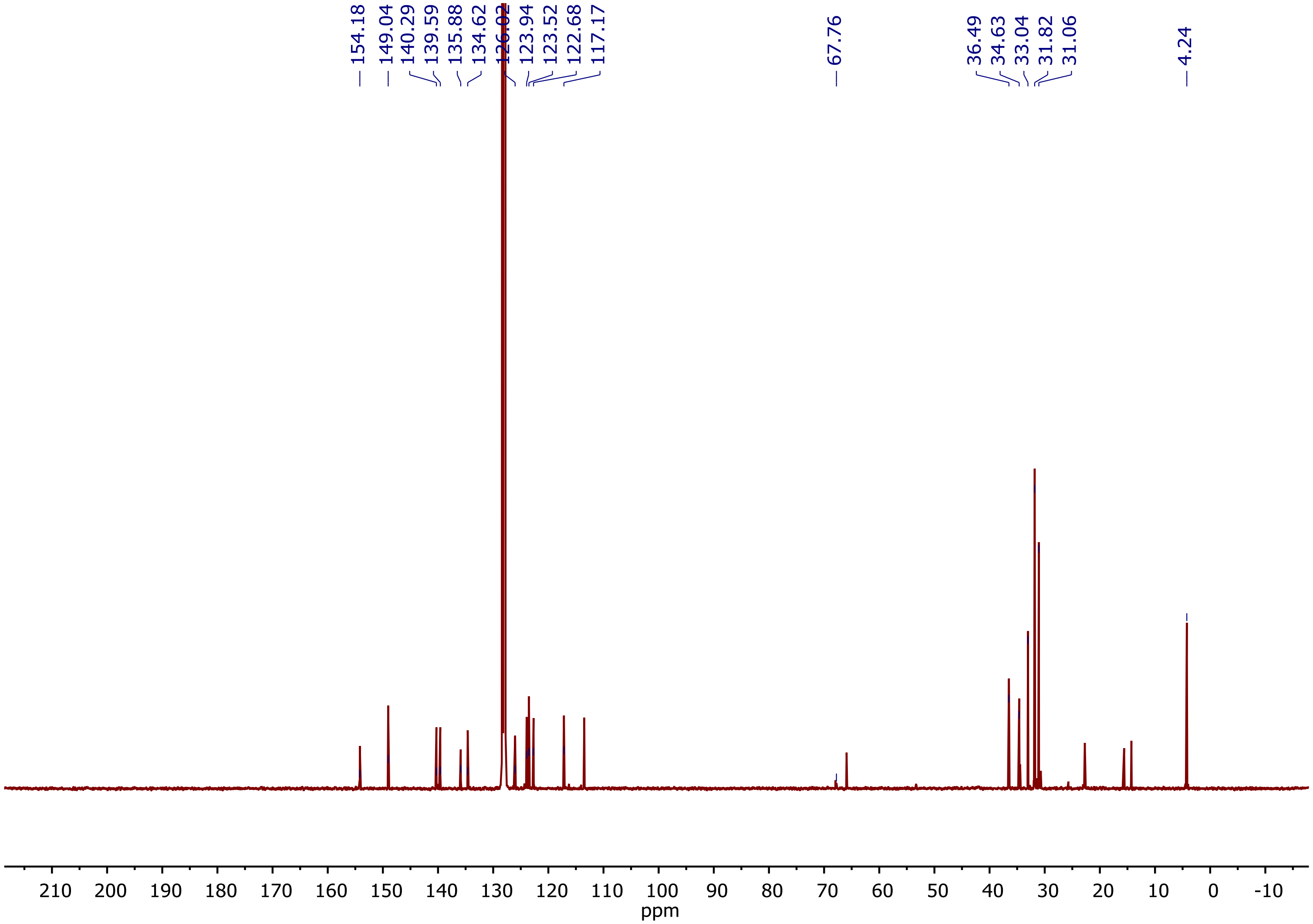


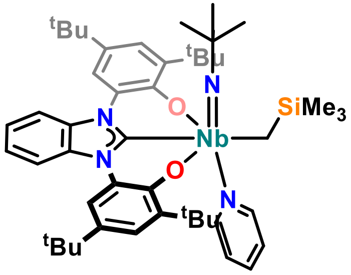


Figure S 41: $^{13}C\{^1H\}$ NMR of **6** in $C_6D_6$ at 298K. (101 MHz)

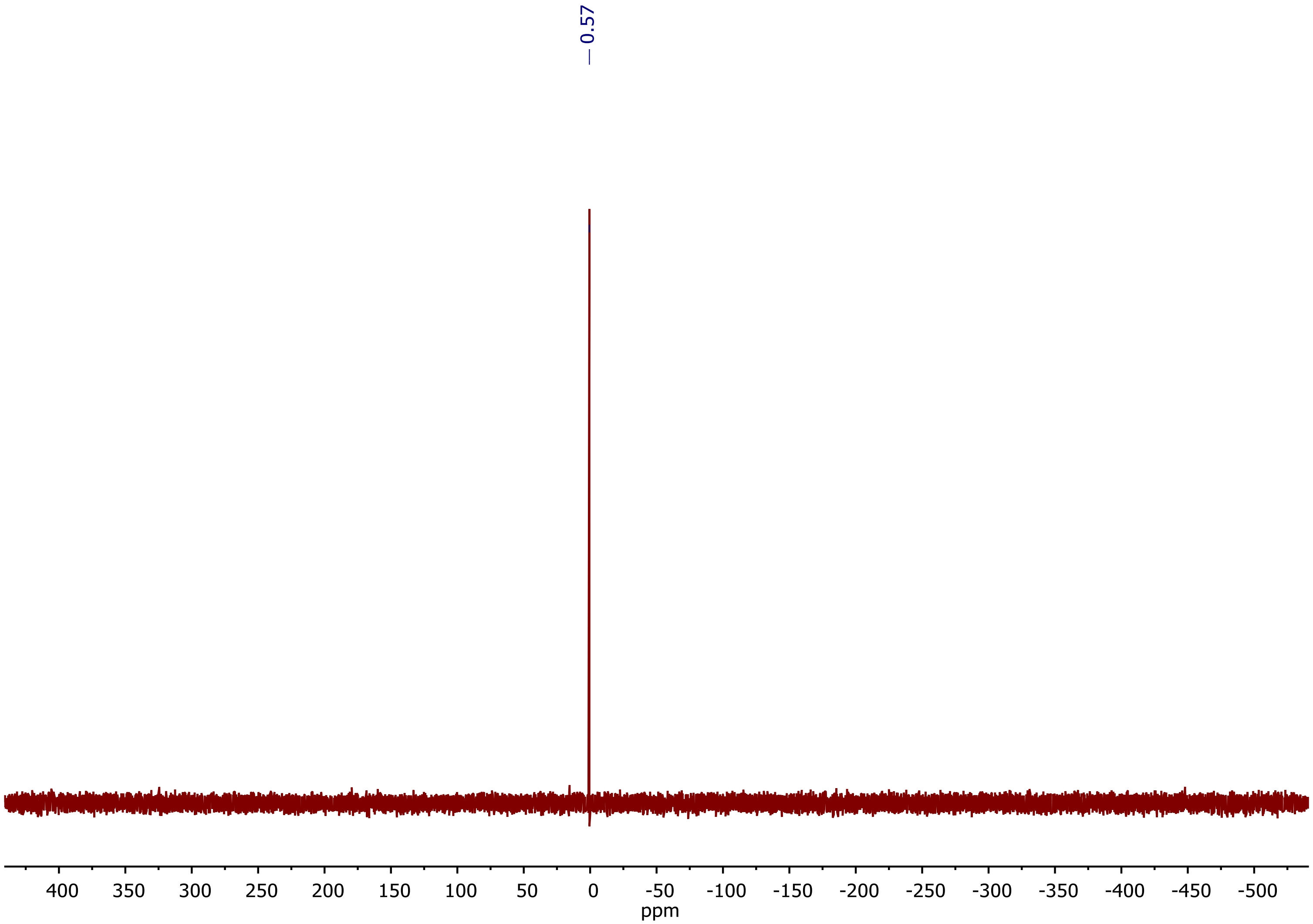


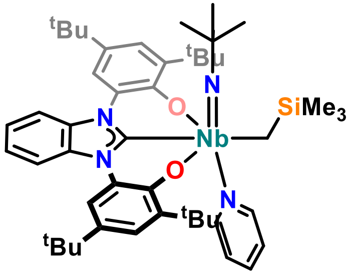


Figure S 42: $^{29}$Si NMR of **6** in $C_6D_6$ at 298K. (80 MHz)

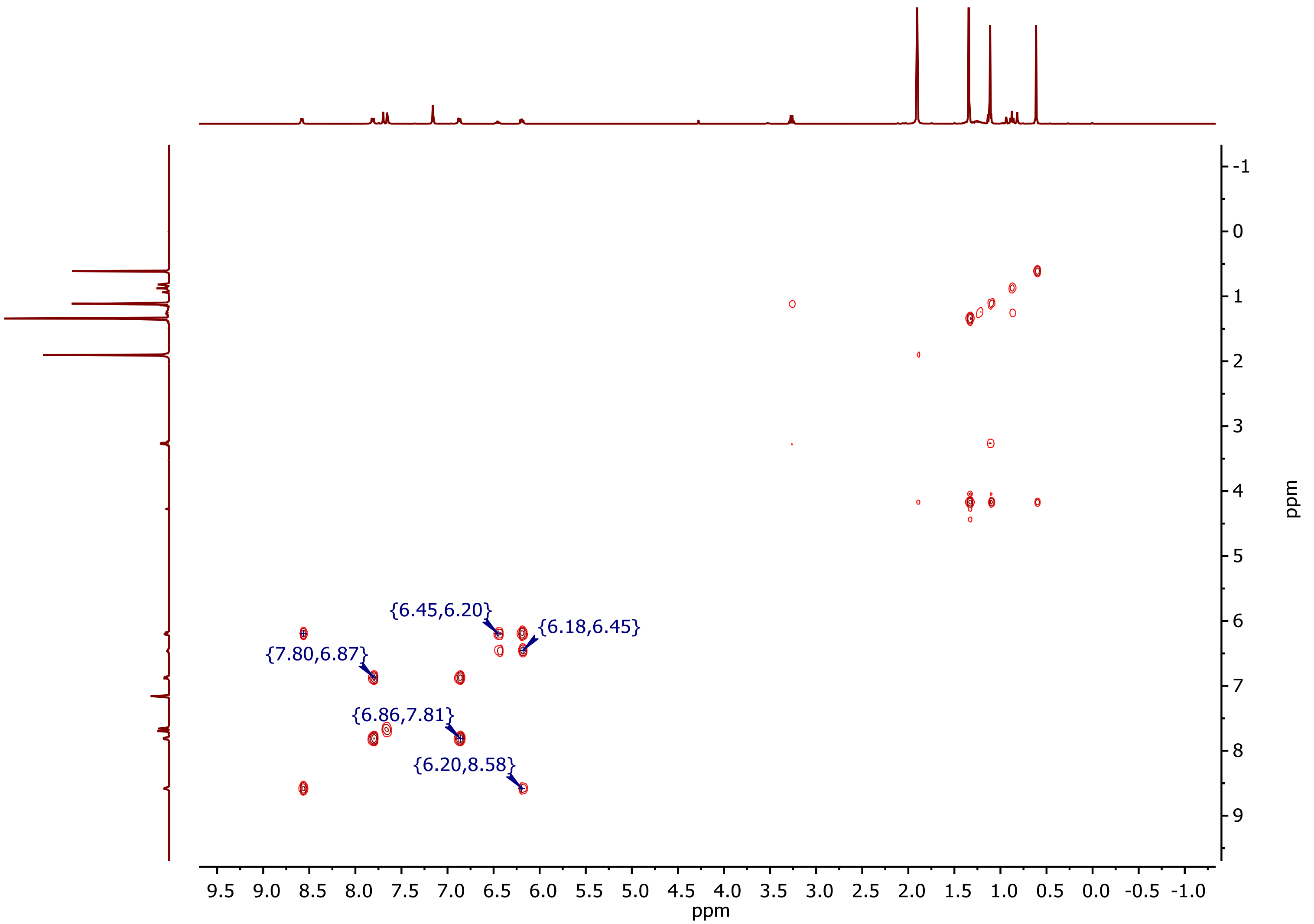


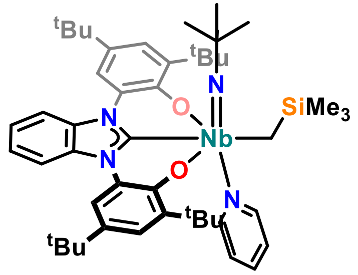


Figure S 43: $^{1}H$-$^{1}H$ COSY of **6** in $C_6D_6$ at 298 K.

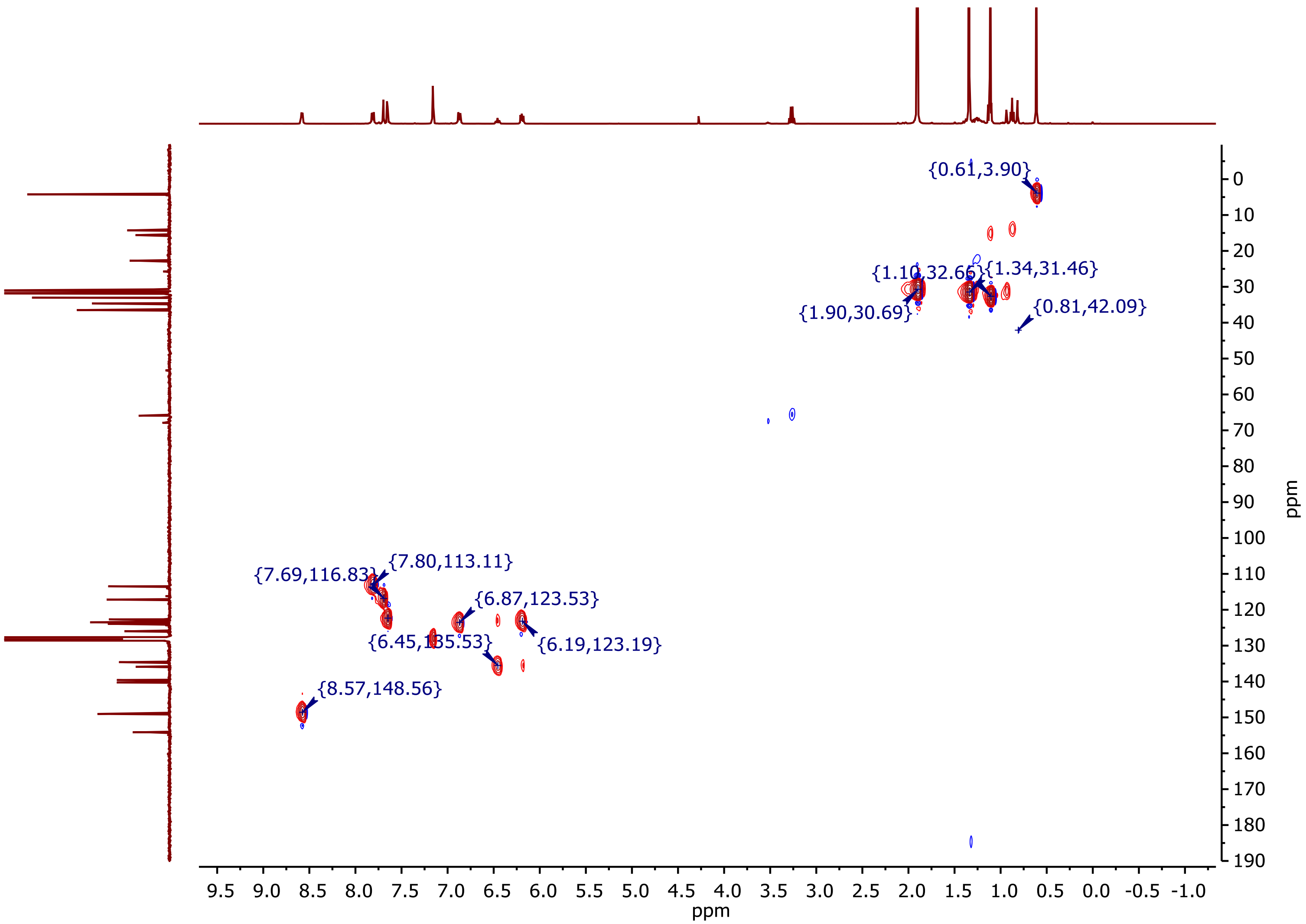


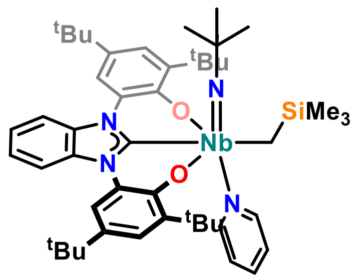


Figure S 44: $^{1}H$-$^{13}C\{^{1}H\}$ HSQC of **6** in $C_6D_6$ at 298 K.

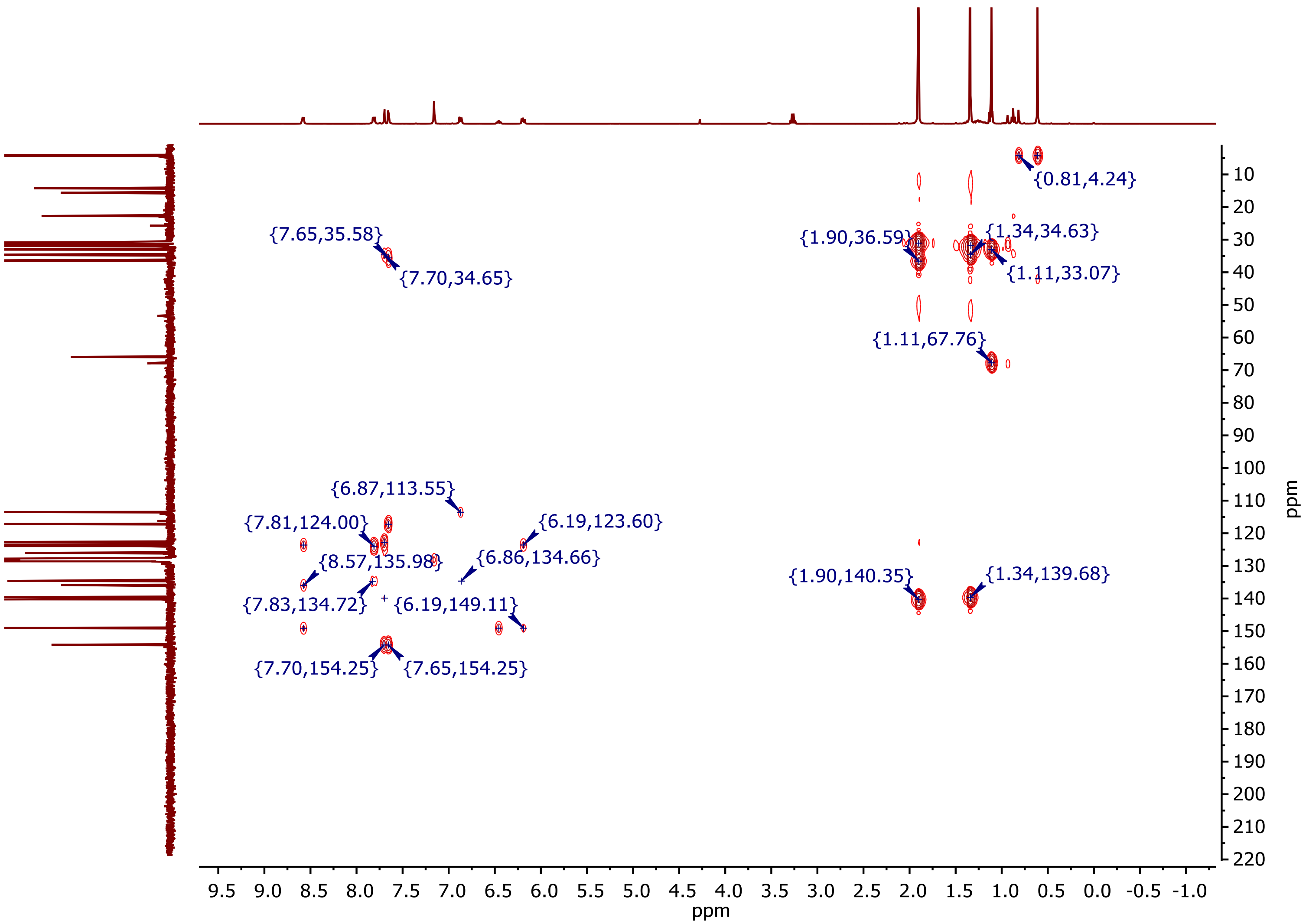


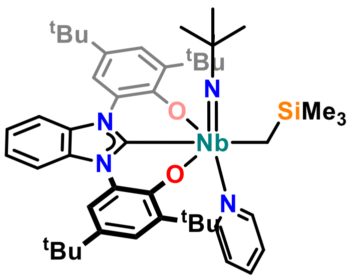


Figure S 45: $^{1}H$-$^{13}C\{^{1}H\}$ HMBC of **6** in $C_6D_6$ at 298 K.

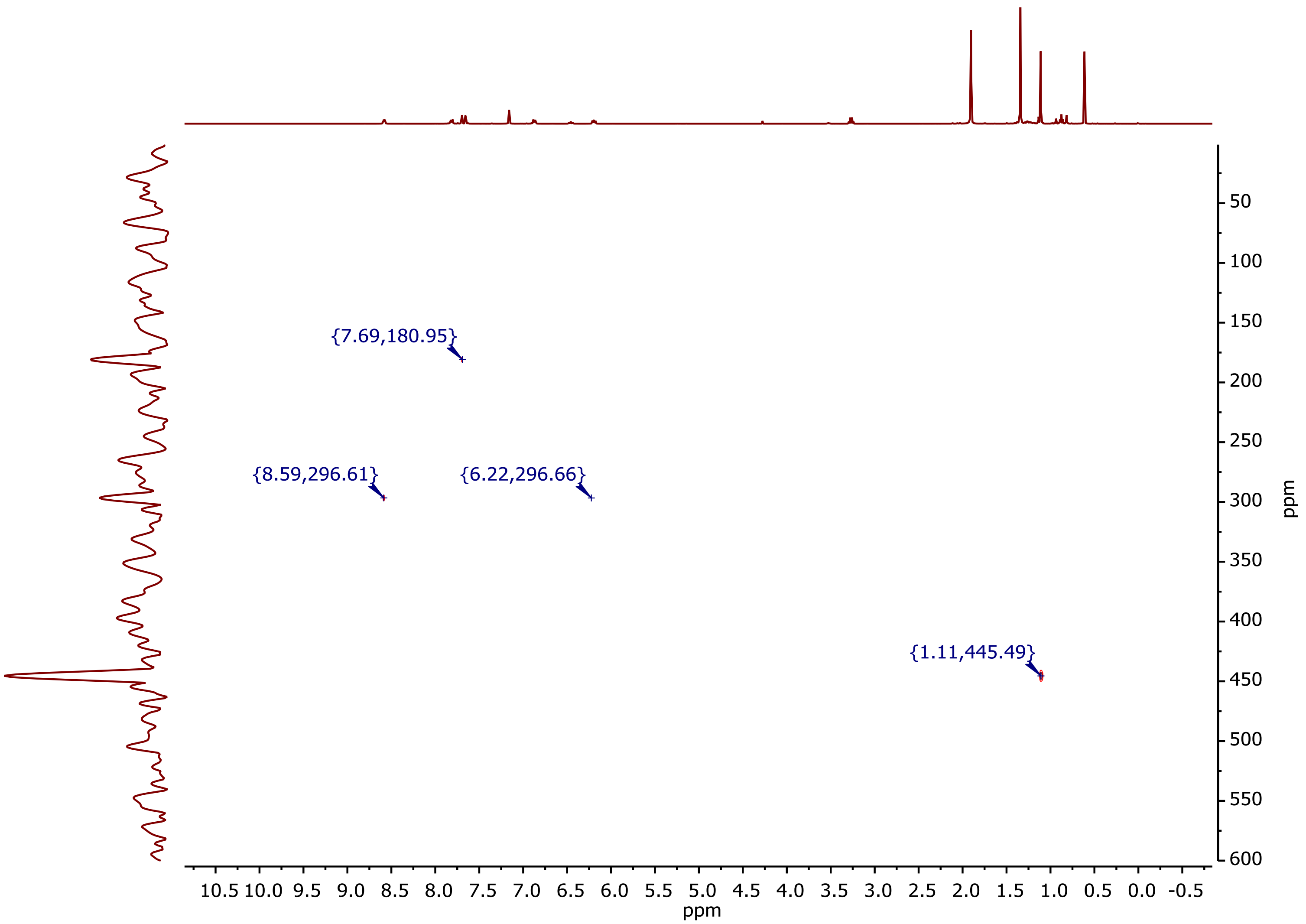


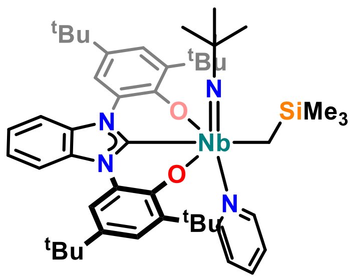


Figure S 46: $^{1}H$-$^{15}N$ HMBC of **6** in $C_6D_6$ at 298K. (41 MHz)

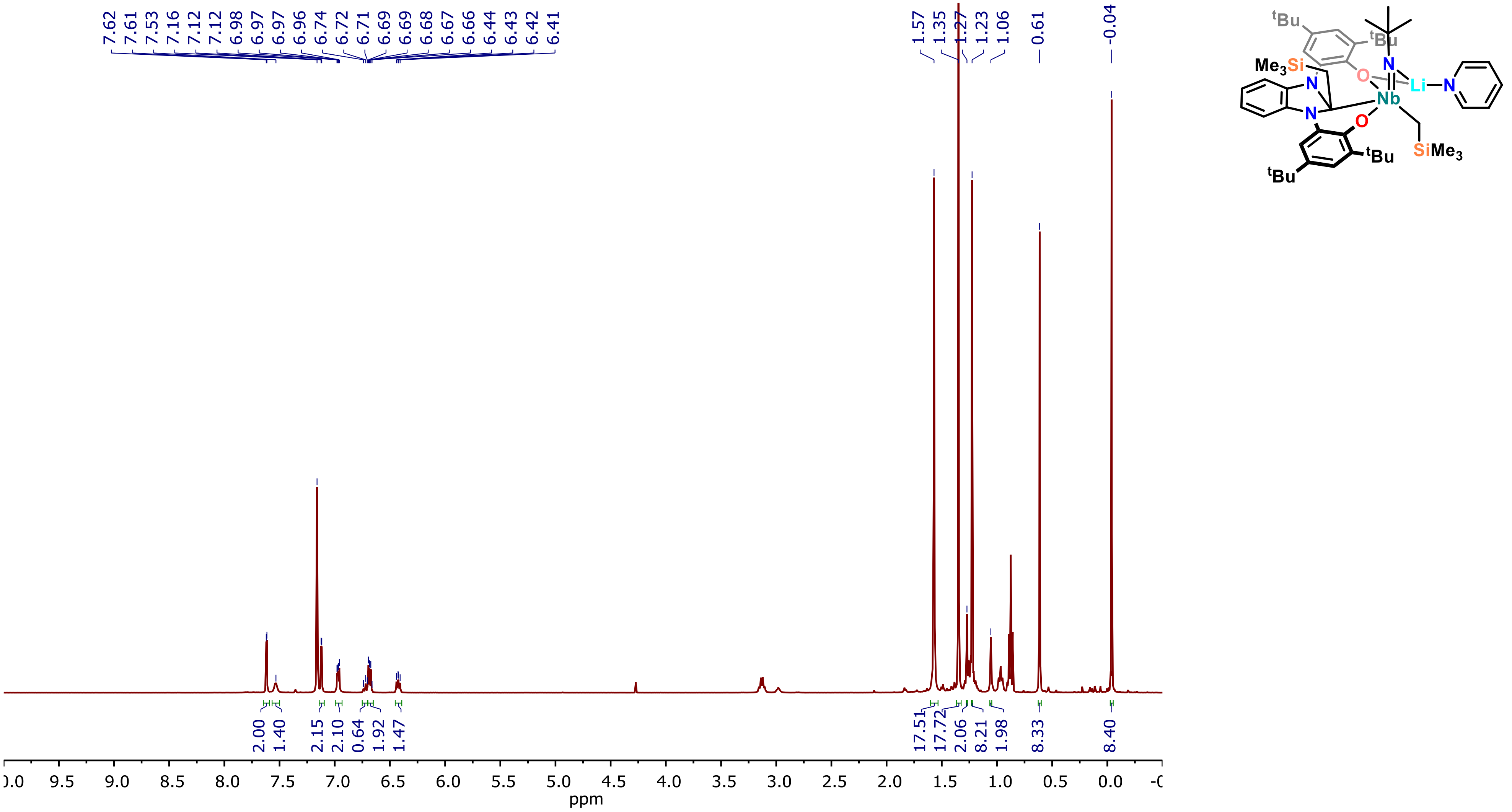


Figure S 47: $^{1}H$ NMR of **7** in $C_6D_6$ at 298 K. (400 MHz)

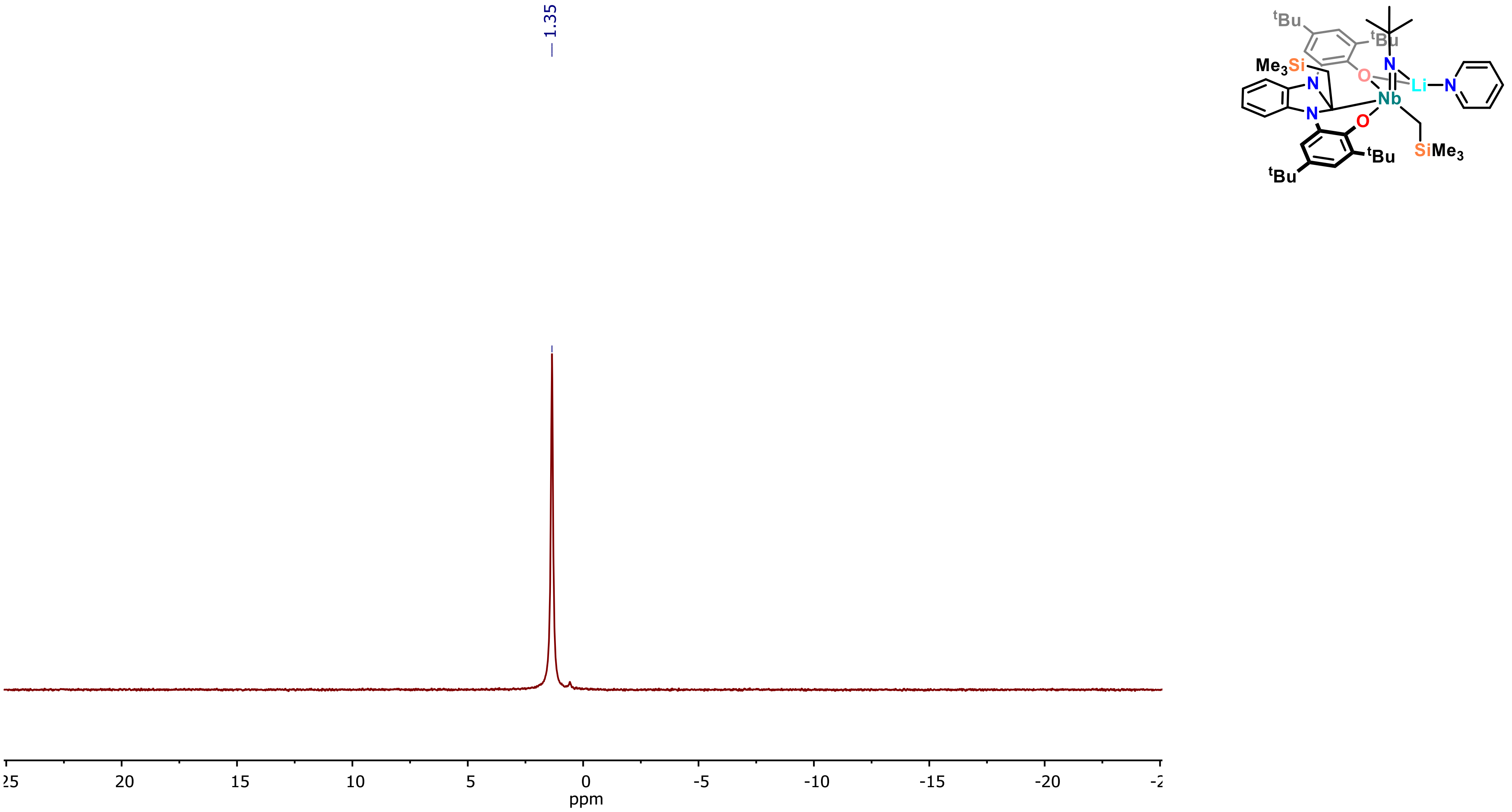


Figure S 48: $^{7}$Li NMR of **7** in $C_6D_6$ at 298 K. (156 MHz)

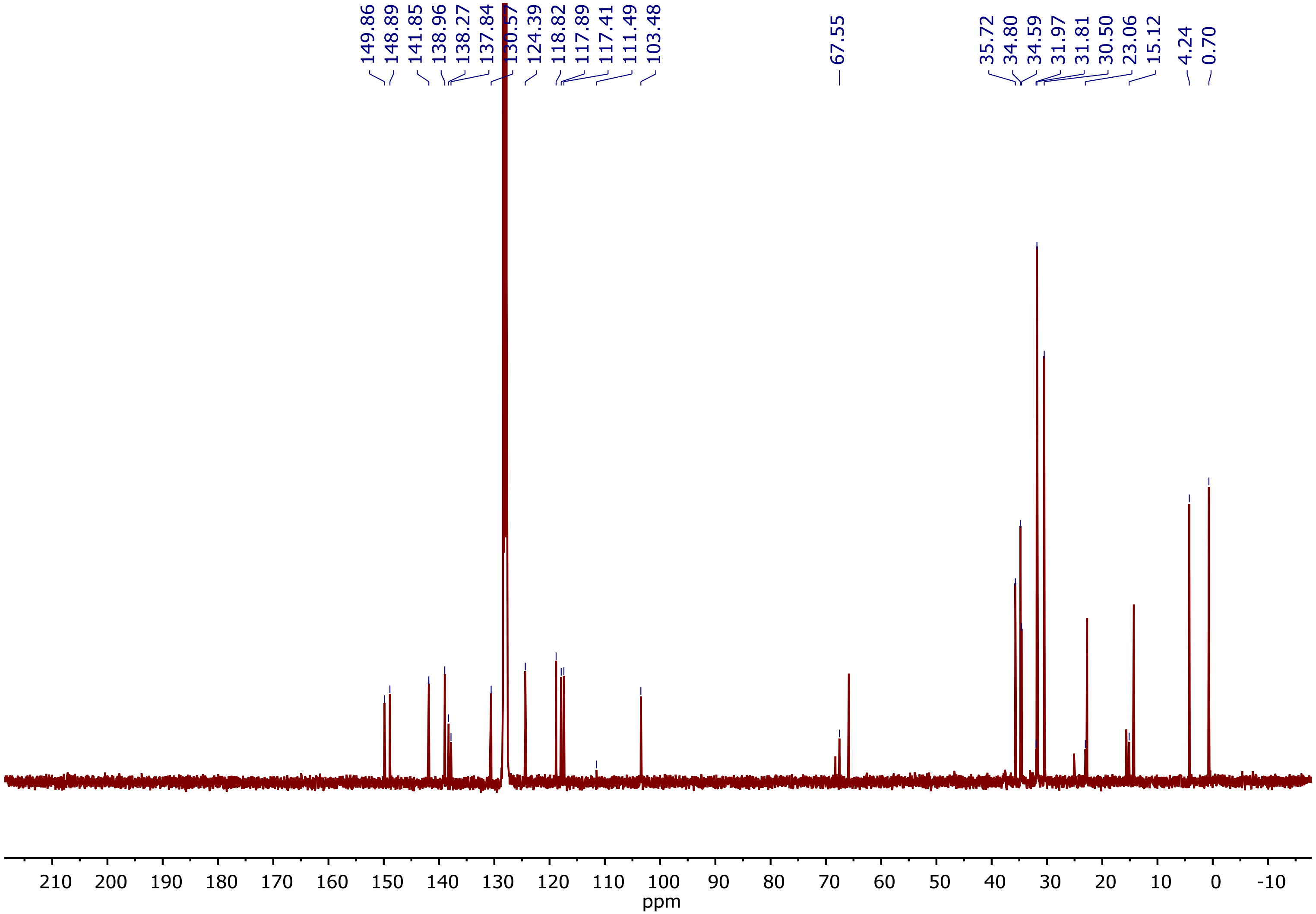


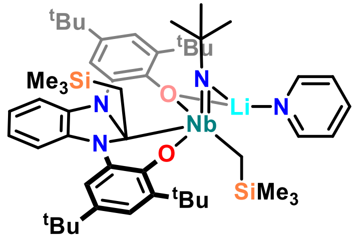


Figure S 49: $^{13}C\{^{1}H\}$ NMR of **7** in $C_6D_6$ at 298 K. (101 MHz)

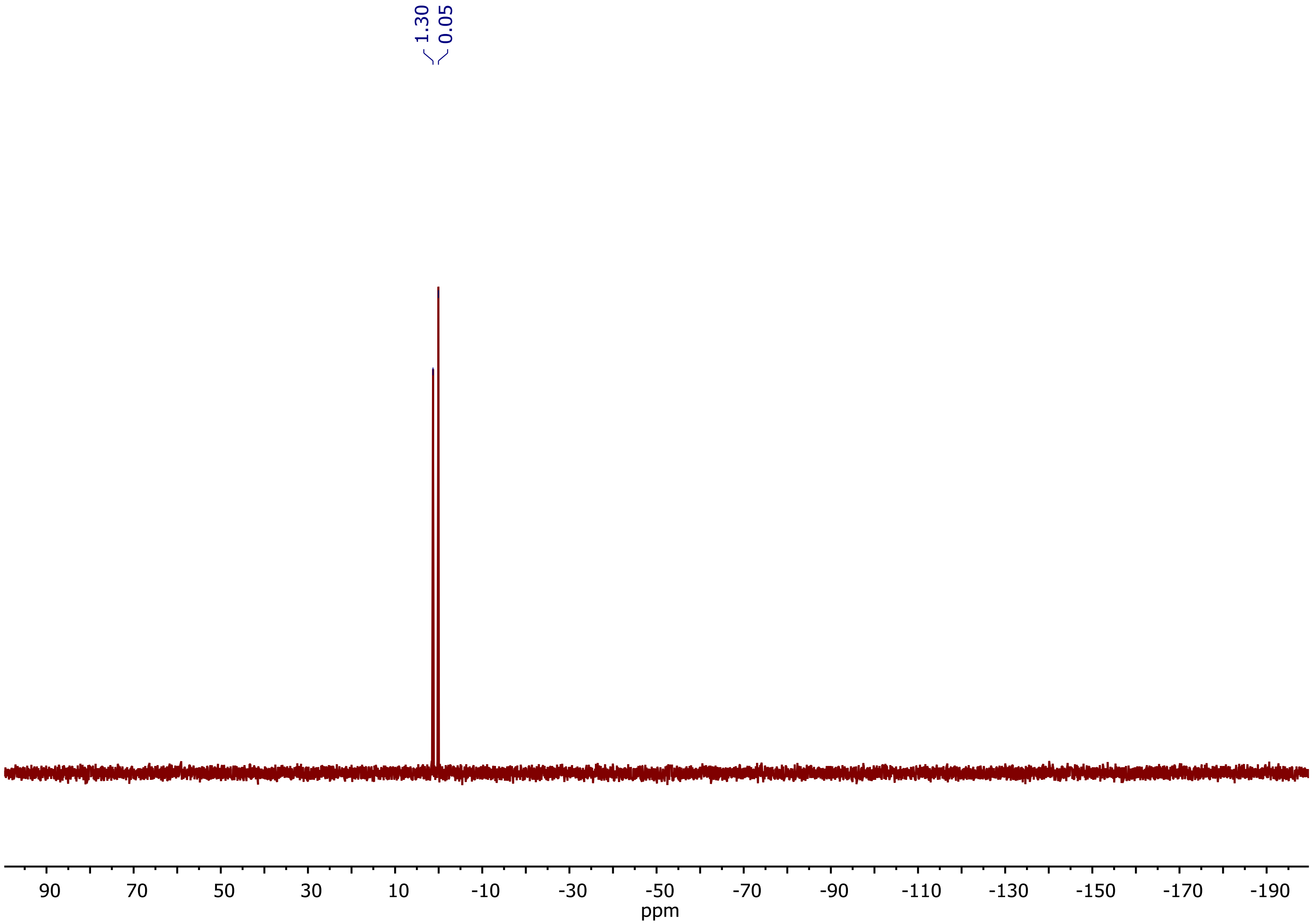


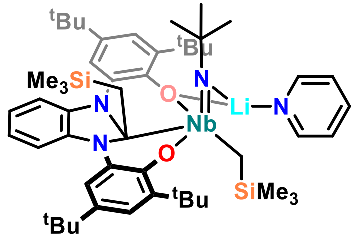


Figure S 50: $^{29}$Si NMR of **7** in $C_6D_6$ at 298 K. (80 MHz)

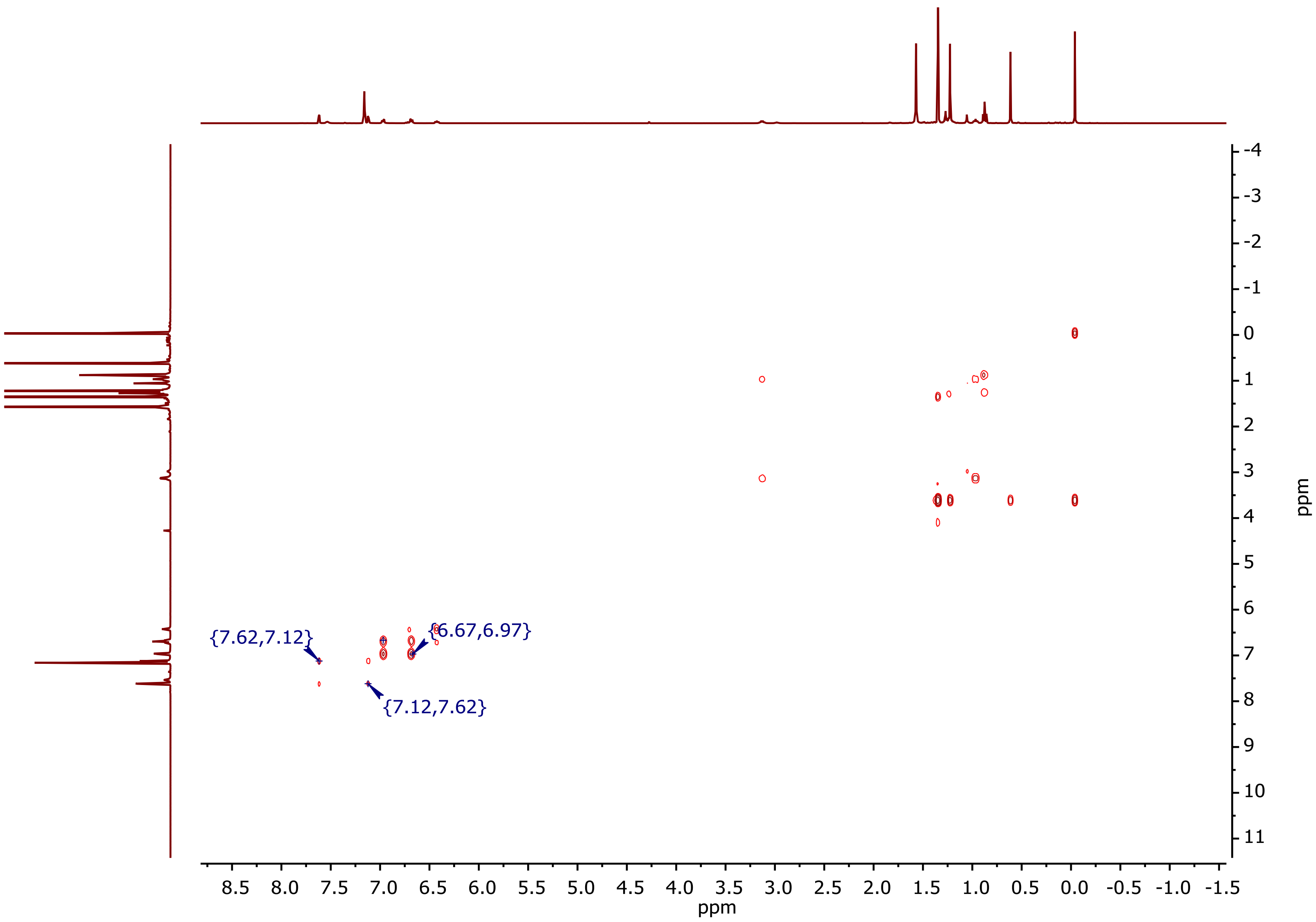


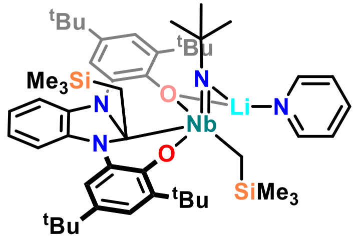


Figure S 51: $^{1}H$-$^{1}H$ COSY of **7** in $C_6D_6$ at 298 K.

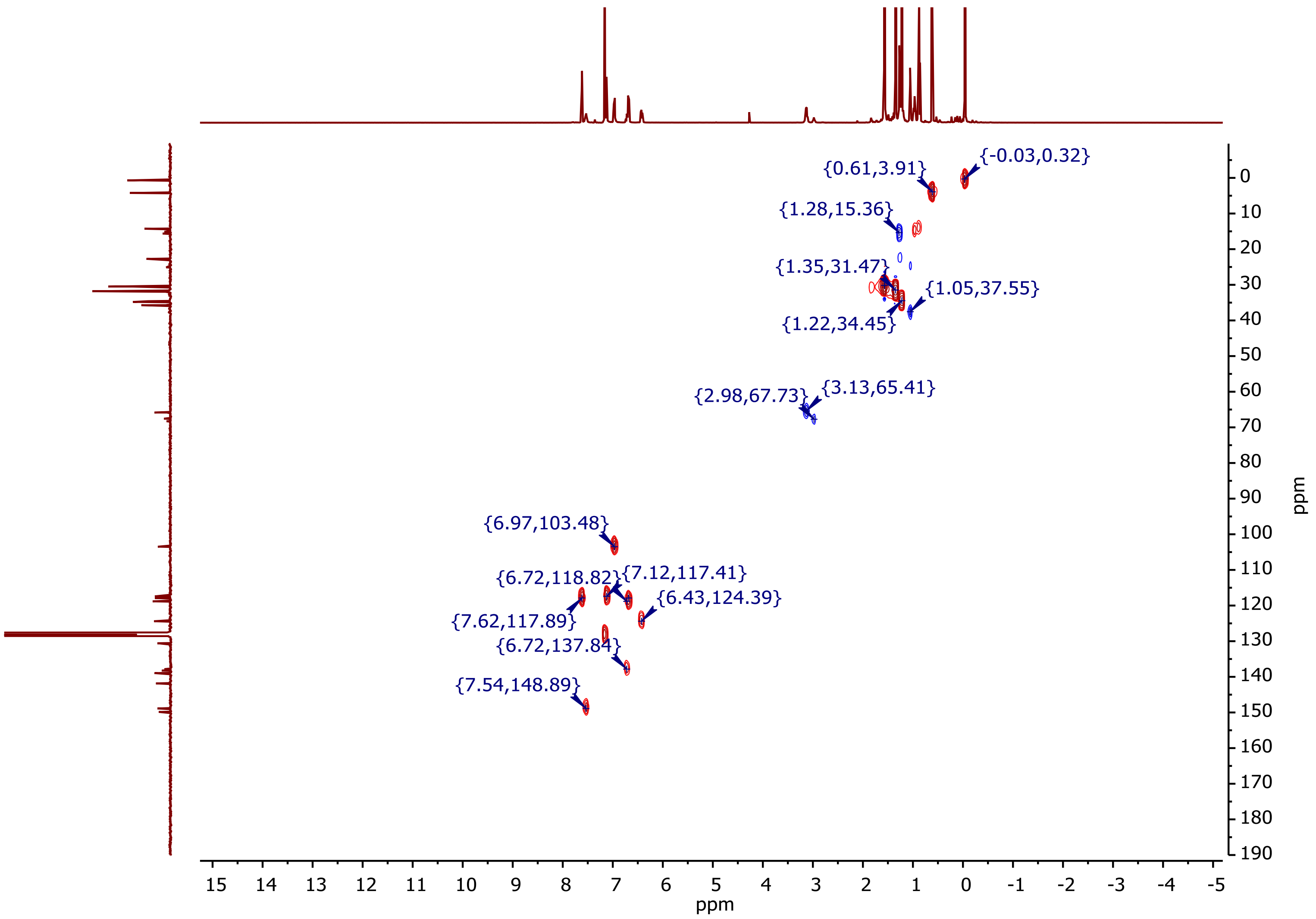


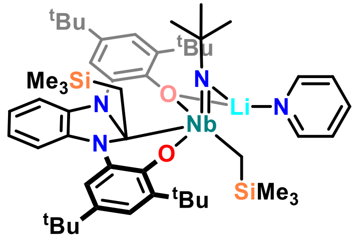


Figure S 52: $^{1}H$-$^{13}C\{^{1}H\}$ HSQC of **7** in $C_6D_6$ at 298 K.

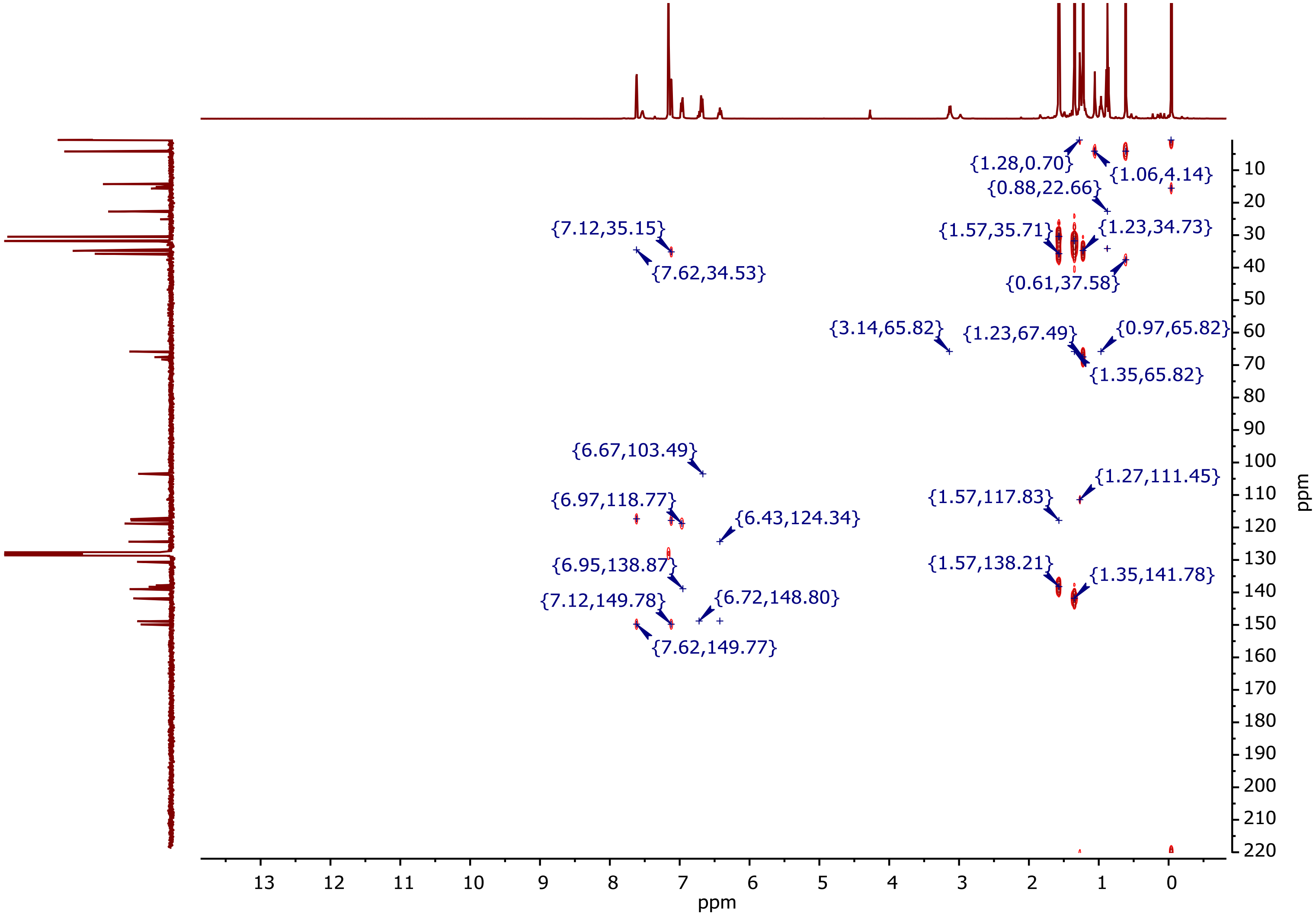


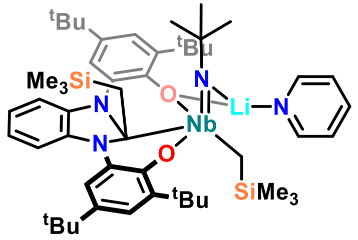


Figure S 53: $^{1}$H-$^{13}$C{$^{1}$H} HMBC of **7** in $C_6D_6$ at 298 K.

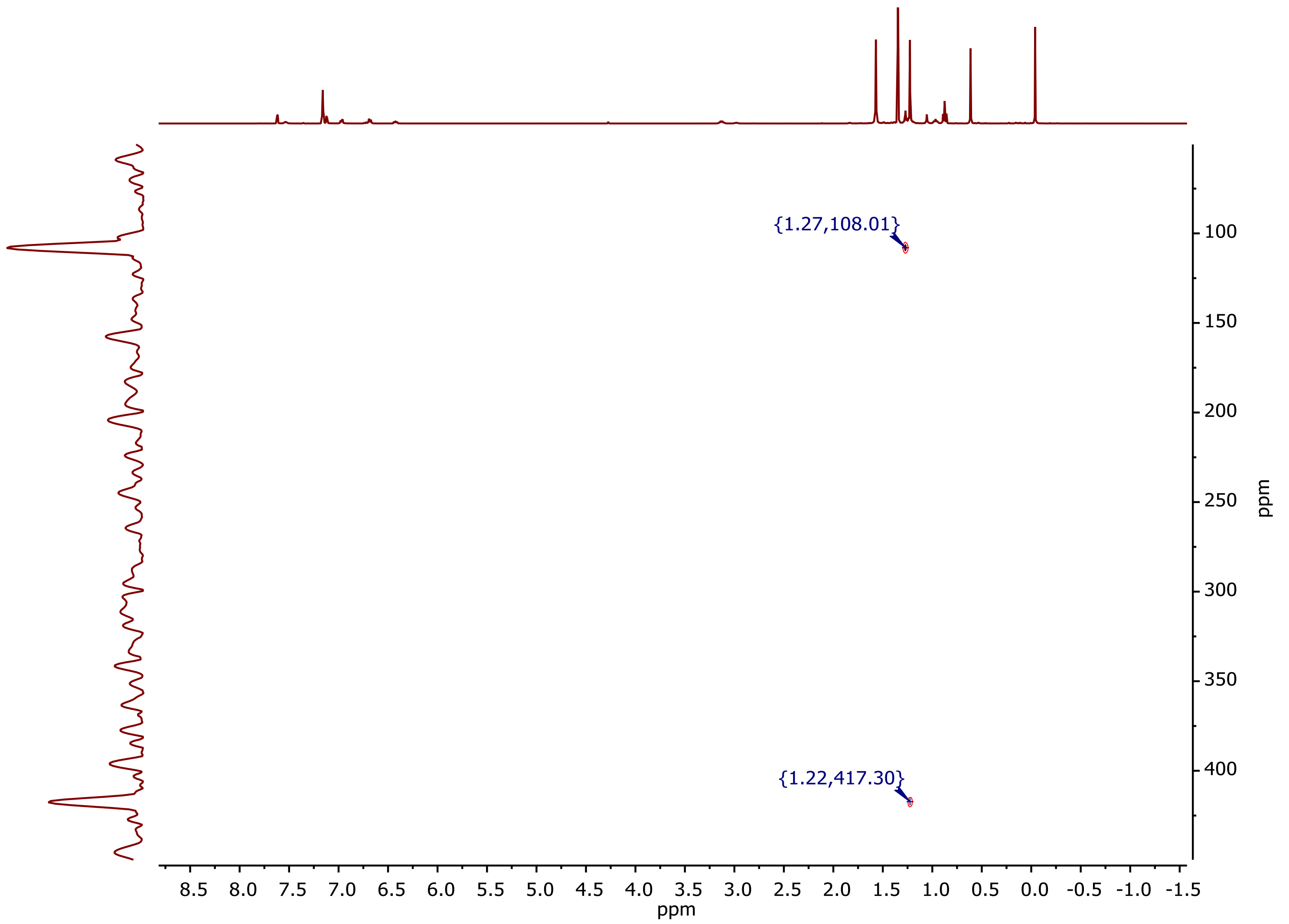


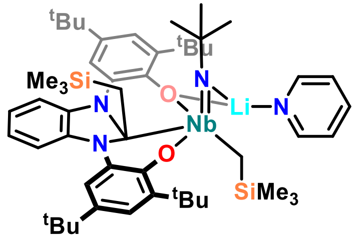

Figure S 54: $^{1}H$-$^{15}N$ HMBC of **7** in $C_6D_6$ at 298 K. (41 MHz)

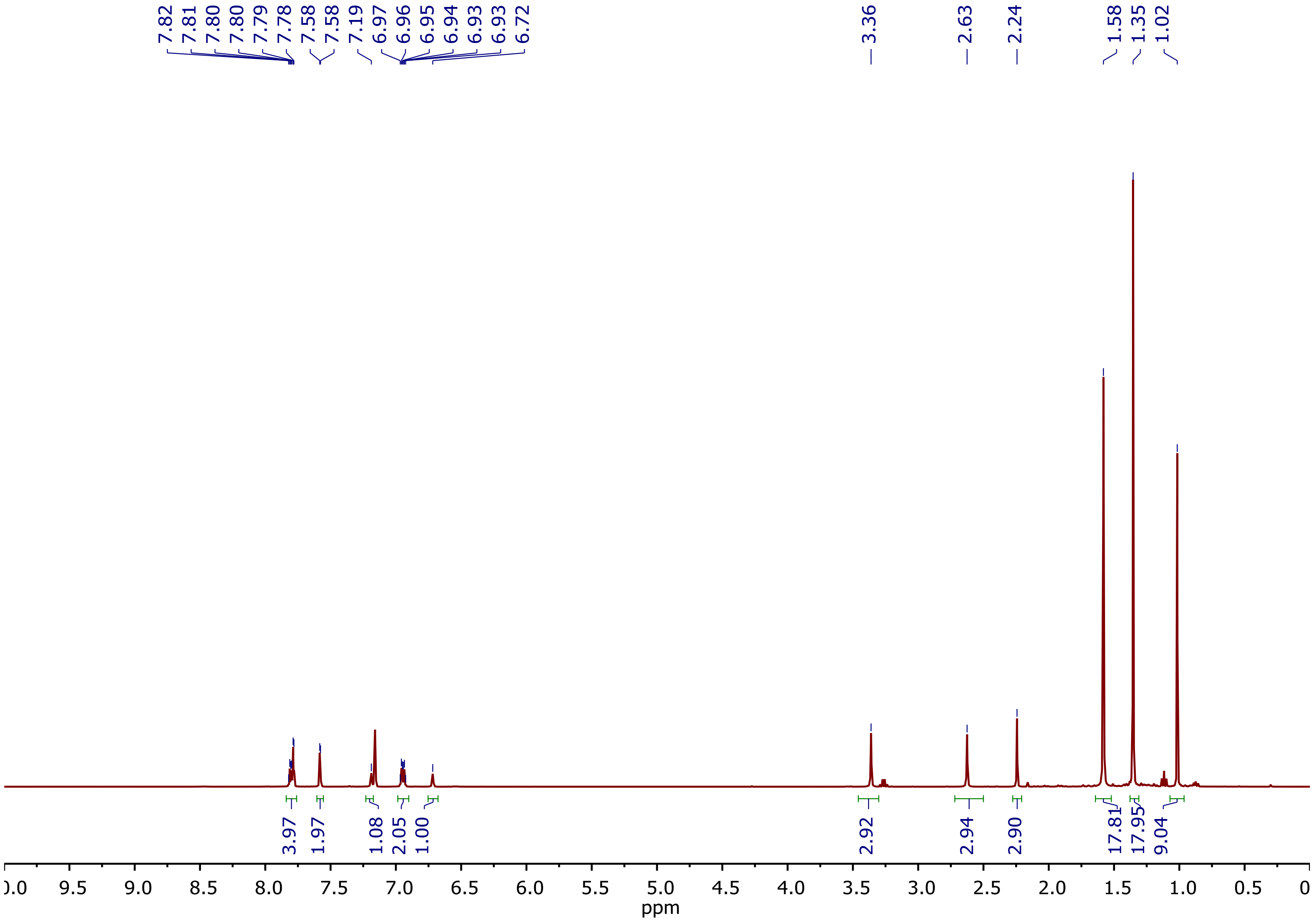

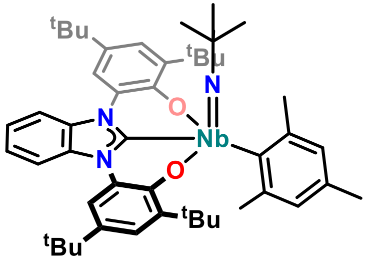


Figure S 55: $^{1}$H NMR of **8** in $C_6D_6$ at 298 K. (400 MHz)

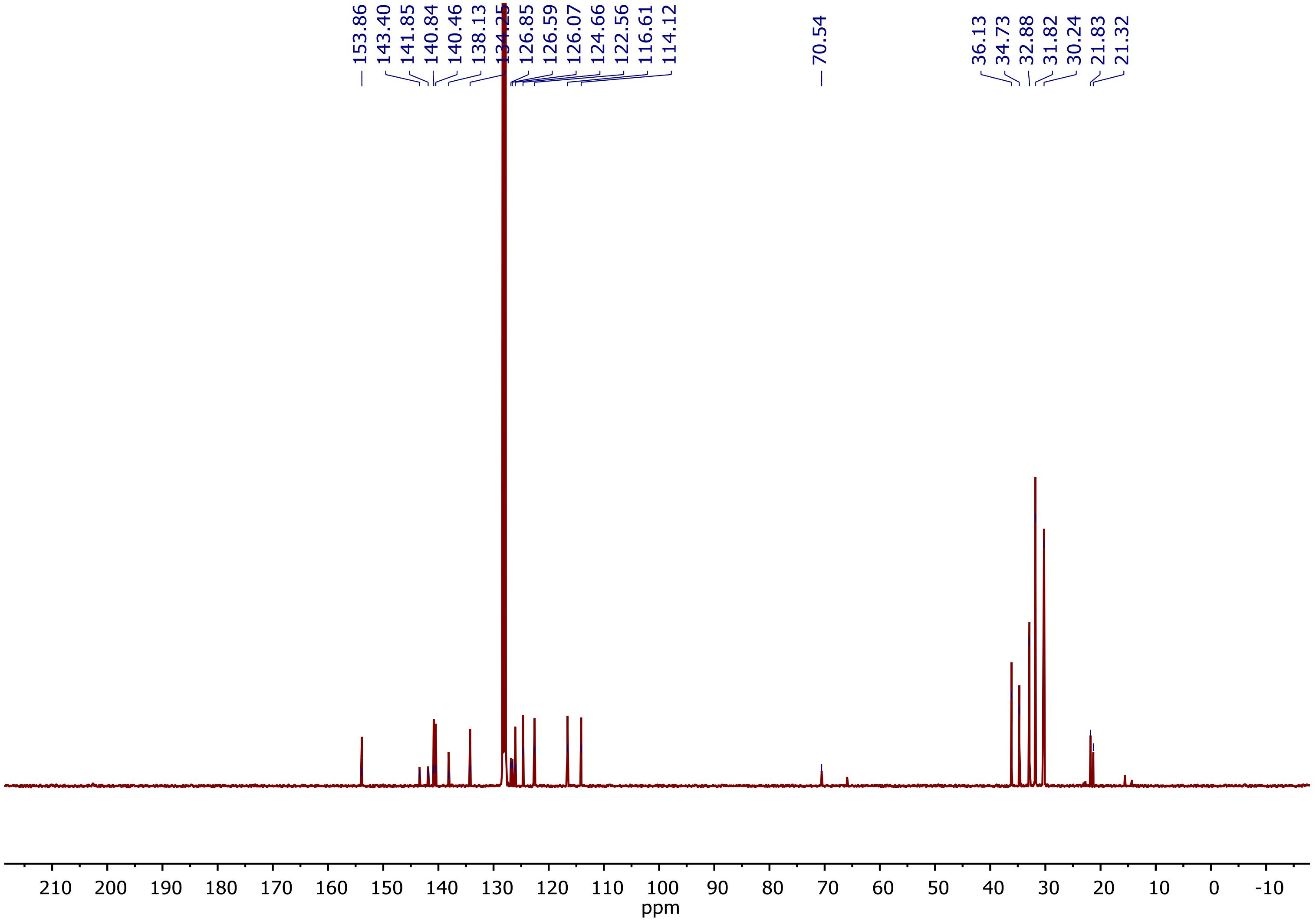


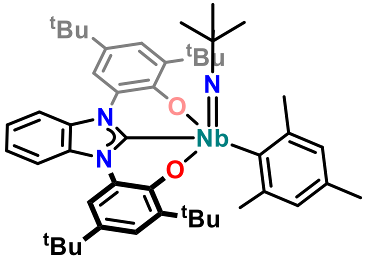


Figure S 56: $^{13}C\{^{1}H\}$ NMR of **8** in $C_6D_6$ at 298 K. (101 MHz)

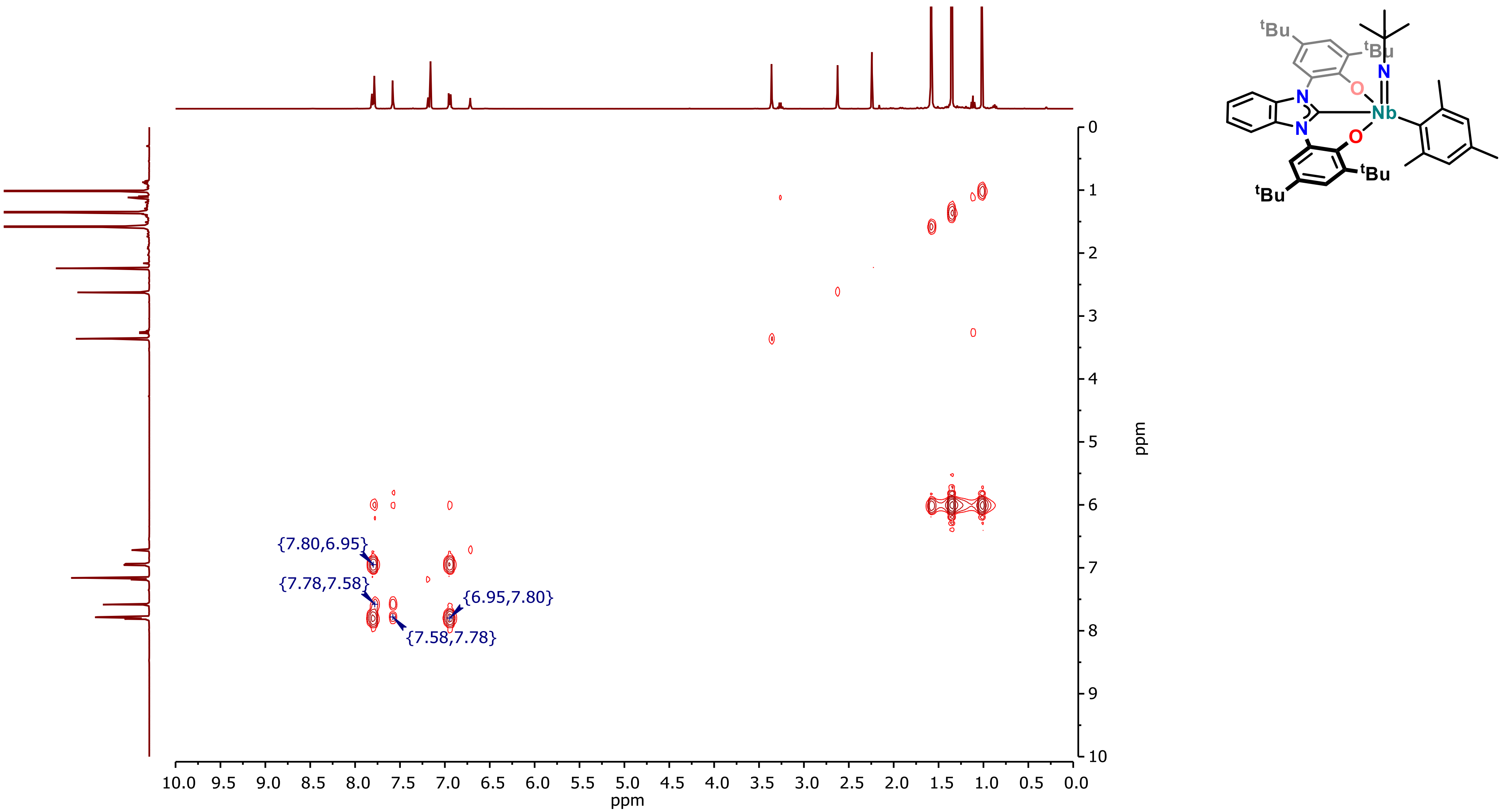


Figure S 57: $^{1}H$-$^{1}H$ COSY of **8** in $C_6D_6$ at 298 K.

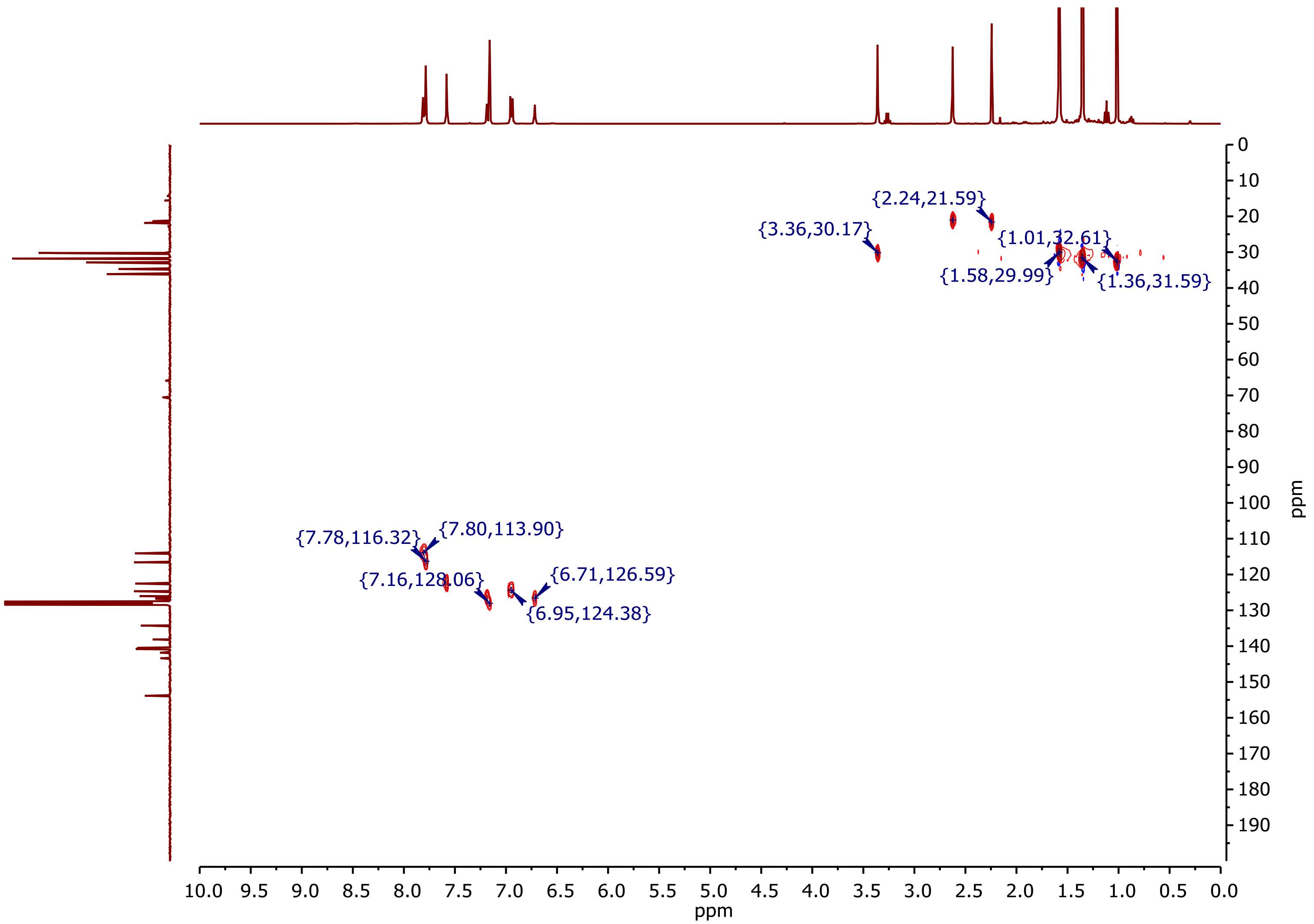


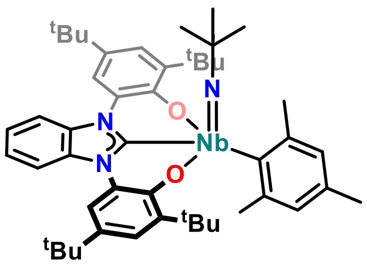


Figure S 58: $^{1}H$-$^{13}C\{^{1}H\}$ HSQC of **8** in $C_6D_6$ at 298 K.

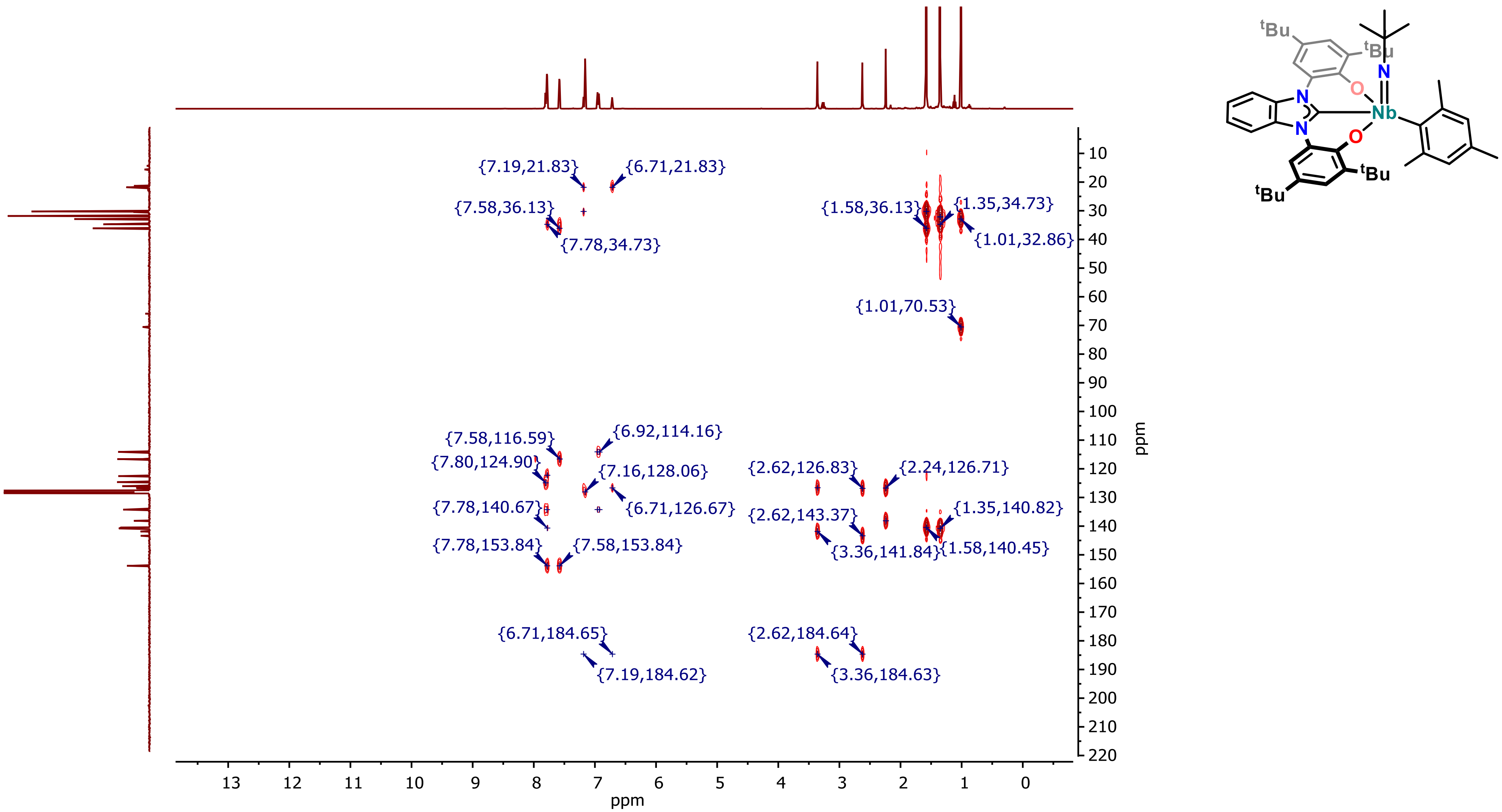


Figure S 59: $^{1}H$-$^{13}C\{^{1}H\}$ HMBC of **8** in $C_6D_6$ at 298K.

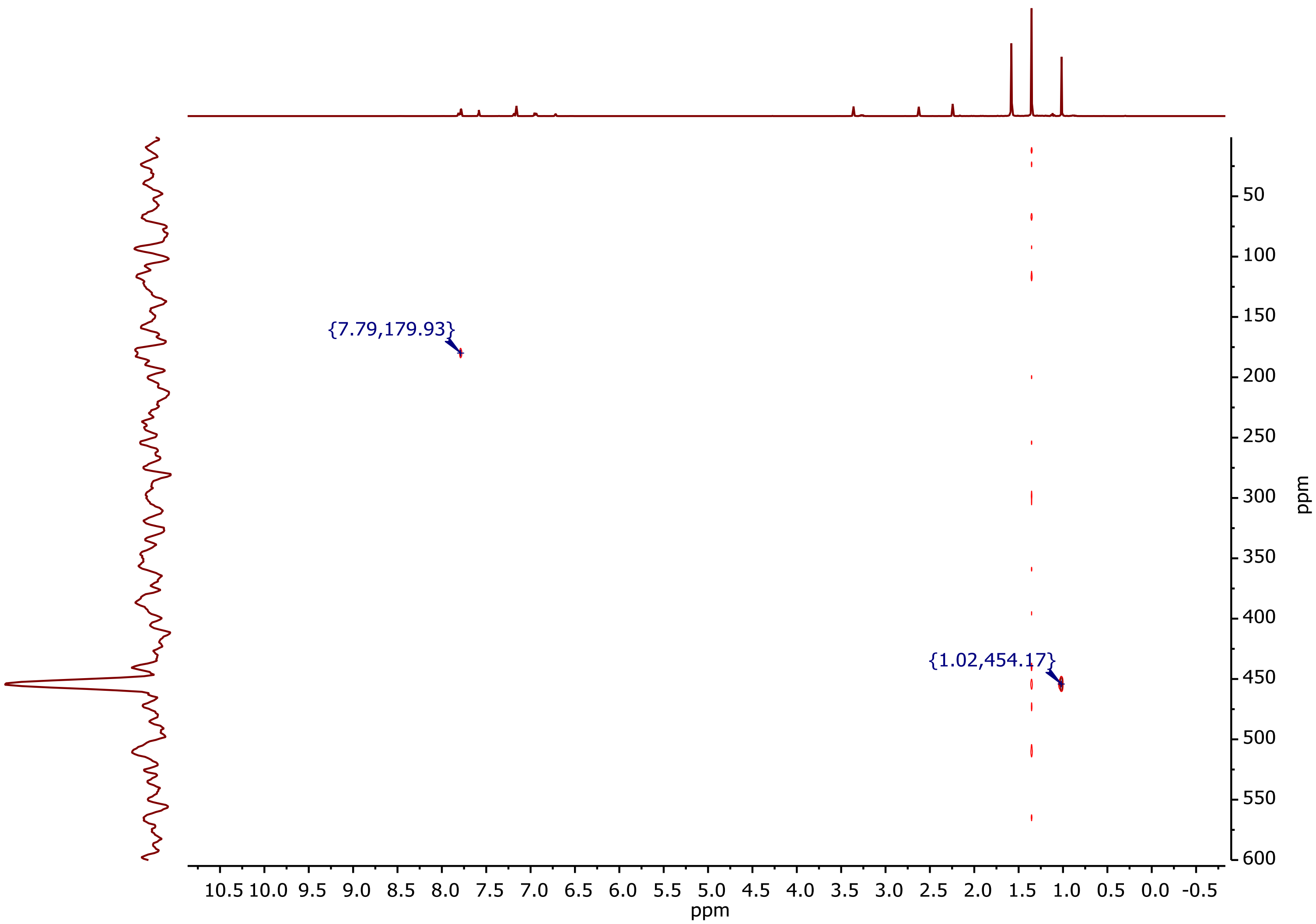


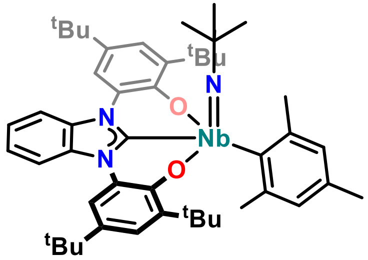


Figure S 60: $^{1}H$-$^{15}N$ HMBC of **8** in $C_6D_6$ at 298K. (41 MHz)

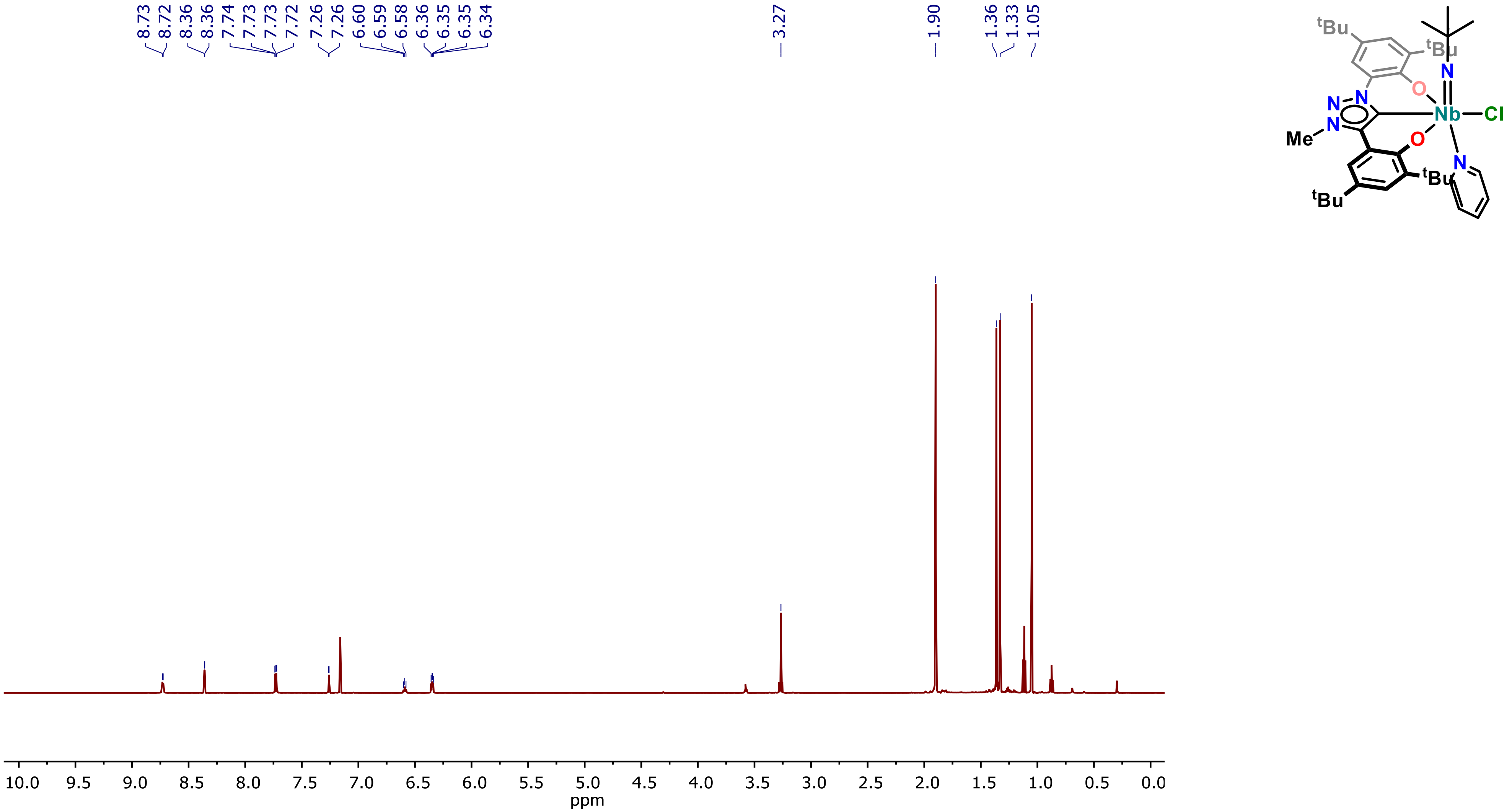


Figure S 61: $^{1}$H NMR of **I** (Figure 1, main manuscript) in $C_6D_6$ at 298 K (700 MHz).

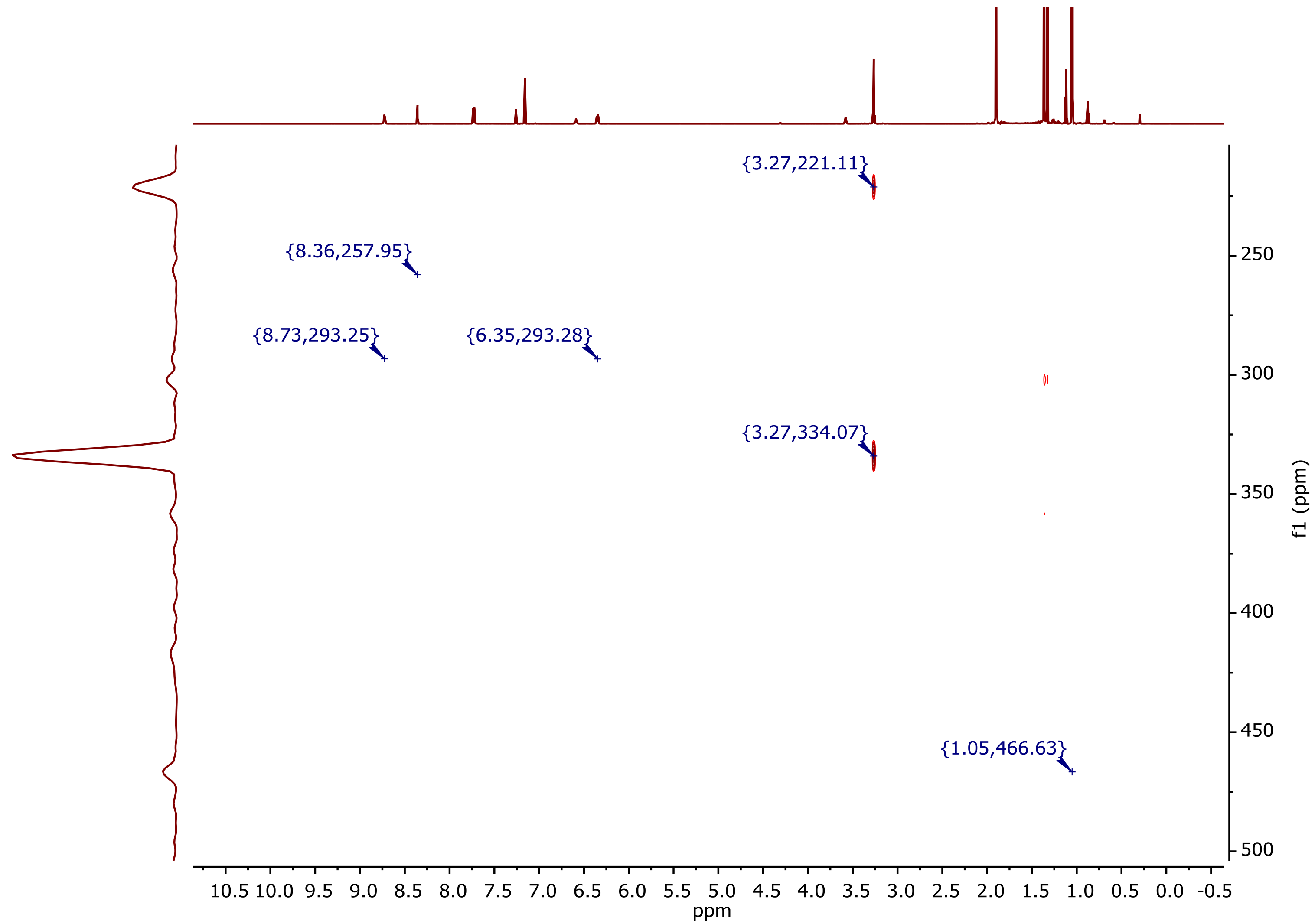


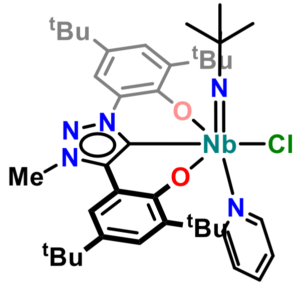


Figure S 62: $^{1}H$-$^{15}N$ HMBC of **I** (Figure 1, main manuscript) in $C_6D_6$ at 298 K (41 MHz).

## 2. IR spectroscopy

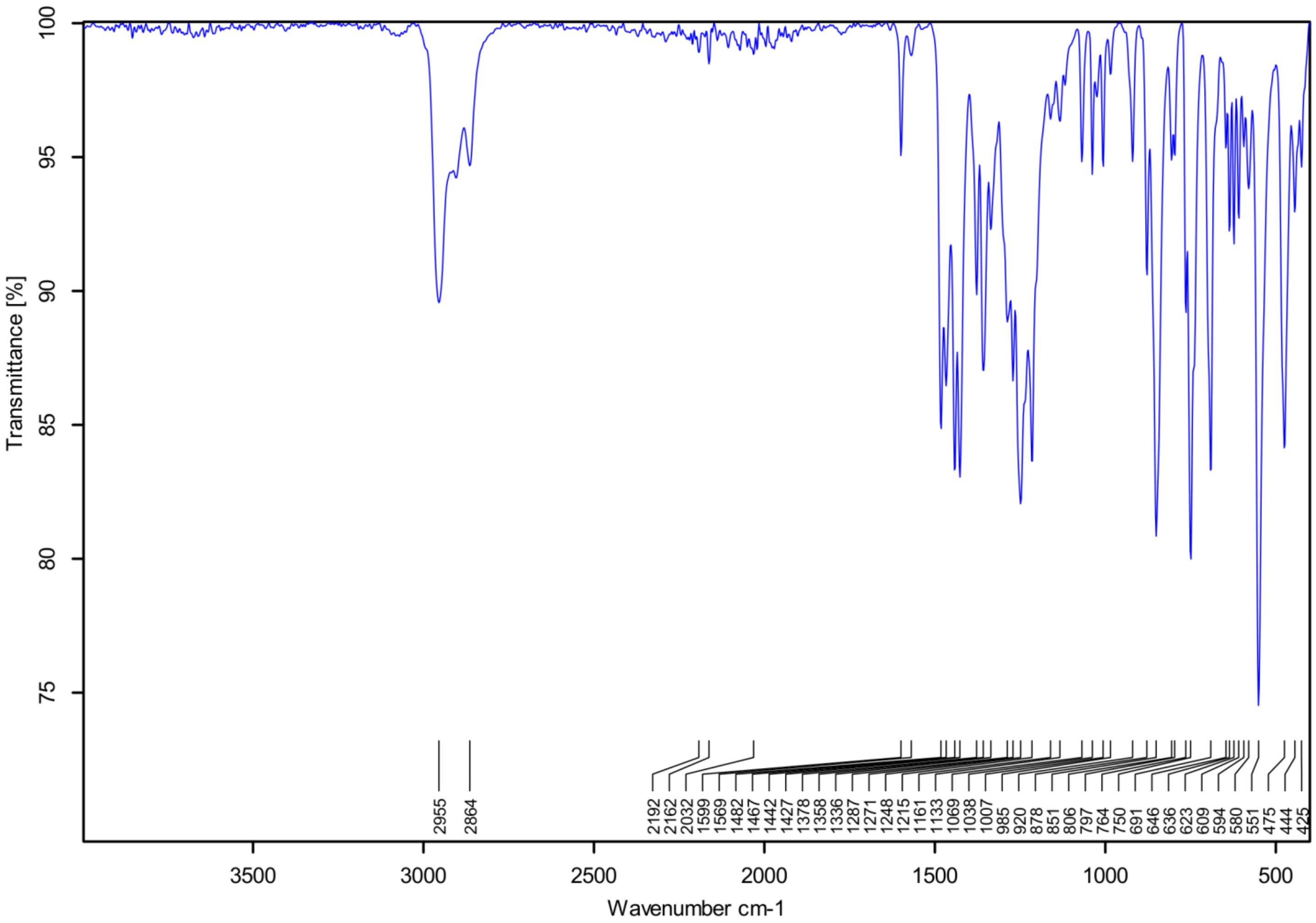


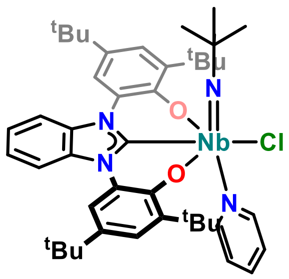


Figure S 63: ATR-IR spectrum of **1-Py** at 298 K.

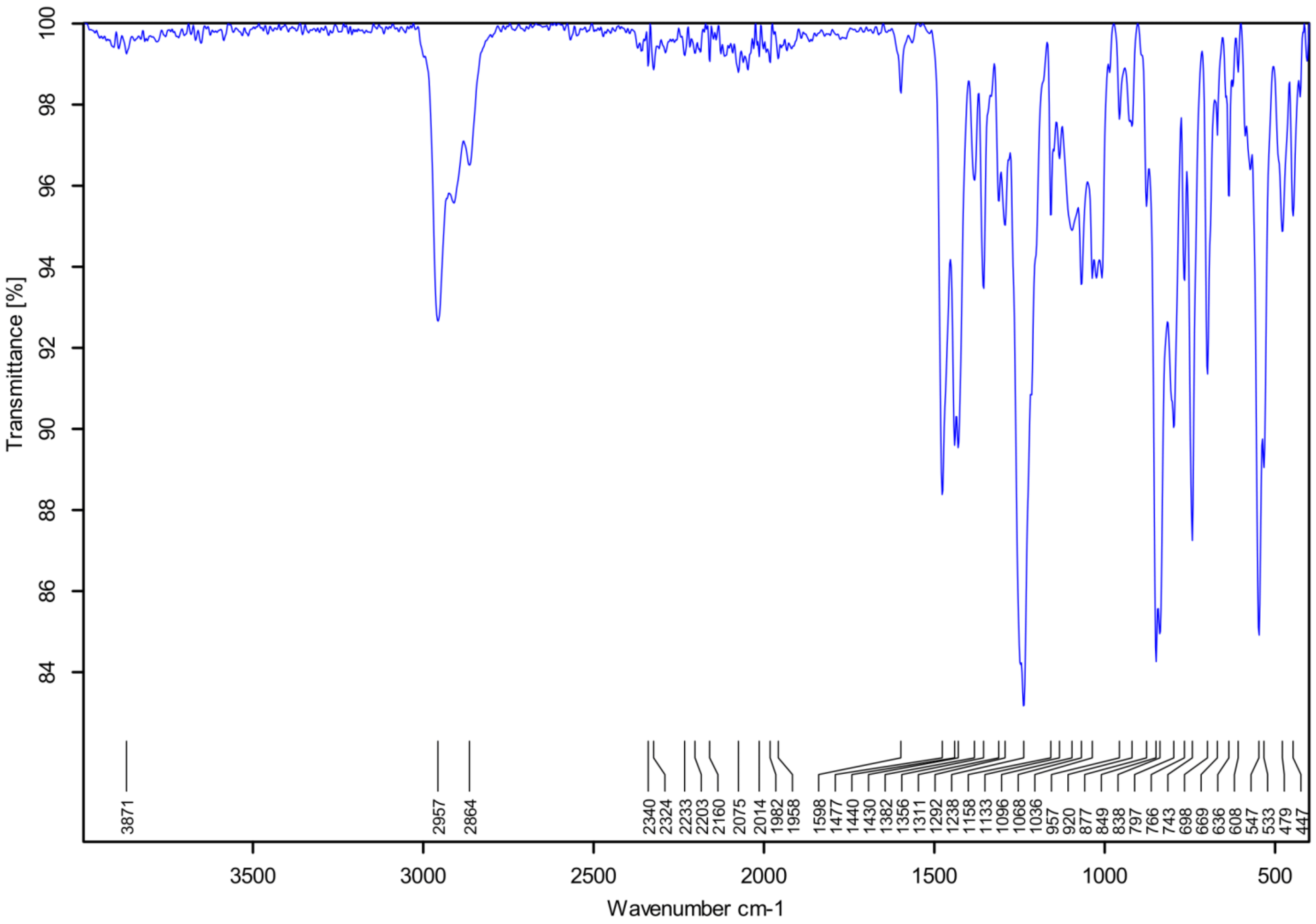


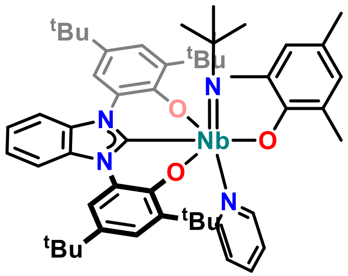


Figure S 64: ATR-IR spectrum of **2** at 298 K.

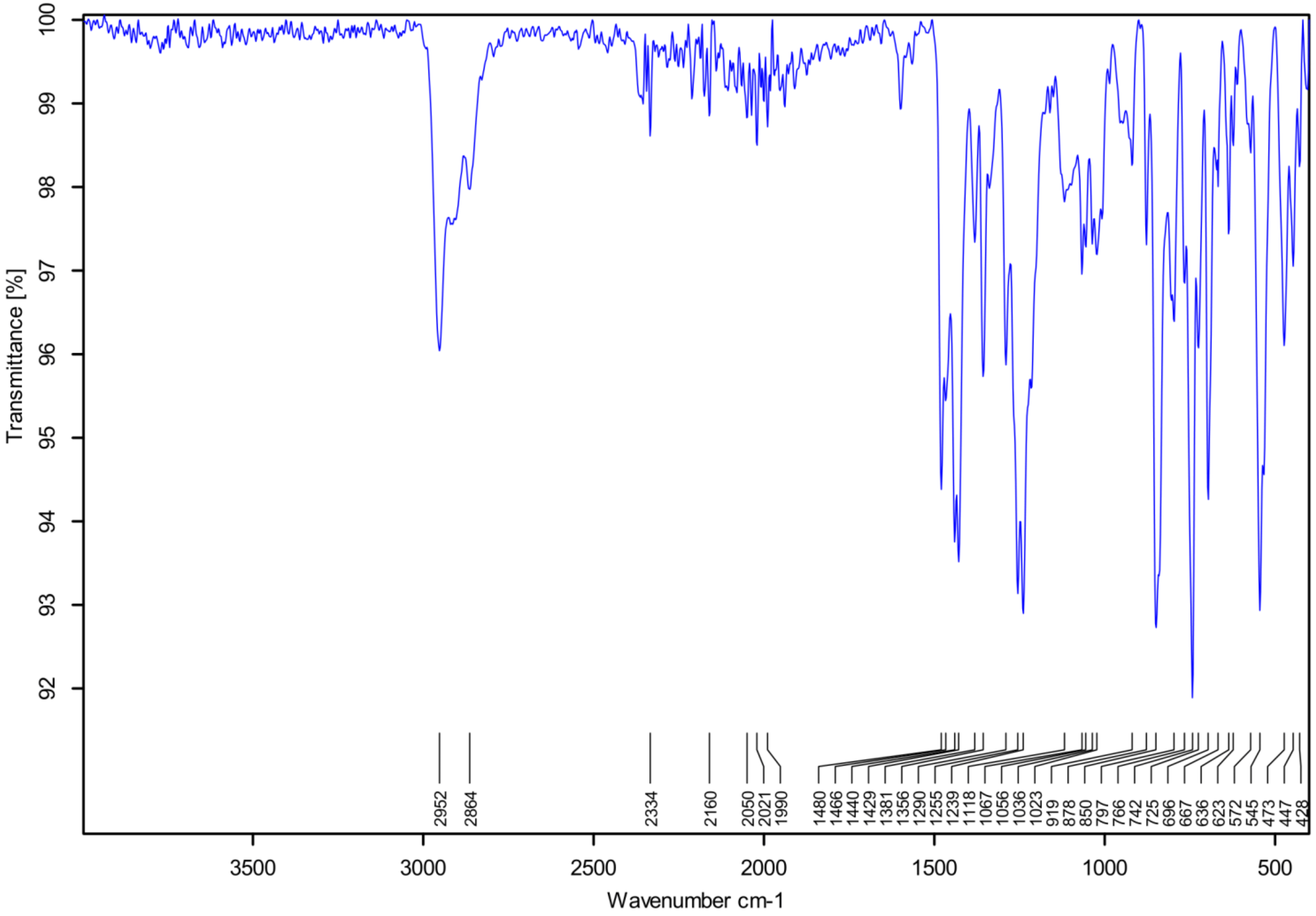


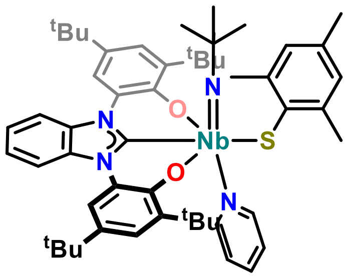


Figure S 65: ATR-IR spectrum of **3** at 298 K.

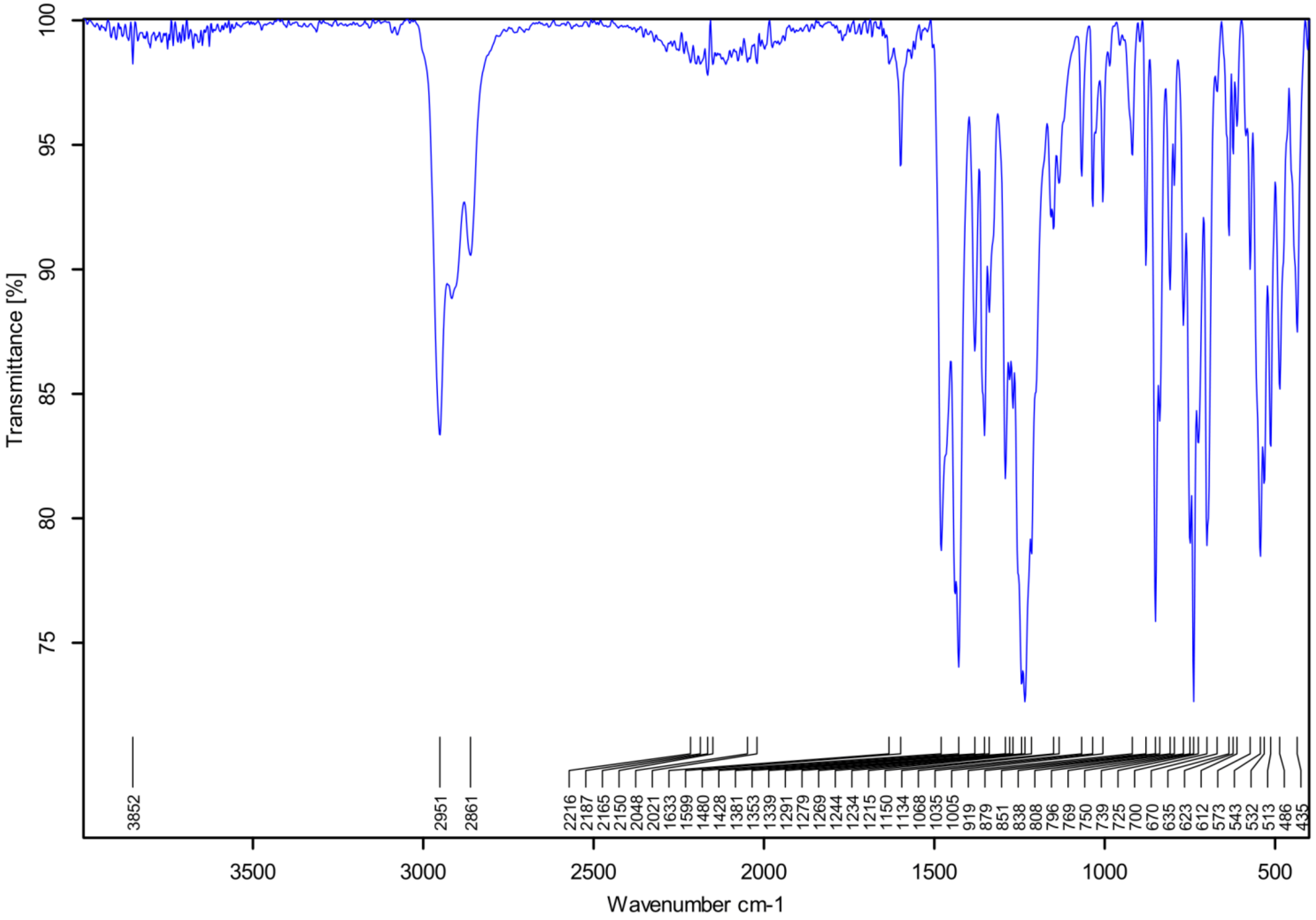


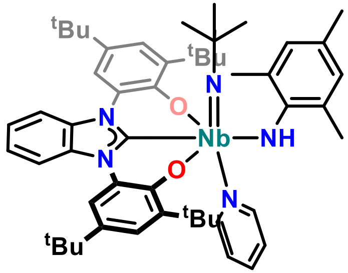


Figure S 66: ATR-IR spectrum of **4** at 298 K.

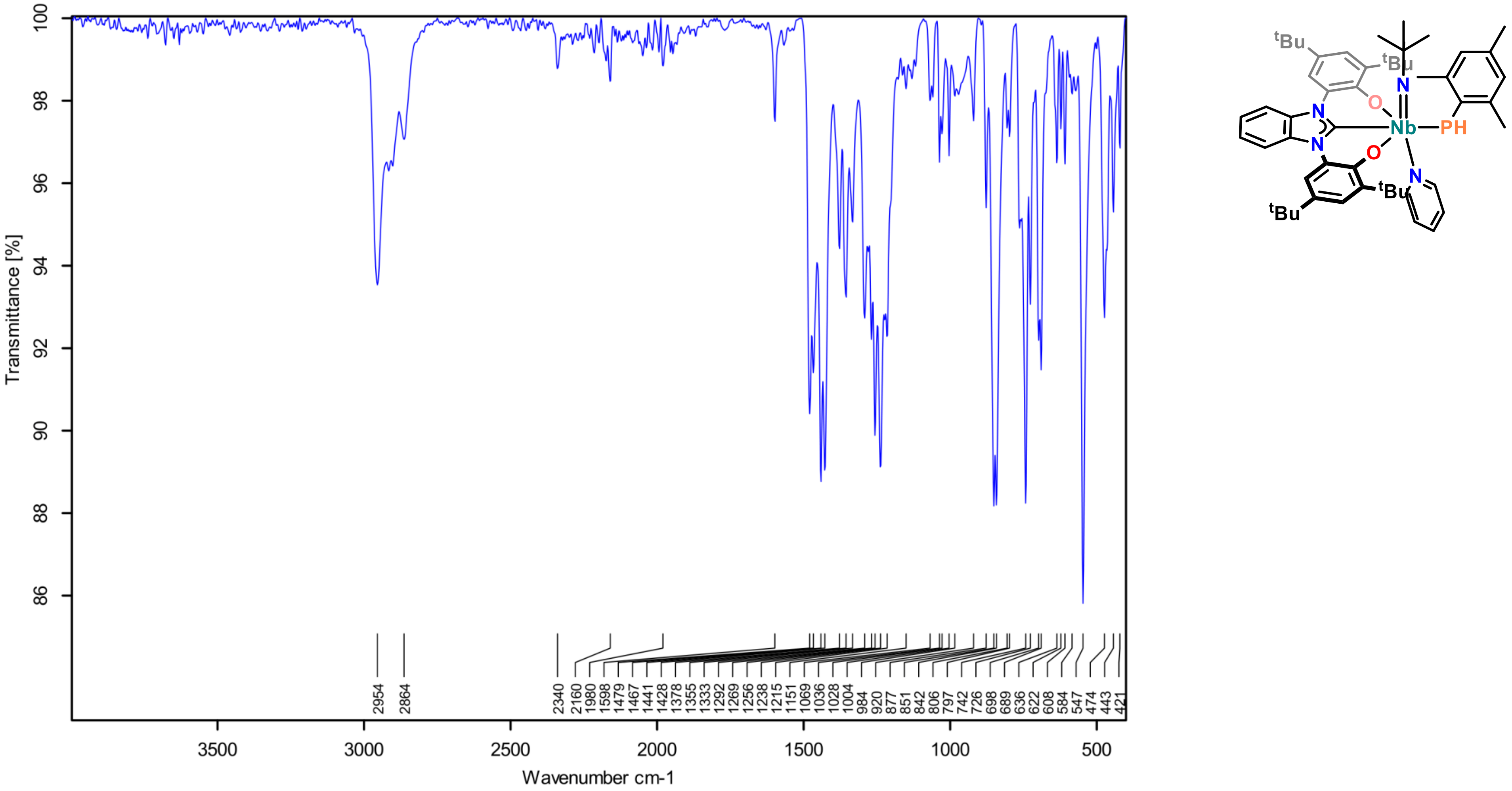


Figure S 67: ATR-IR spectrum of **5** at 298 K.

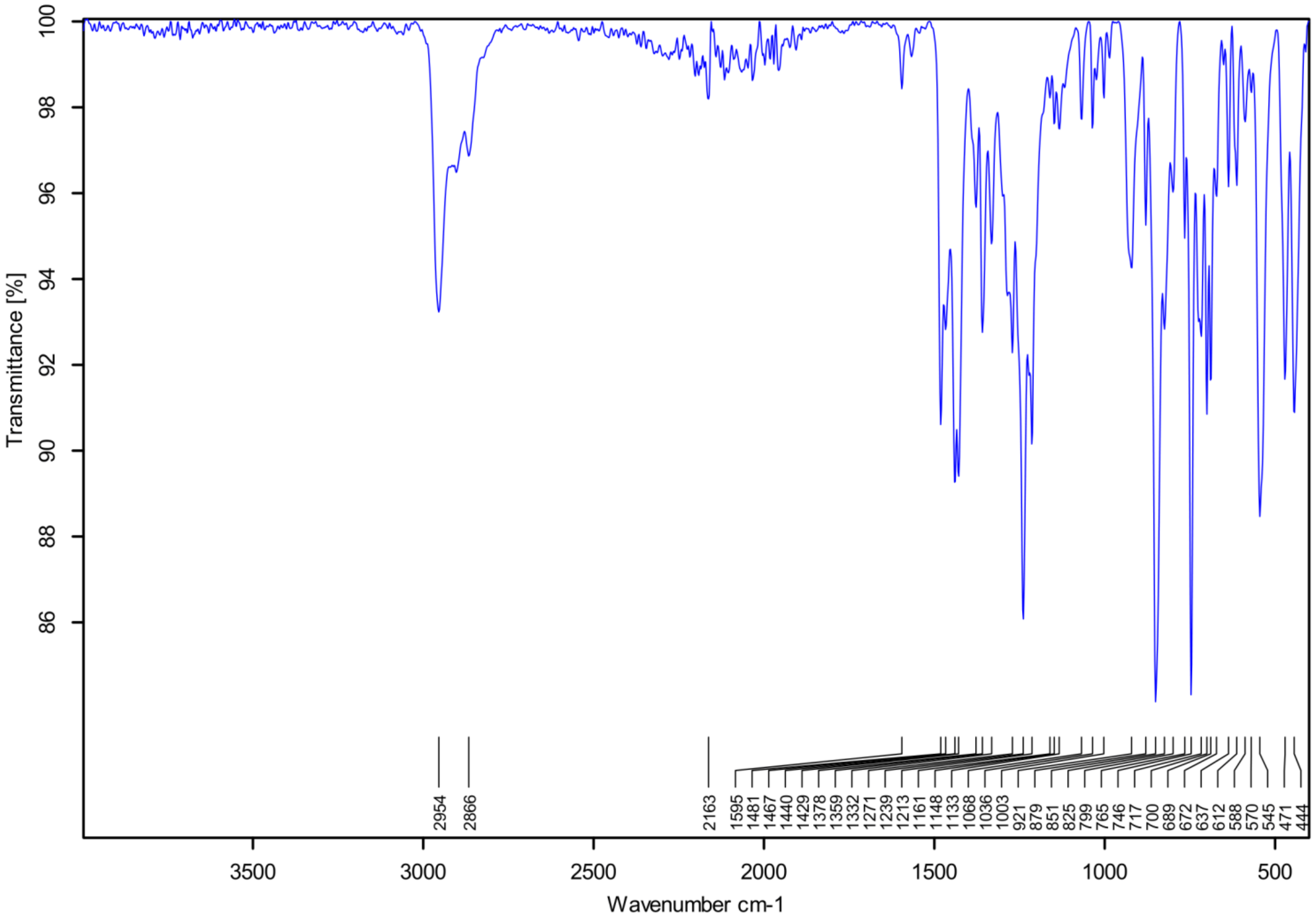


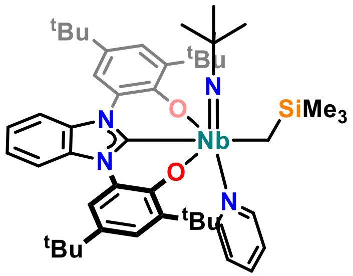


Figure S 68: ATR-IR spectrum of **6** at 298 K.

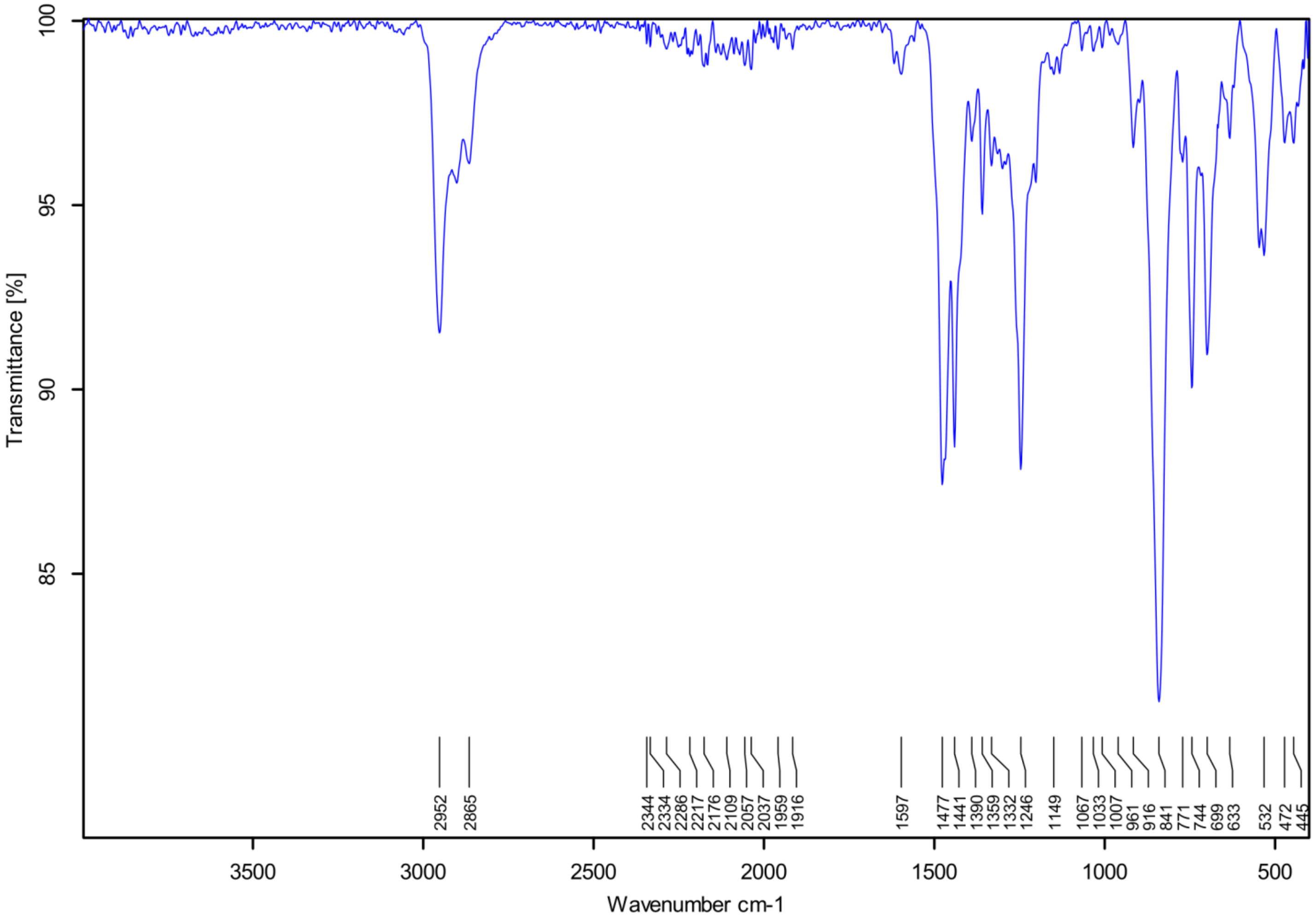


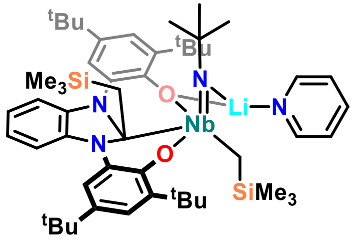

Figure S 69: ATR-IR spectrum of **7** at 298 K.

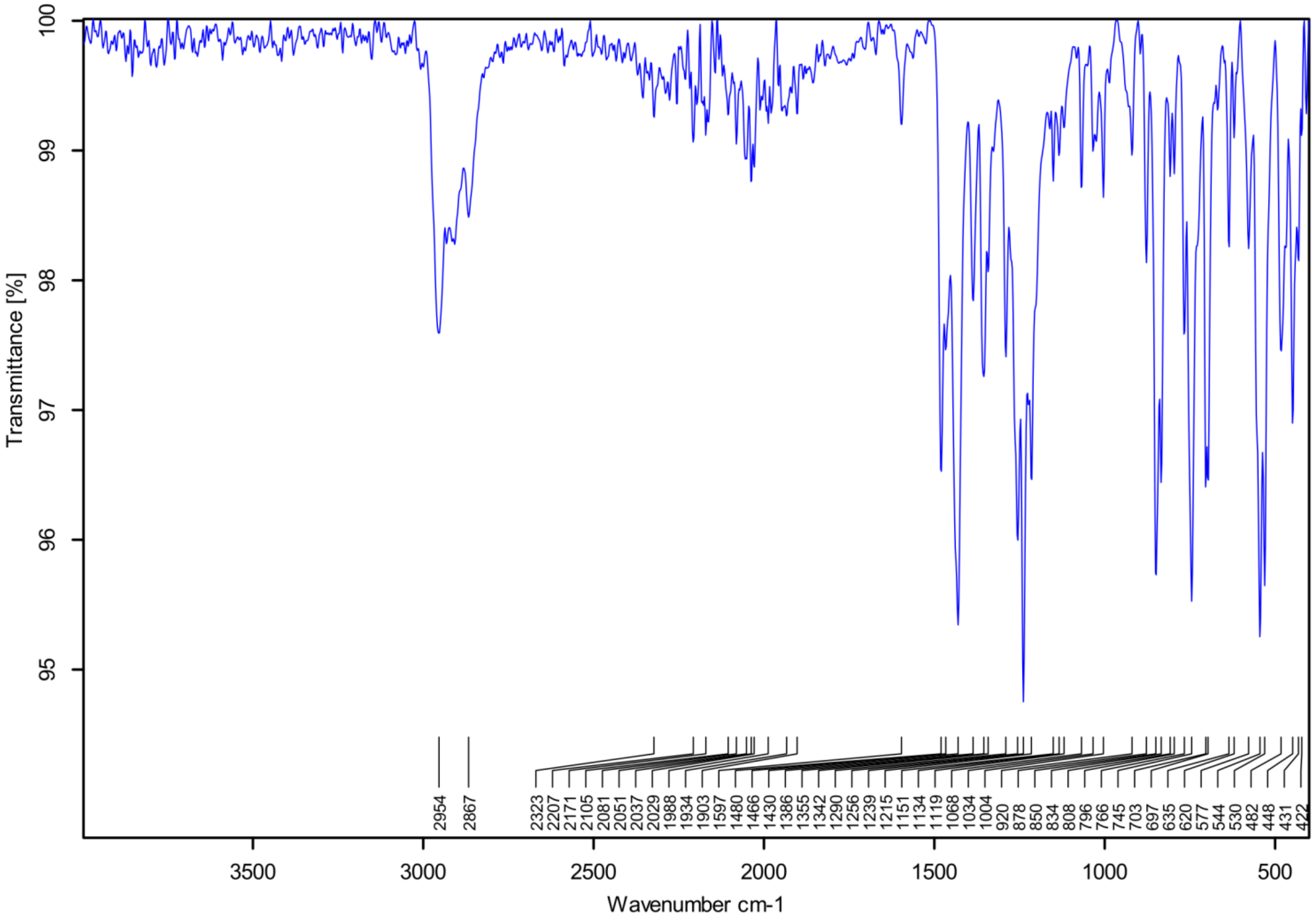


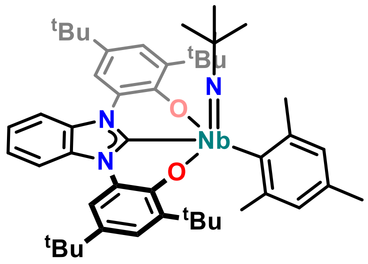


Figure S 70: ATR-IR spectrum of **8** at 298 K.

## 3. Crystallographic details

Table S 1: Crystallographic details

| | **1-Py*** | **1** | **3** | **4** | **5** | **6** | **7*** | **8** | **9** |
|---|---|---|---|---|---|---|---|---|---|
| Chemical formula | $2(C_{44}H_{58}N_4O_2Cl_1Nb_1)$ $0.3\ C_5H_{12}$ | $C_{39}H_{53}N_3O_2Cl_1Nb_1$ $0.5\ C_5H_{12}$ | $C_{53}H_{69}N_4O_2S_1Nb_1$ $0.5\ C_5H_{12}$ | $C_{53}H_{70}N_5O_2Nb_1$ $0.5\ C_5H_{12}$ | $C_{53}H_{70}N_4O_2P_1Nb_1$ $C_5H_{12}$ | $C_{48}H_{69}N_4O_2Si_1Nb_1$ $0.75\ C_5H_{12}$ | $C_{52}H_{80}N_4O_2Si_2Nb_1Li_1$ $0.5\ C_5H_{12}$ | $C_{48}H_{64}N_3O_2Nb_1$ | $C_{78}H_{106}N_6O_5Nb_1$ $1.5\ C_5H_{12}$ |
| $M_r$ | 1628.24 | 760.27 | 955.16 | 938.12 | 991.15 | 909.18 | 949.23 | 807.93 | 1501.72 |
| Crystal system | Monoclinic | Monoclinic | Triclinic | Triclinic | Monoclinic | Monoclinic | Monoclinic | Orthorhombic | Monoclinic |
| Space group | *P*2$_1$/*n* | *P*2$_1$/*c* | *P*-1 | *P*-1 | *P*2$_1$/*n* | *P*2$_1$/*c* | *P*2$_1$/*n* | *Pnma* | *C*2/*c* |
| a (Å) | 25.801(4) | 13.0070(4) | 11.5800(7) | 11.3250(8) | 12.0996(7) | 16.227(3) | 12.4099(6) | 23.8126(19) | 33.6152(14) |
| b (Å) | 12.0620(17) | 16.3443(5) | 16.2127(10) | 16.3810(12) | 16.6804(10) | 16.516(3) | 31.1910(13) | 19.4876(18) | 25.3256(8) |
| c (Å) | 32.671(5) | 22.1143(6) | 16.4471(9) | 16.6130(13) | 27.5729(13) | 39.203(6) | 15.9754(8) | 9.7872(7) | 21.2301(9) |
| α (°) | 90 | 90 | 113.960(2) | 112.757(3) | 90 | 90 | 90 | 90 | 90 |
| β (°) | 97.616(5) | 105.2260(10) | 97.789(2) | 93.198(3) | 93.995(2) | 95.282(3) | 93.991(2) | 90 | 98.342(2) |
| γ (°) | 90 | 90 | 92.358(2) | 99.987(3) | 90 | 90 | 90 | 90 | 90 |
| V (Å$^3$) | 10078(3) | 4536.3(2) | 2779.8(3) | 2773.2(4) | 5551.4(5) | 10462(3) | 6168.7(5) | 4541.8(6) | 17882.5(12) |
| Z | 4 | 4 | 2 | 2 | 4 | 8 | 4 | 4 | 8 |
| Densitiy (g cm$^{-3}$) | 1.073 | 1.113 | 1.141 | 1.123 | 1.186 | 1.154 | 1.022 | 1.182 | 1.116 |
| F(000) | 3442 | 1612 | 1018 | 1002 | 2120 | 3900 | 2032 | 1720 | 6408 |
| Radiation Type | MoKα | MoKα | MoKα | MoKα | MoKα | MoKα | MoKα | MoKα | MoKα |
| μ (mm$^{-1}$) | 0.326 | 0.357 | 0.294 | 0.258 | 0.288 | 0.293 | 0.269 | 0.303 | 0.304 |
| Crystal size | 0.49x0.45x0.42 | 0.18x0.16x0.15 | 0.15x0.12x0.08 | 0.10x0.08x0.07 | 0.10x0.05x0.04 | 0.21x0.18x0.15 | 0.20x0.10x0.05 | 0.25x0.24x0.22 | 0.20x0.15x0.12 |
| Meas. Refl. | 221056 | 147181 | 71590 | 69222 | 144156 | 115352 | 155507 | 21161 | 242541 |
| Indep. Refl. | 23257 | 10418 | 10615 | 9833 | 12759 | 19357 | 14211 | 4264 | 19631 |
| Obsvd. [$I > 2\sigma(I)$] | 17348 | 9289 | 8596 | 6756 | 10370 | 13080 | 11634 | 2797 | 14878 |
| $R_{int}$ | 0.05588 | 0.0503 | 0.0714 | 0.1442 | 0.0448 | 0.1099 | 0.0659 | 0.1330 | 0.0616 |
| R [$F^2 > 2\sigma(F^2)$] | 0.0495 | 0.0428 | 0.0472 | 0.0599 | 0.0686 | 0.0632 | 0.0588 | 0.0901 | 0.0386 |
| wR($F^2$) | 0.1240 | 0.1159 | 0.1433 | 0.1835 | 0.1414 | 0.1411 | 1571 | 0.2102 | 0.1120 |
| S | 1.038 | 1.067 | 1.059 | 1.040 | 1.183 | 1.031 | 1.081 | 1.092 | 1.040 |
| $\Delta\rho_{max}$ | 0.436 | 0.917 | 0.951 | 0.695 | 1.075 | 0.714 | 0.396 | 1.393 | 0.514 |
| $\Delta\rho_{min}$ | -0.565 | -0.427 | -0.830 | -0.744 | -0.880 | -1.016 | -0.541 | -1.666 | -0.464 |
| CCDC | 2171514 | 2207809 | 2171518 | 2171513 | 2171516 | 2171515 | 2172680 | 2171512 | 2171517 |

* Due to heavily disordered solvent molecules (pentane), the SQUEEZE algorithm was applied.

Table S 2: Selected Bond lengths and angles

| | 1-Py | 1 | 3 | 4 | 5 | 6 | 7 | 8 | 9 |
|---|---|---|---|---|---|---|---|---|---|
| Nb1 – C1 | 2.260(2) | 2.261(2) | 2.244(3) | 2.254(4) | 2.287(3) | 2.310(4) | 2.235(3) | 2.310(9) | 2.292(2) / 2.277(2) |
| Nb1 – O1 | 1.9824(16) | 1.9632(16) | 2.011(2) | 2.036(3) | 2.000(2) | 1.983(3) | 1.9223(19) | 1.994(5) | 1.9744(15) / 1.9906(15) |
| Nb1 – O2 | 1.9740(17) | 1.9631(16) | 2.016(2) | 2.030(3) | 1.988(2) | 1.994(3) | 2.0678(19) | - | 1.9940(15) / 1.9907(15) |
| Nb1 – N40 | 1.756(2) | 1.739(2) | 1.762(3) | 1.764(4) | 1.761(3) | 1.749(3) | 1.812(3) | 1.758(9) | 1.7518(18) / 1.736(2) |
| Nb1 – N50 | 2.509(2) | - | 2.458(3) | 2.480(4) | 2.498(3) | 2.550(3) | - | - | - |
| Nb1 – X* | 2.4276(7) | 2.4006(6) | 2.4880(8) | 2.065(4) | 2.5977(11) | 2.230(4) | 2.231(3) | 2.229(9) | 1.9198(15) / 1.9280(15) |
| | | | | | | | | | |
| N1 – C8 | 1.436(3) | 1.432(3) | 1.434(4) | 1.407(5) | 1.437(4) | 1.447(5) | 1.404(3) | 1.450(9) | 1.436(3) / 1.435(3) |
| C8 – C13 | 1.398(3) | 1.402(3) | 1.398(5) | 1.419(6) | 1.411(4) | 1.393(6) | 1.404(3) | 1.403(9) | 1.400(3) / 1.397(3) |
| C13 – O1 | 1.339(3) | 1.351(3) | 1.350(4) | 1.356(5) | 1.340(4) | 1.343(5) | 1.360(3) | 1.338(8) | 1.339(2) / 1.344(3) |
| | | | | | | | | | |
| N2 – C14 | 1.439(3) | 1.437(3) | 1.431(5) | 1.442(5) | 1.446(4) | 1.440(5) | 1.404(3) | - | 1.438(3) / 1.441(3) |
| C14 – C19 | 1.394(4) | 1.398(3) | 1.417(4) | 1.413(6) | 1.401(4) | 1.403(6) | 1.408(4) | - | 1.400(3) / 1.394(3) |
| C19 – O2 | 1.335(3) | 1.347(3) | 1.333(4) | 1.338(5) | 1.338(4) | 1.339(5) | 1.365(3) | - | 1.339(3) / 1.338(3) |
| | | | | | | | | | |
| C1-N1 | 1.363(3) | 1.360(3) | 1.358(4) | 1.359(5) | 1.361(4) | 1.363(5) | 1.482(3) | 1.348(8) | 1.368(3) / 1.360(3) |
| C1-N2 | 1.402(3) | 1.359(3) | 1.358(4) | 1.365(5) | 1.416(4) | 1.359(5) | 1.478(3) | - | 1.360(3) / 1.357(3) |
| | | | | | | | | | |
| O1 – Nb1 – O2 | 151.67(7) | 140.94(7) | 151.39(9) | 150.20(12) | 149.14(9) | 148.64(12) | 154.41(9) | 139.2(3) | 139.24(6) / 137.67(7) |
| N40 – Nb1 – N50 | 172.40(9) | - | 175.63(10) | 173.55(14) | 174.73(11) | 169.35(14) | - | - | - |
| N40 – Nb1 – X* | 104.75(7) | 107.33(3) | 108.28(9) | 105.86(16) | 105.02(9) | 107.47(16) | 115.89(13) | 113.5(4) | 110.83(7) / 111.46(8) |
| C1 – Nb1 – X* | 158.44(6) | 155.36(6) | 154.91(8) | 158.68(16) | 158.93(9) | 157.94(15) | 134.21(11) | 151.6(4) | 150.72(7) / 153.82(8) |
| | | | | | | | | | |
| $\tau_5$ | - | 0.24 | - | - | - | - | 0.37 | 0.21 | 0.19 / 0.27 |

*X = O10, S60, N60, P60, C60, Cl1

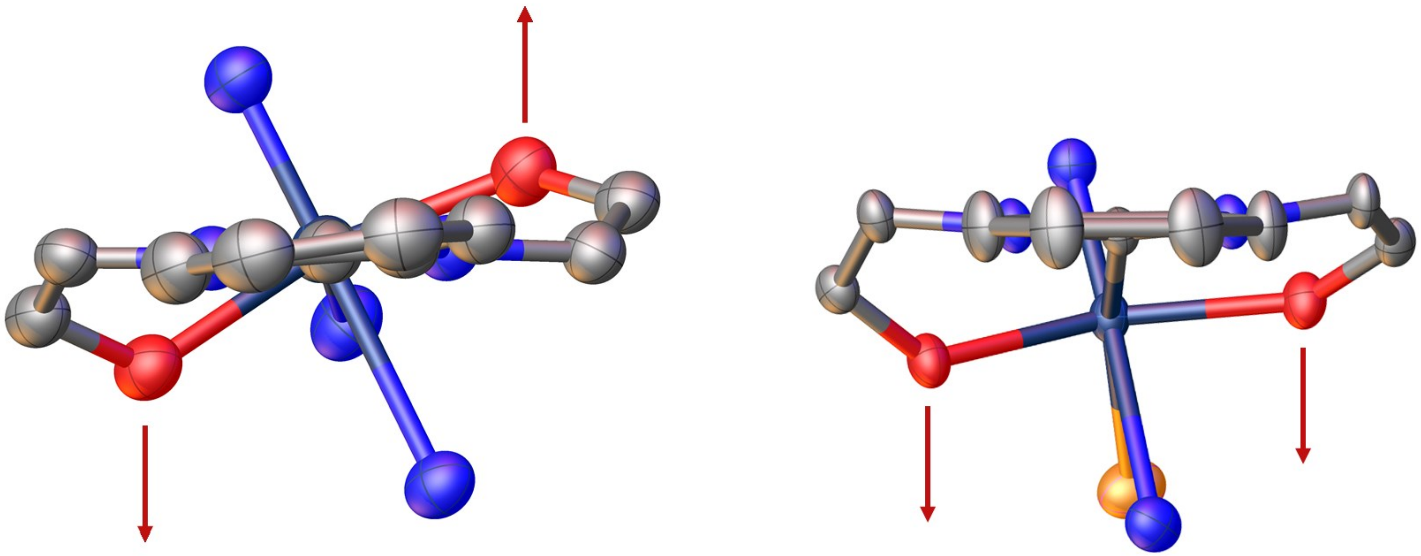

Figure S 71: View along the benzimidazole-2-ylidene plane in the truncated molecular structures of **4** (left) and **5** (right) to visualize the relative orientation of phenolate linkers causing the observation of a pitch angle or not.

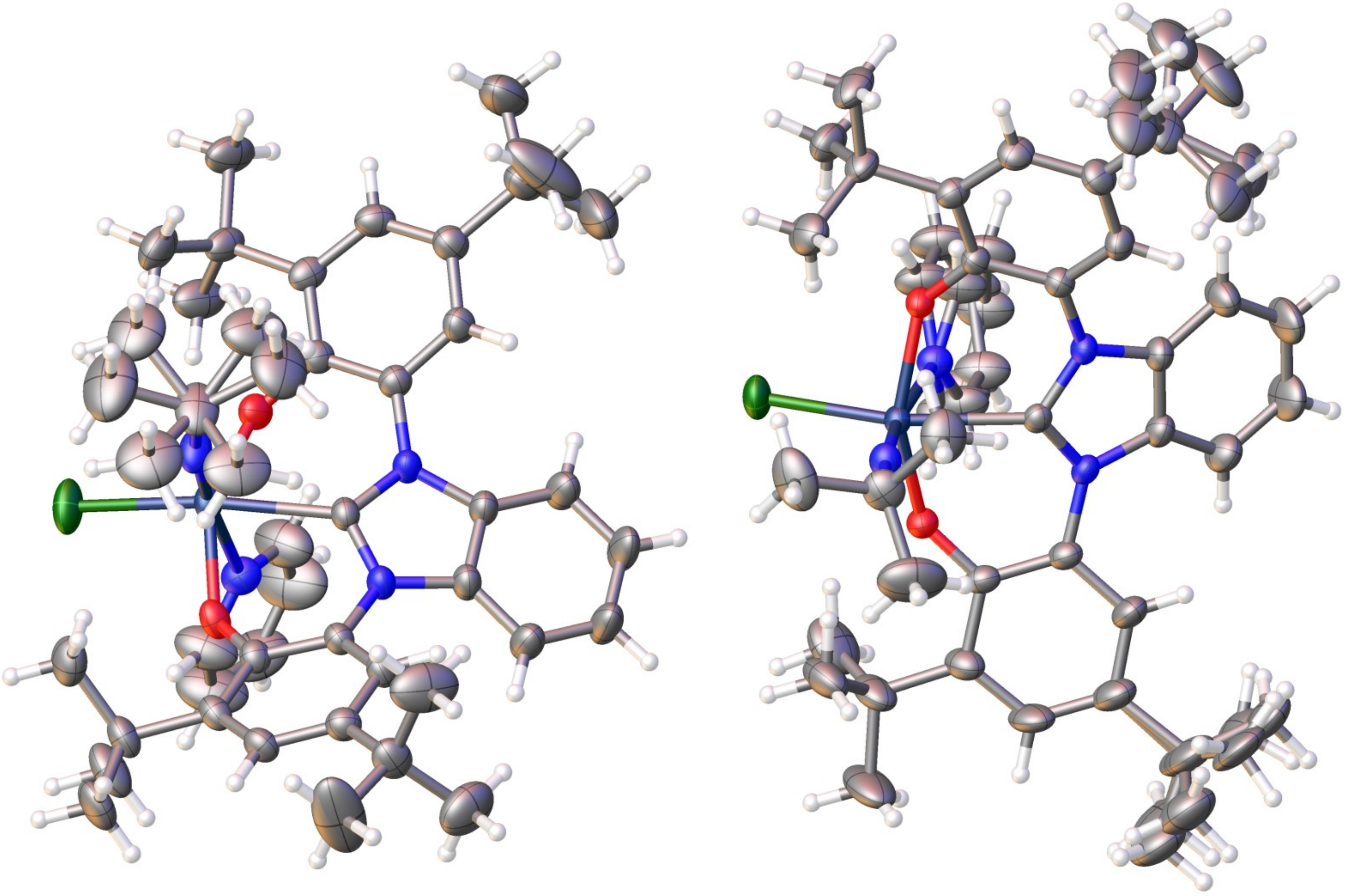

Figure S 72: Full Molecular structure of **1-Py**. Ellipsoids are shown at a probability level of 50%.

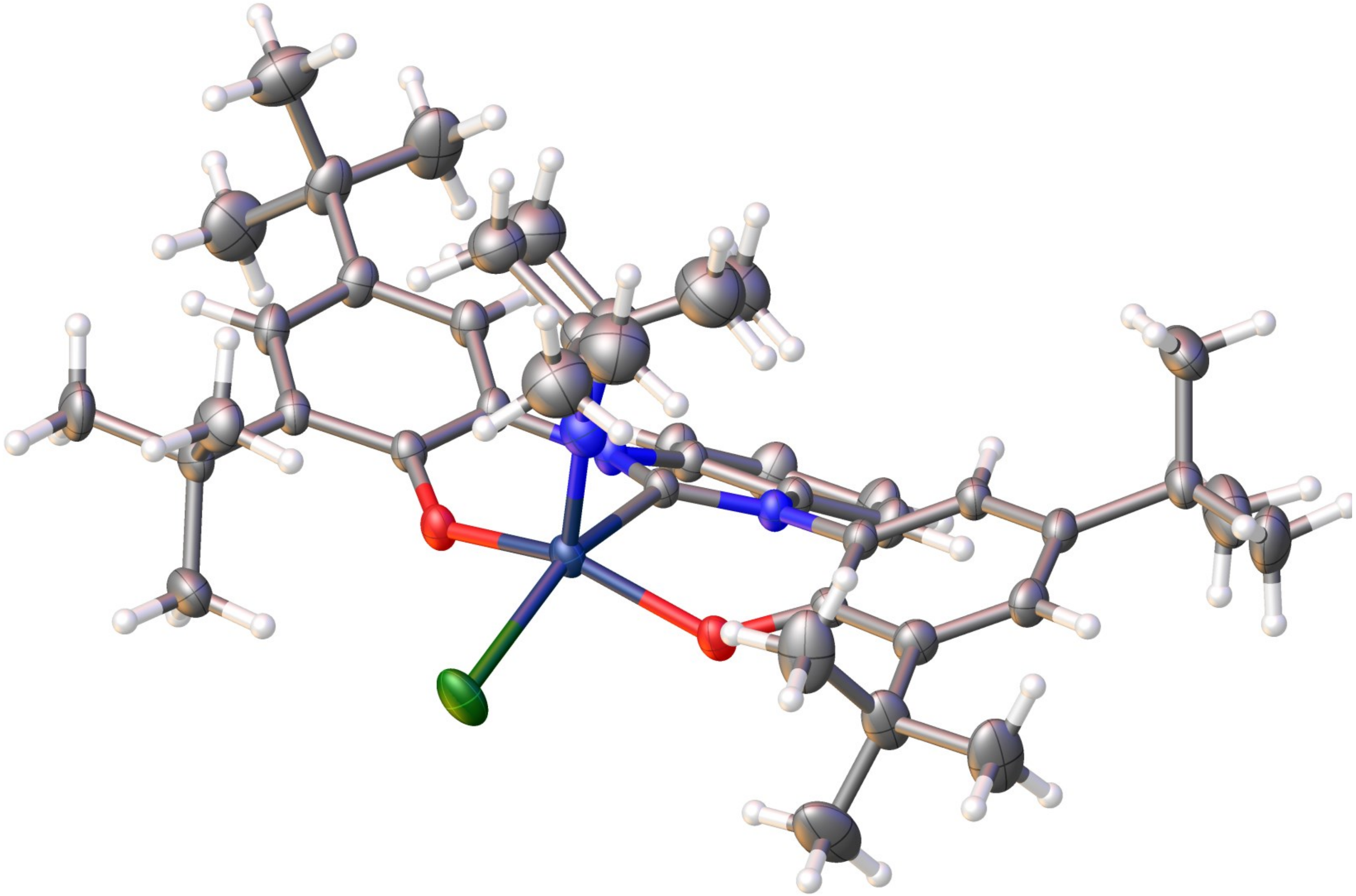

Figure S 73: Molecular structure of **1**. Ellipsoids are shown at a probability level of 50%.

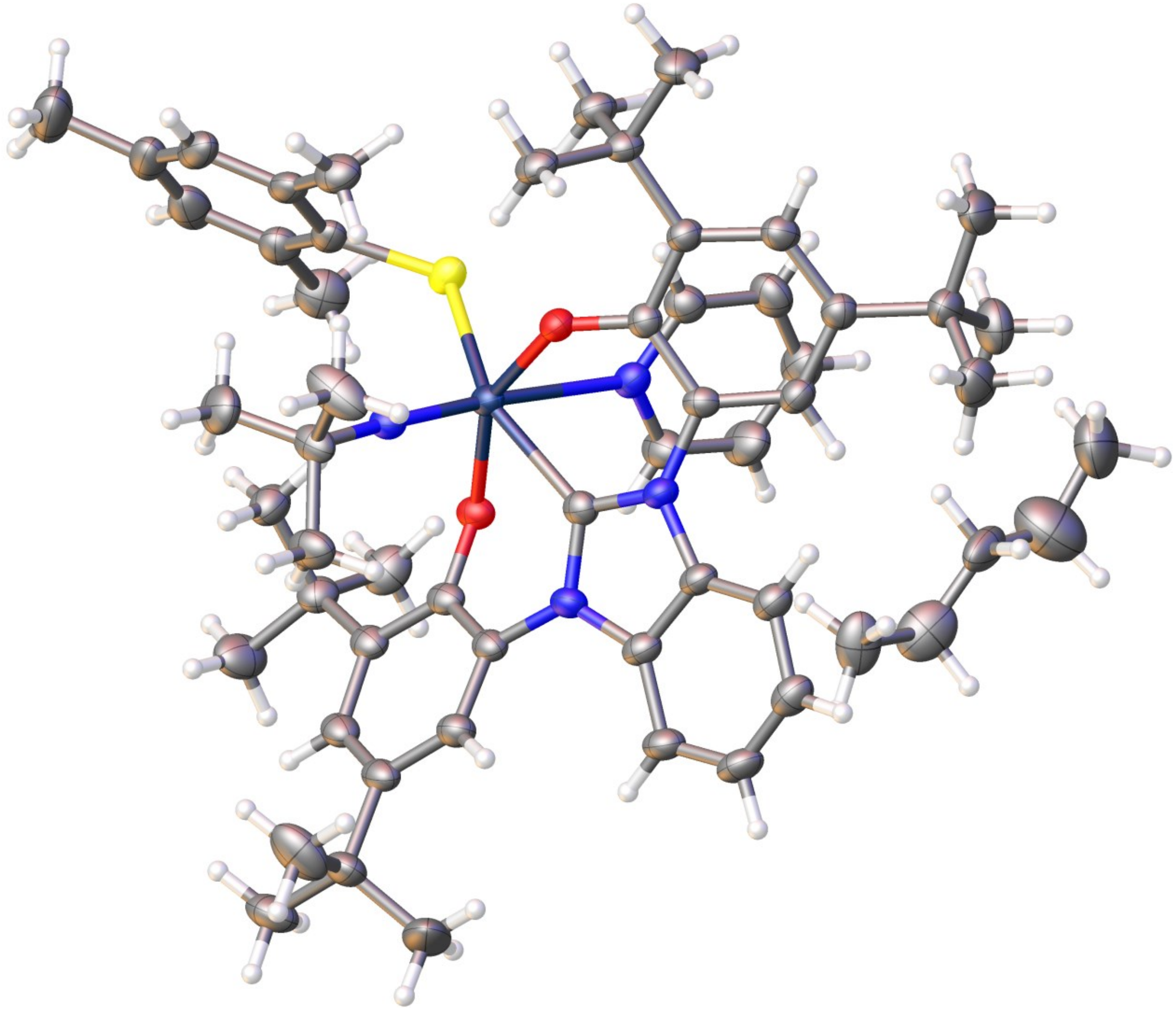

Figure S 74: Full Molecular structure of **3**. Ellipsoids are shown at a probability level of 50%.

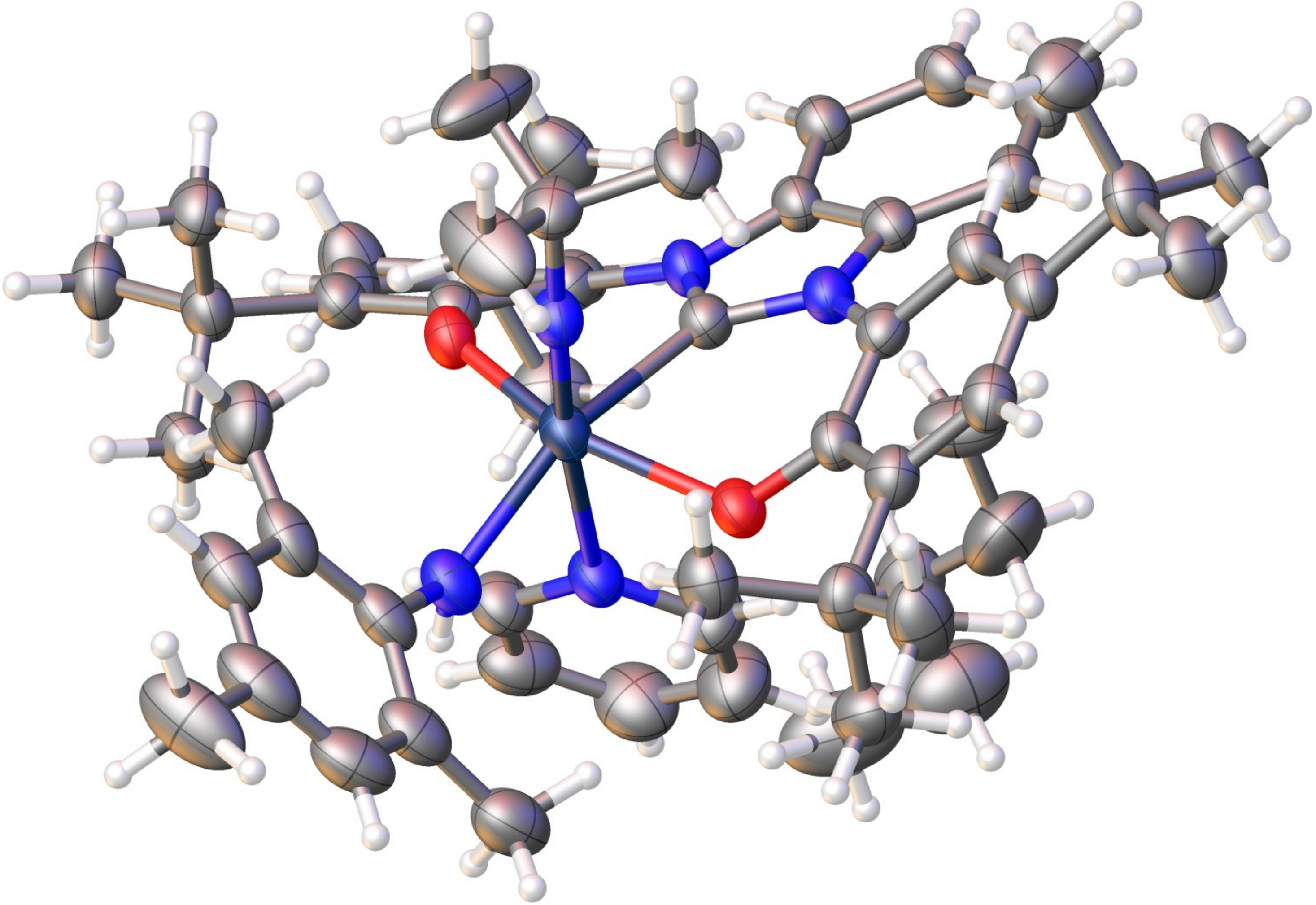

Figure S 75: Full Molecular structure of **4**. Ellipsoids are shown at a probability level of 50%.

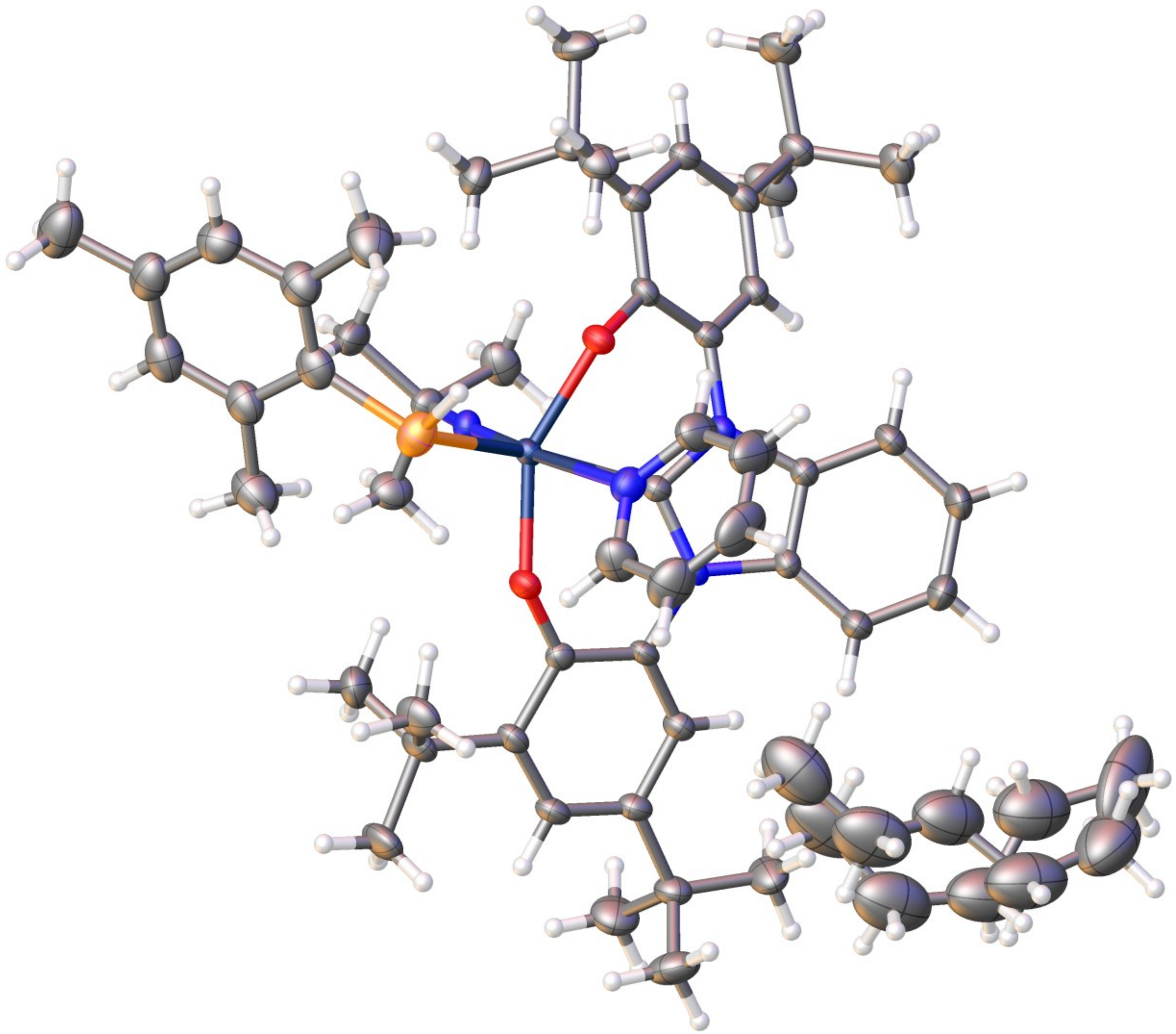

Figure S 76: Full Molecular structure of **5**. Ellipsoids are shown at a probability level of 50%.

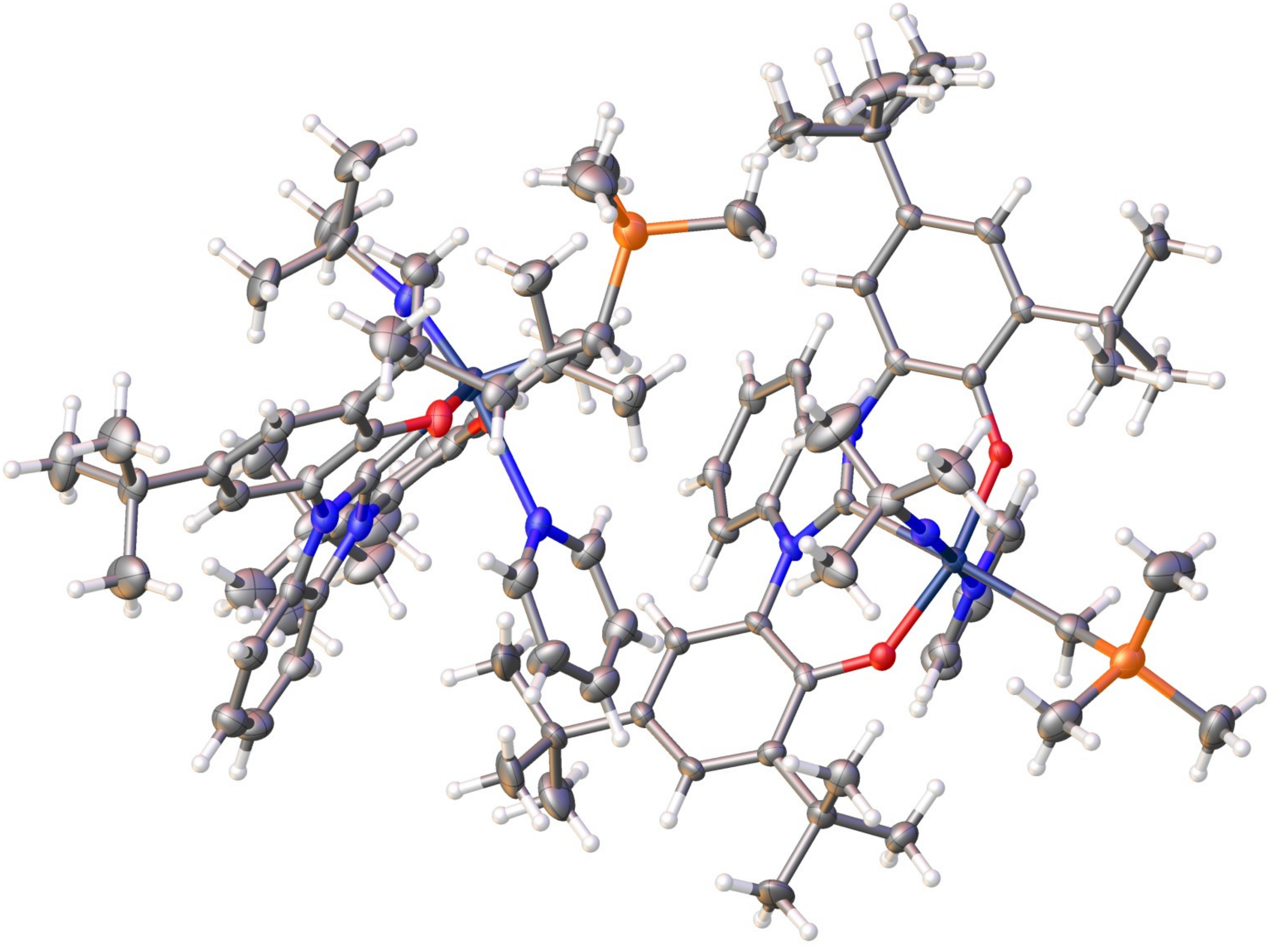

Figure S 77: Full Molecular structure of **6**. Ellipsoids are shown at a probability level of 50%.

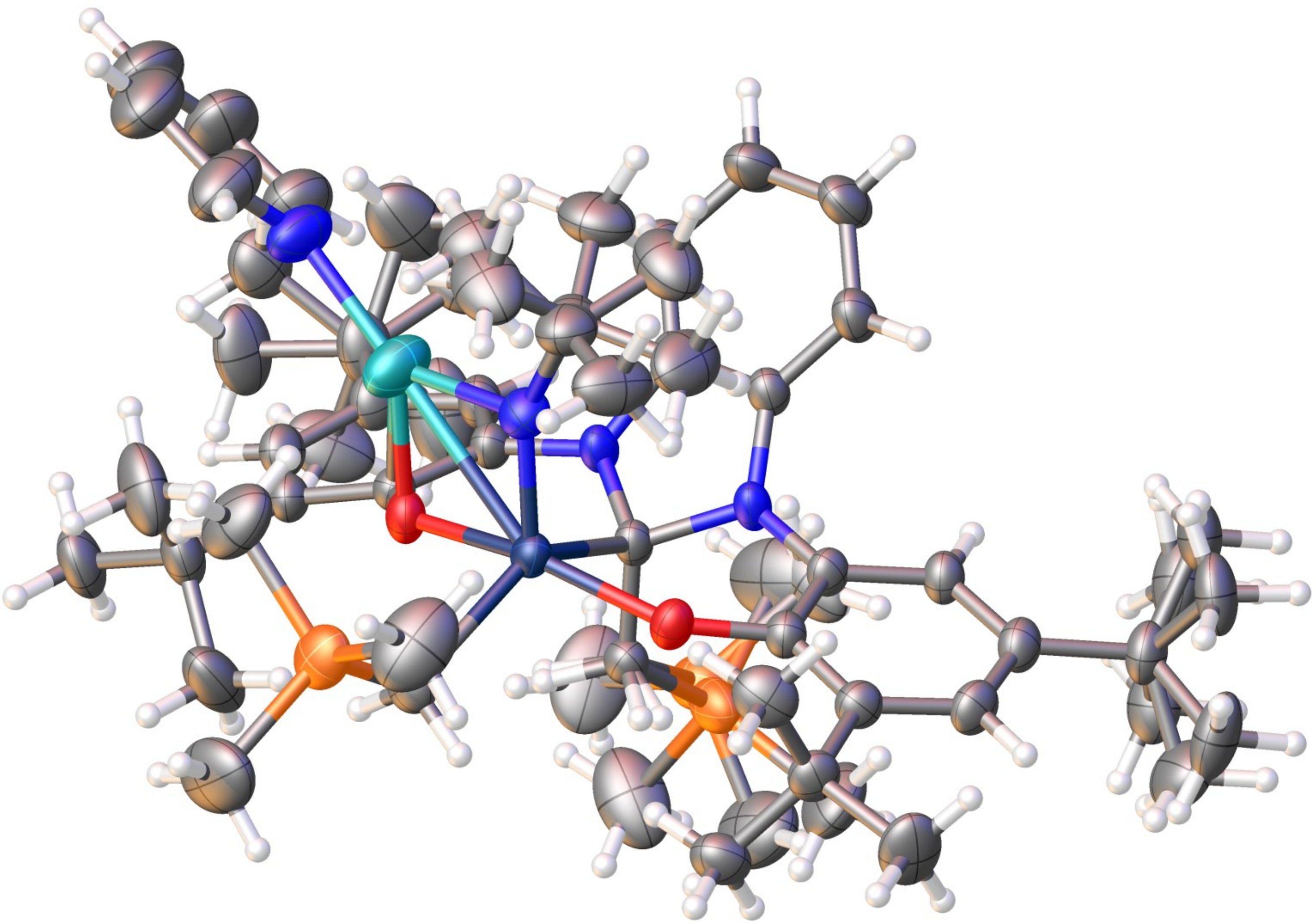

Figure S 78: Full Molecular structure of **7**. Ellipsoids are shown at a probability level of 50%.

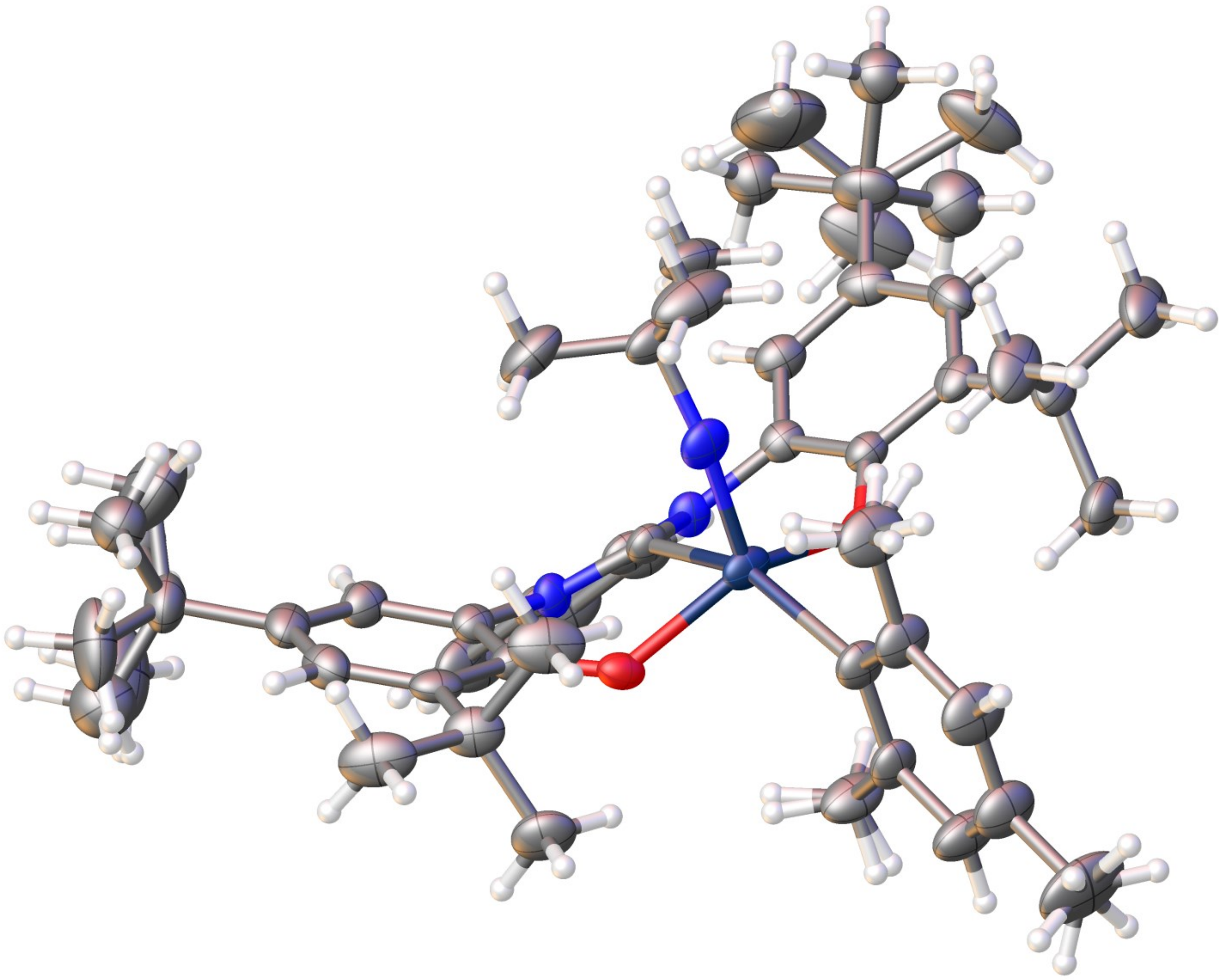

Figure S 79: Full Molecular structure of **8**. Ellipsoids are shown at a probability level of 50%.

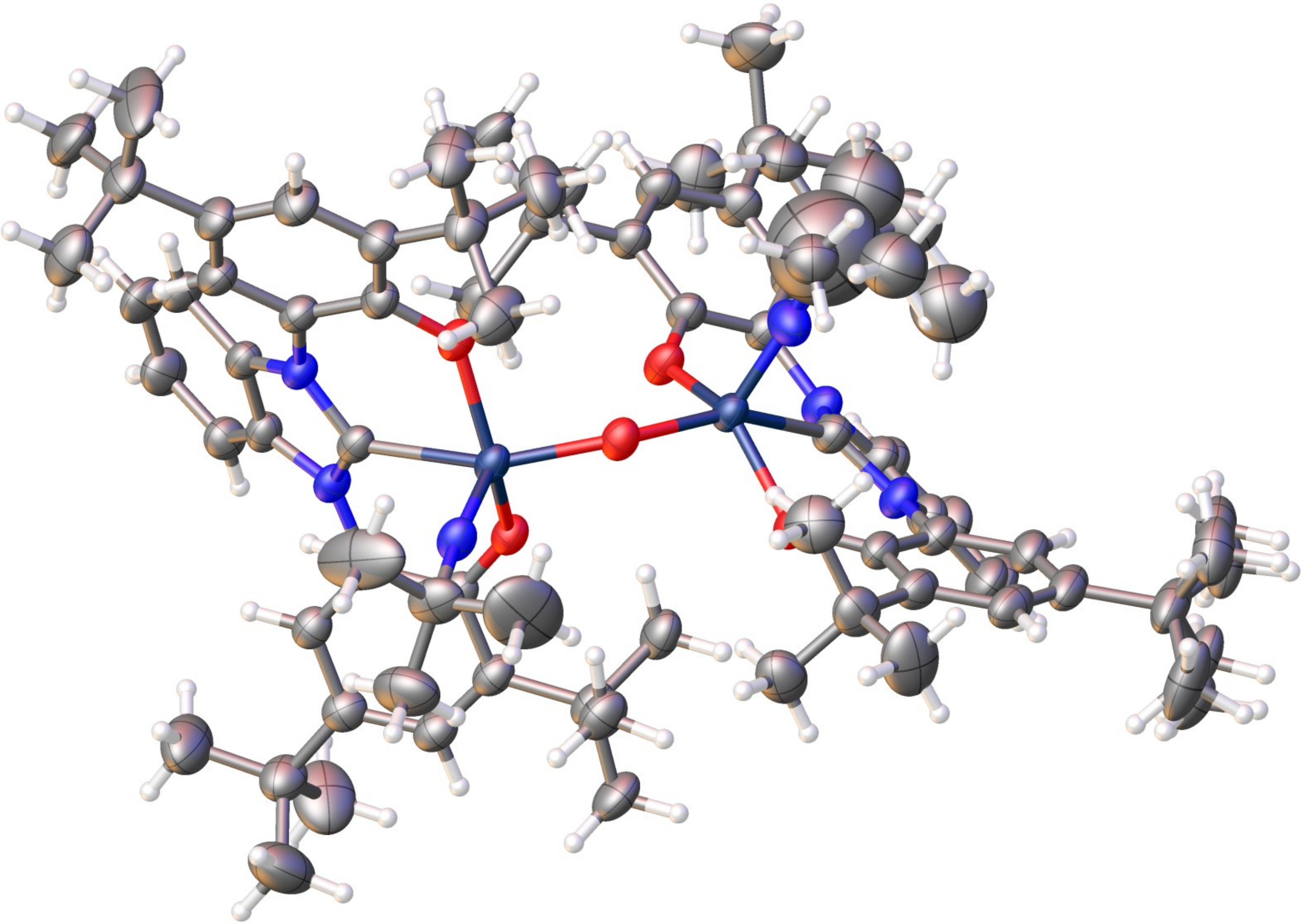

Figure S 80: Full Molecular structure of **9**. Ellipsoids are shown at a probability level of 50%

## 4. Electrochemistry (Cyclic Voltammetry)

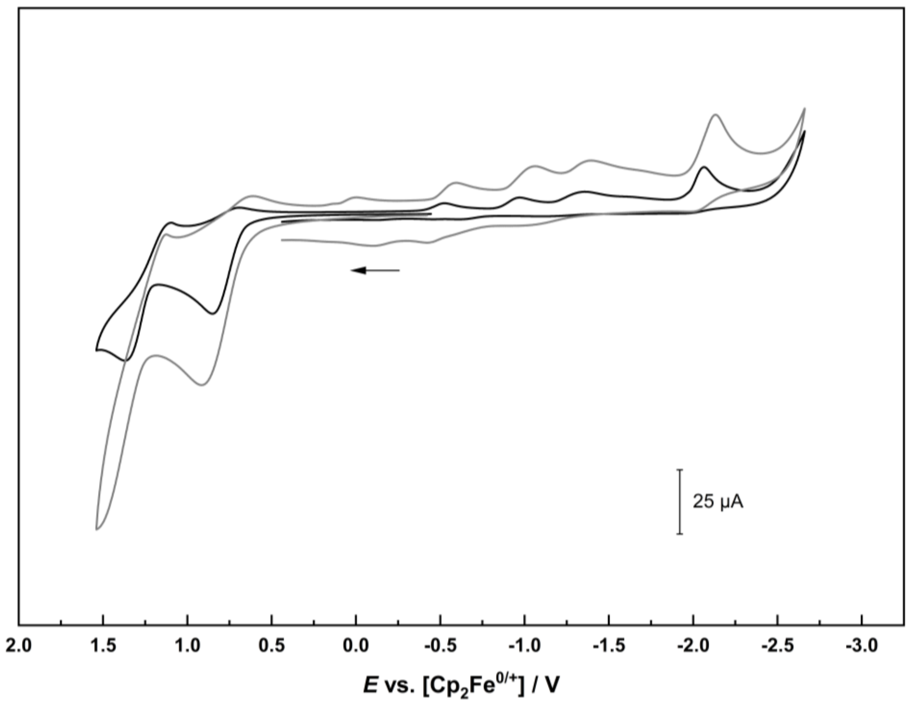


Figure S 81: Cyclic voltammogram of a 0.001 M solution of **1-Py** in a 0.1 M solution of $NBu_4PF_6$ in dichloromethane at 298 K at sweep rates of 0.100 V/s (black line) and 0.500 V/s (gray line). Pease note that all reductive processes result from the irreversible oxidative processes.

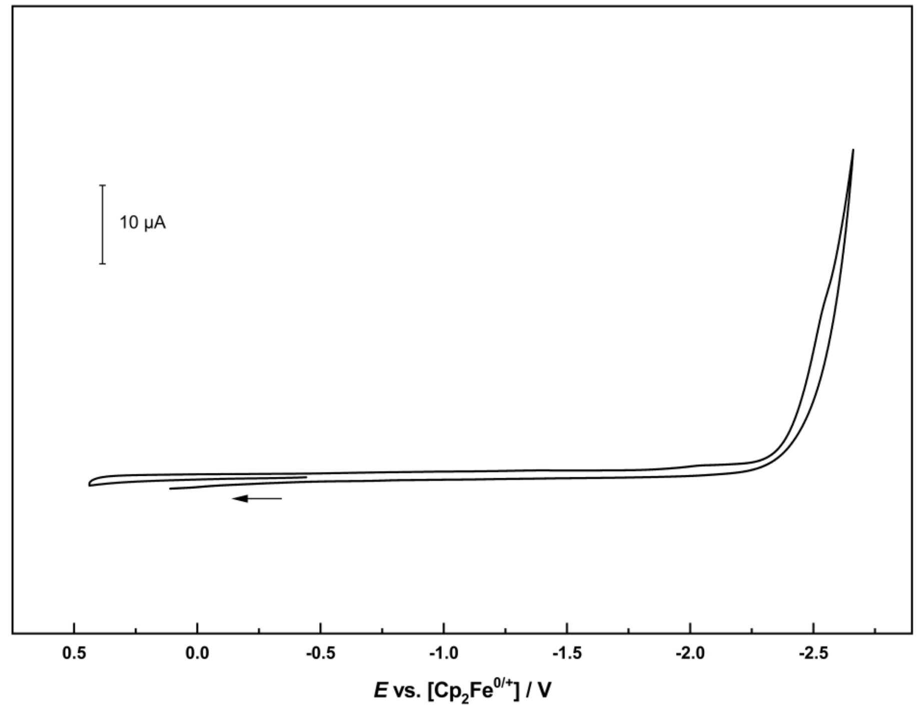


Figure S 82: Cyclic voltammogram of a 0.001 M solution of **1-Py** in a 0.1 M solution of $NBu_4PF_6$ in dichloromethane at 298 K at sweep rates of 0.100 V/s. Only the reductive scan is shown to show that all reductive processes observed in Figure S72 result from the irreversibility of the oxidation processes.

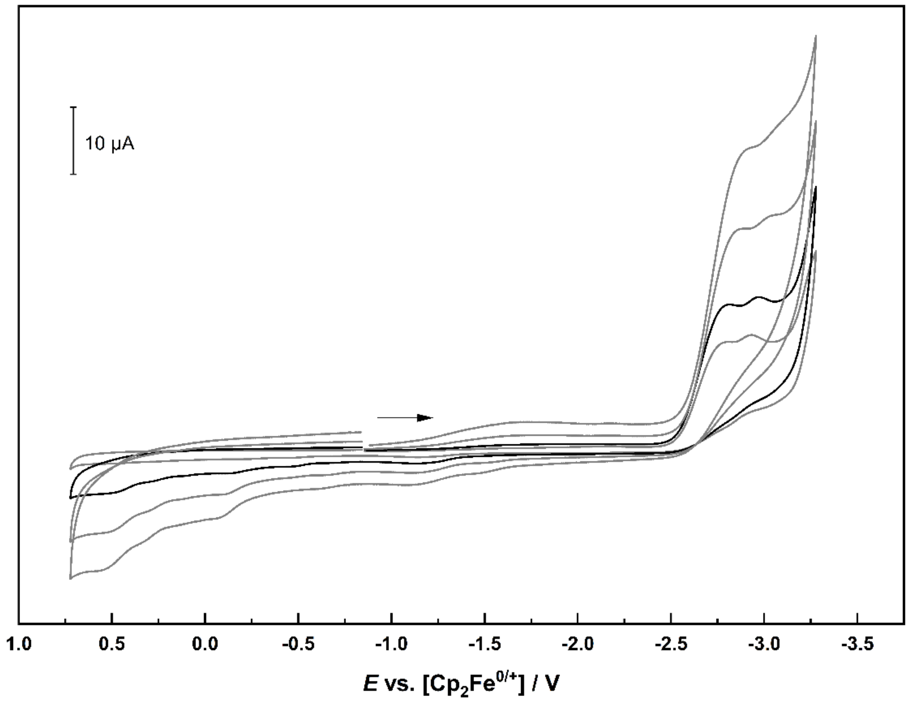


Figure S 83: Cyclic voltammogram of a 0.001 M solution of **1-Py** in a 0.1 M solution of $NBu_4PF_6$ in tetrahydrofuran at 298 K at sweep rates of 0.050 V/s, 0.100 V/s (black line), 0.250 V/s and 0.500 V/s.

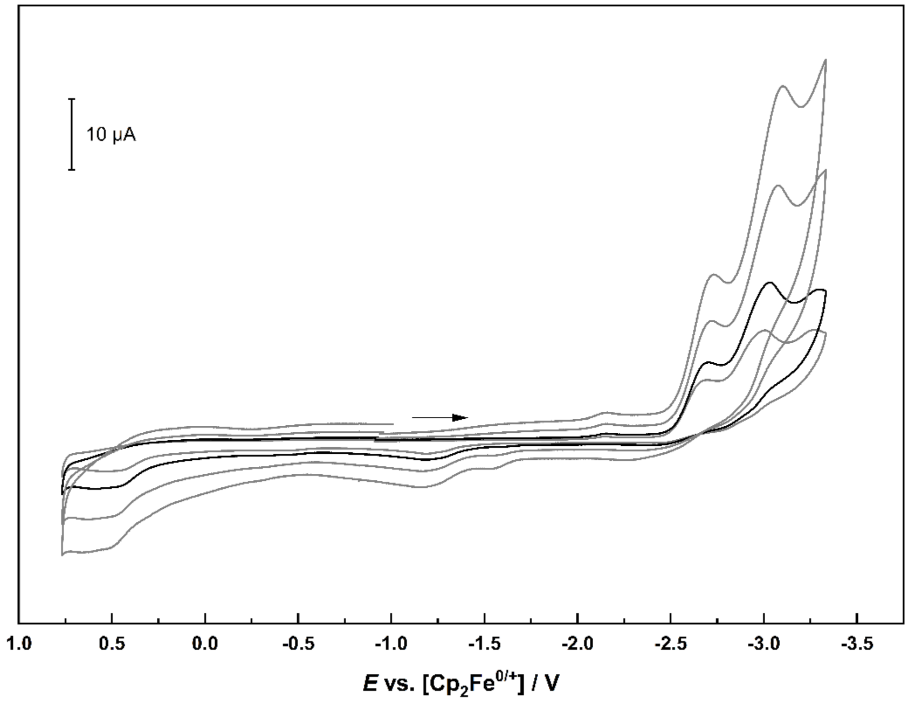


Figure S 84: Cyclic voltammogram of a 0.001 M solution of **1** in a 0.1 M solution of $NBu_4PF_6$ in tetrahydrofuran at 298 K at sweep rates of 0.050 V/s, 0.100 V/s (black line), 0.250 V/s and 0.500 V/s.

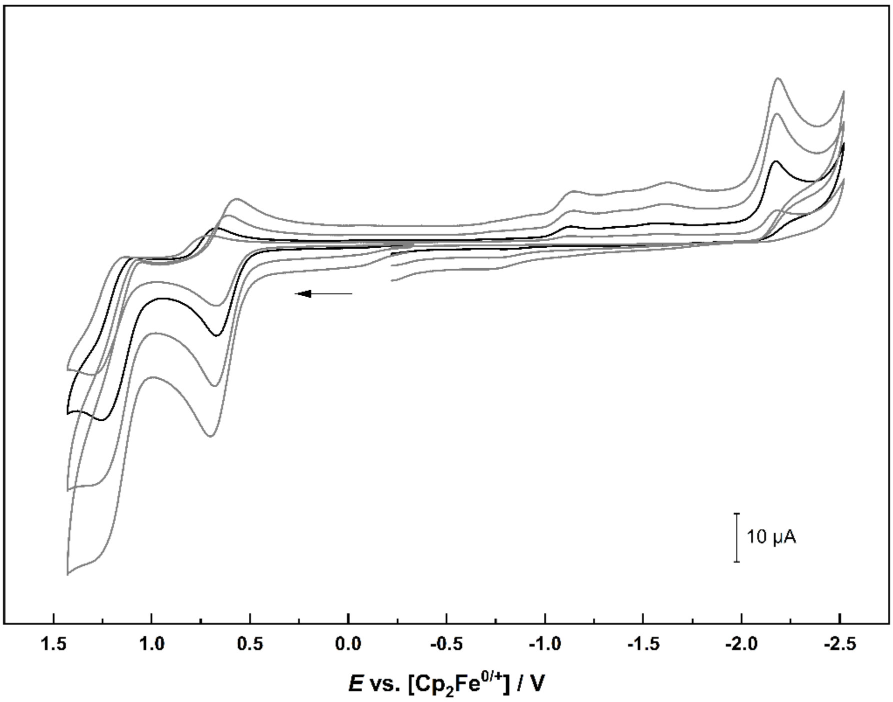


Figure S 85: Cyclic voltammogram of a 0.001 M solution of **complex I** (Figure 1 main manuscript) in a 0.1 M solution of $NBu_4PF_6$ in dichloromethane at 298 K at sweep rates of 0.050 V/s, 0.100 V/s (black line), 0.250 V/s and 0.500 V/s. All reductive processes result from the irreversible oxidative processes. For a separated scan of the reduction potentials, see below.

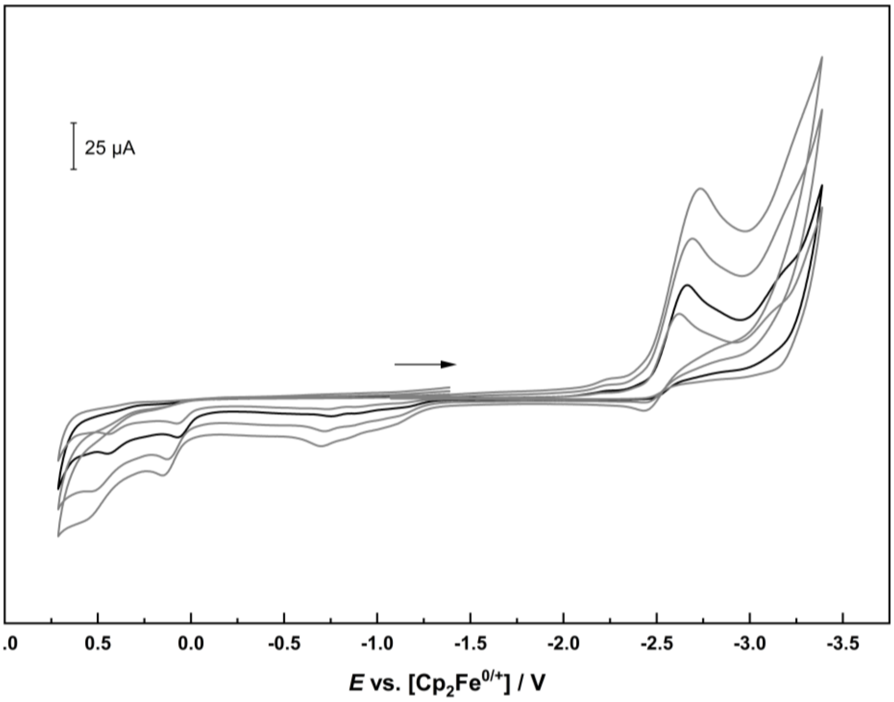


Figure S 86: Cyclic voltammogram of a 0.002 M solution of **complex I** (Figure 1 main manuscript) in a 0.1 M solution of $NBu_4PF_6$ in tetrahydrofuran at 298 K at sweep rates of 0.050 V/s, 0.100 V/s (black line), 0.250 V/s and 0.500 V/s. All oxidative processes result from the irreversible reductive processes.

Table S 3: Summary of electrochemical processes and corresponding potentials of the complexes **1-Py**, **1** and **I**.

| Compound | 1st oxidation $E_{pa}$ / V [a] | 2nd oxidation $E_{pa}$ / V [a] | 1st reduction $E_{pc}$ / V [b] | 2nd reduction $E_{pc}$ / V [b] |
|---|---|---|---|---|
| **1-Py** | 0.845 | 1.355 | -2.815 | -2.975 |
| **1-THF** | - | - | -2.701 | -3.034 |
| **I** | 0.676 | 1.258 | -2.649 | - |

[a] peak potential of an irreversible oxidation wave recorded at 0.100 V/s in 0.10 M $NBu_4PF_6$ in $CH_2Cl_2$, [b] peak potential of an irreversible reduction wave recorded at 0.100 V/s in 0.10 M $NBu_4PF_6$ in THF, [c] potentials were calibrated internally against the ferrocene/ferrocenium redox couple.
